# Lessons From the UK's Lockdown: Discourse on Behavioural Science in Times of COVID-19

**DOI:** 10.3389/fpsyg.2021.647348

**Published:** 2021-06-17

**Authors:** Jet G. Sanders, Alessia Tosi, Sandra Obradovic, Ilaria Miligi, Liam Delaney

**Affiliations:** ^1^Department of Psychological and Behavioural Sciences, London School of Economics and Political Sciences, London, United Kingdom; ^2^Independent Researcher, London, United Kingdom; ^3^School of Psychology and Counselling, Faculty of Arts and Social Sciences, The Open University, England, United Kingdom

**Keywords:** behavioural science, behavioural policy, COVID-19, national lockdown, trust in science, corpus linguistics, media discourse analysis, Twitter

## Abstract

In recent years *behavioural science* has quickly become embedded in national level governance. As the contributions of *behavioural science* to the UK's COVID-19 response policies in early 2020 became apparent, a debate emerged in the British media about its involvement. This served as a unique opportunity to capture public discourse and representation of *behavioural science* in a fast-track, high-stake context. We aimed at identifying elements which foster and detract from trust and credibility in emergent scientific contributions to policy making. With this in mind, in Study 1 we use corpus linguistics and network analysis to map the narrative around the key *behavioural science* actors and concepts which were discussed in the 647 news articles extracted from the 15 most read British newspapers over the 12-week period surrounding the first hard UK lockdown of 2020. We report and discuss (1) the salience of key concepts and actors as the debate unfolded, (2) quantified changes in the polarity of the sentiment expressed toward them and their policy application contexts, and (3) patterns of co-occurrence via network analyses. To establish public discourse surrounding identified themes, in Study 2 we investigate how salience and sentiment of key themes and relations to policy were discussed in original Twitter chatter (*N* = 2,187). In Study 3, we complement these findings with a qualitative analysis of the subset of news articles which contained the most extreme sentiments (*N* = 111), providing an in-depth perspective of sentiments and discourse developed around keywords, as either promoting or undermining their credibility in, and trust toward behaviourally informed policy. We discuss our findings in light of the integration of *behavioural science* in national policy making under emergency constraints.

## Introduction

Public trust in the transparency and reliability of scientific evidence is an important component of effective responses to major challenges and crises (Hendriks et al., 2015; Pittinsky, 2015). Generally, public perceptions of science are positive: science is often held in high esteem with equally high confidence placed in scientists (e.g., Scheufele, 2013; Jonge, 2015; National Science Board, 2016; Lamberts, 2017; Robert Bosch Stiftung, 2017; Lindholm et al., 2018). However, the application of science in policy has variable success (Sanchez-Paramo et al., 2019). Not all science is deemed fit to inform policy (Anvari and Lakens, 2018; Ioannidis, 2018; Cairney, 2020; Stevens, 2020). Determining when a scientific discipline is ready to inform policy is precarious and can be volatile and the criteria for evidence-readiness can vary depending on what is at stake (Ruggeri et al., 2020). In addition, policy choices are trade-offs shaped by many pressures other than those based on evidence. Direct competition from other pressures can shape public perceptions of the evidence and can steer the policy-makers' ability to implement evidence at hand (Cairney, 2020).

In March 2020, the UK was faced with the high stake policy choice of a national lockdown as COVID-19 spread globally (Kreps and Kriner, 2020). As scientific evidence about the virus and its effects was sparse, a broad range of scientists were called onto expert panels to advise governments. In British policy, unlike many other national governments, one prominent perspective was that of the *behavioural science* (UK Government, 2020).

The integration of *behavioural science* into UK policy took several forms, but was most notably embodied by (1) the inclusion of Dr. David Halpern, chief executive of the Behavioural Insights Team (BIT) in the government's Scientific Advisory Group for Emergencies (SAGE) and (2) the development of a behavioural advisory group known as the Scientific Pandemic Influenza Group on Behaviours (SPI-B). It is possible that *behavioural science* was particularly well-represented in the UK because it has been embedded in British policy for longer and more widely than in other national systems. The UK Cabinet Office was amongst the first to embed a dedicated *behavioural science* unit (often called the “nudge unit”) to that effect (Sanders et al., 2018). Arguably, it is in part due to this unit that the effect of nudges as a novel policy instrument (Lourenco et al., 2016) and methods to test for their effectiveness (Della Vigna and Linos, 2020) were demonstrable on national policy level and embedded elsewhere. We have since seen an increasing popularity for the policy approach, as evidenced by the growing number of behavioural insight units that advise national governments on issues involving citizen choices in the last 10 years (Whitehead et al., 2014; Halpern, 2015).

Despite these successes, in March 2020 the role of behavioural scientists in the UK's COVID-19 response was heavily debated in the media. This left the questions: what caused debate about the role of this emergent science, what were its consequences (if any) and how can we learn from the communication around its scientific contributions to this high-stake policy? While an emerging body of literature exists on support for behavioural interventions (e.g., Reynolds et al., 2019, Sunstein et al., 2019), far less work has been conducted on trust in behavioural scientists more generally, and no work that we are aware of examines public support for the inclusion of behavioural scientists in committees advising government and shaping policy.

To examine this, we were particularly interested in this debate in media discourse, given media's important role in forming public opinion and therefore setting public trust in emerging science (Van Aelst, 2014). In addition, mass media plays an important agenda-setting role: it can direct collective attention and perceived importance (McLeod et al., 1974), shape how severe an issue is perceived to be, and influence how individuals come to perceive their social and political environment (Tyler, 1980; Protess and McCombs, 2016). In other words, mass media play an important “mediating” role in sharing and shaping how scientific and political expertise is understood by the public (Baum and Potter, 2008; Kim et al., 2018).

The rise of social media platforms such as Twitter in recent years (Gil de Zúñiga et al., 2012) provides a further opportunity to consider how public perceptions are then reflected by news media. For example, Chew and Eysenbach (2010) show that during the H1N1 pandemic, individuals used Twitter to share resource-related posts, with news media websites being the most popular sources to share. But social media users do not only reproduce existing information, they are also actively engaged in how key debates are shared and understood, with the potential to impact decision-making in significant ways (Bello-Orgaz et al., 2017). Twitter gives rise to a huge volume of text-based data, with over 500 million “tweets” generated by users each day (Chae, 2015; Mention, 2018). These tweets can be useful for tracking how public opinion develops around key social issues (D'Andrea et al., 2019). Specifically, relevant for the present context, Twitter data has been used to analyze public opinion on vaccination (Bello-Orgaz et al., 2017; D'Andrea et al., 2019), the role of fake news during the pandemic (Gruzd and Mai, 2020) and shifts in public emotions during the pandemic, from fear to anger (Lwin et al., 2020). This emerging research highlights the role of media (both traditional and social) in providing key information, focusing public attention on social issues and shaping public opinion and emotions. We build on this literature in our paper.

This paper provides a key case study on trust and acceptability surrounding the contributions of social and *behavioural science* in times of crises. Specifically, the paper aims to address the following questions: How was this emergent science debated in the print media, and what can we learn from its perceived credibility in informing policy? To answer these questions, we examine public and media discourse surrounding the high-stake policy decision of the first national lockdown in the UK in March 2020. We draw on a 24-week period to track the discourse as it evolved. Specifically, our case study draws on two independent sources: In Study 1 we use print media to examine the salience, sentiment and co-occurrence of *behavioural science* keywords in the media. In Study 2, we draw on Twitter data to track how this print media discourse is picked up and appropriated in public discourse. Lastly, in Study 3, we draw on a subset of newspaper articles from Study 1 to provide a more in-depth analysis of how discourses around trust and credibility of *behavioural science* are constructed and either promoted or undermined.

In bringing together the findings from the three studies, a key objective of this paper is to understand what the consequences (if any) of this discourse were, and how we can learn from it to further trust toward scientific contributions to high-stake policy.

## Study 1 Newspaper Discourse Analysis

Top newspapers have been shown to sway common understanding of scientific disciplines and can be used as a proxy to measure understanding of their place in public policy (Bauer et al., 1994; Mutz and Soss, 1997; Schäfer, 2012). As the contributions of *behavioural science* to the UK's COVID-19 lockdown policies developed, and debate emerged in the British media about its involvement, we reasoned that, in the lead up to, during and after the UK COVID-19 lockdown period in March 2020, public perceptions of *behavioural science* contributions to this high-stake UK policy decision should be detectable from newspaper articles. With this in mind, we set out to explore (1) the prevalence of *behavioural science* actors and concepts in relation to national level policy making, (2) the valence associated with such actors and concepts (see Alamoodi et al., 2020 for a review of its use in other COVID-19 policy contexts), and (3) the co-occurrence of key *behavioural science* concepts and actors over the lockdown period of 2020.

### Materials and Methods

#### Materials

We retrieved news articles from the online database *Lexis Nexis* for an 8-week window on either side of the hard UK lockdown (27th of January 2020–10th of July 2020). We drew on the 15 UK newspapers with the highest circulation levels (see Supplementary Material 1). We estimate that articles in these newspapers collectively reached almost 8 million people in print and in digital editions (~12% of the British population) on a monthly basis (Mayhew, 2020; Worldometer, 2020).

**Figure 1:**
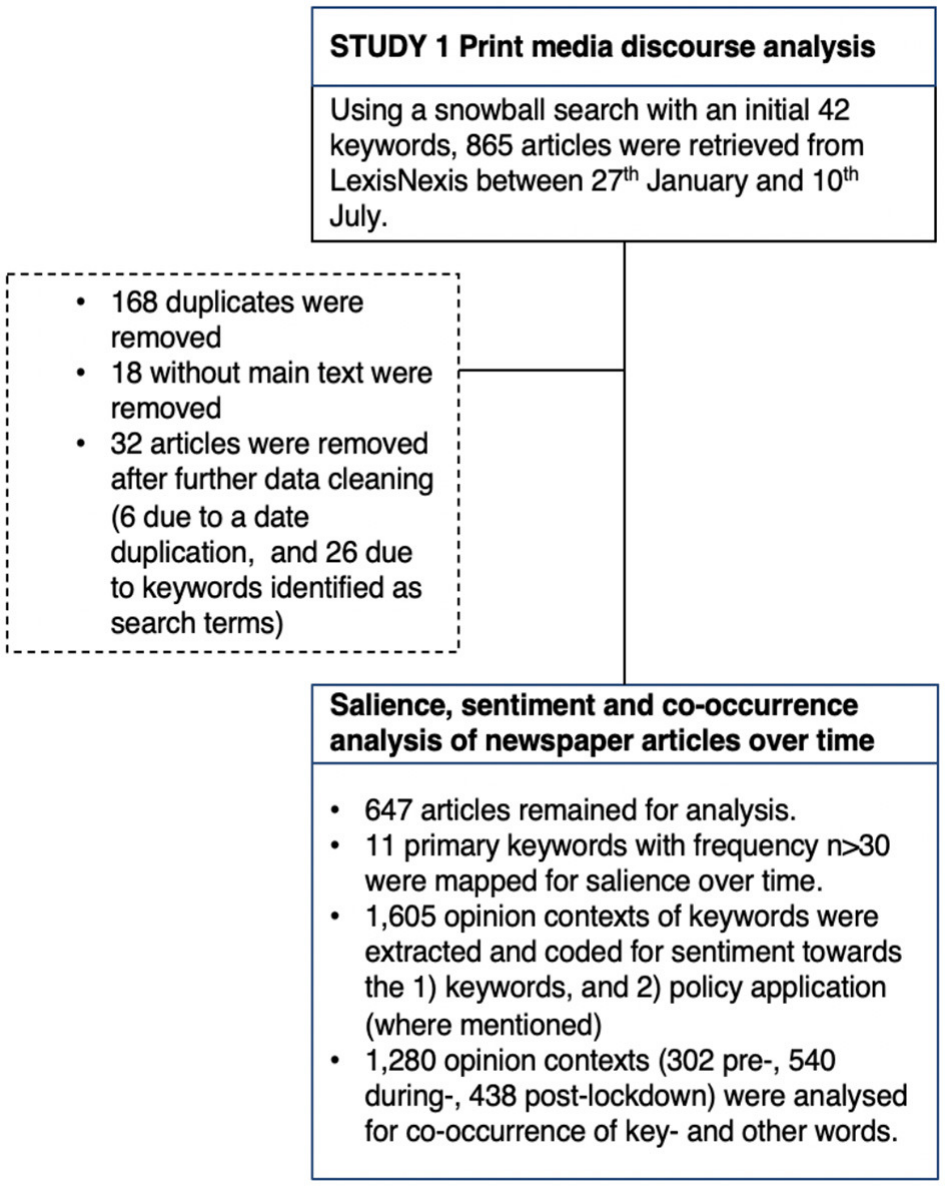
Flowchart of data selection and cleaning process for Study 1.

Using a snowball method, we developed a query to identify articles relevant to the discussion of *behavioural science* (see Supplementary Material 2A for the various stages and final query). The search produced a corpus of 865 articles. Deduplication and removal of incomplete articles resulted in a sample of 679 articles. These were qualitatively reviewed by three coders for relevance to the topic of *behavioural science*. This left 647 articles (ranging from 1 to 47 per news outlet; see Supplementary Material 1 for details) for the quantitative analysis (see Figure 1).

#### Keyword Processing

We defined an initial set of 42 keywords based on the snowball method applied through the search query (see Supplementary Material 3 for a complete set). As one word can be expressed in different ways (e.g., abbreviated, singular/plural form, or by use of synonyms), keywords were grouped to form primary keywords as follows: (1) plurals were standardized into a singular form: e.g., *behavioural science* and *behavioural science* as *behavioural science*; (2) synonyms were unified: e.g., *nudge unit* and *Behavioural Insights Team* as *Behavioural Insights Team*; and (3) we integrated semantically related keywords based on expert knowledge: e.g., *nudge, nudging, nudge theory*, and *nudge strategy* were noted as *nudge*. As exceptions to this rule we kept *psychologist, behavioural scientist*, and *behavioural economist* as stand-alone primary keywords. As profession names often preface unique actors (as opposed to their plural counterparts; e.g., Professor of Health Psychology Susan Michie VS Professors at Oxford), they lend themselves as proxies for actors not captured in our keyword base^1^.

This resulted in 20 primary keywords: *behaviour science*, affiliated disciplines (*psychology, behaviour economics*), *behavioural science* concepts (*nudge, choice architecture, irrational behaviour, behaviour change, behavioural analysis, behavioural insights*), commonly named actors in national or international behavioural policy work (*SPI-B, Behavioural Insights Team, Michie, Halpern, Chater, Thaler, Sunstein, Kahneman*), and unnamed *behavioural science* actors (*behavioural scientist, psychologist, behavioural economist*).

#### Analyses

##### Salience

To assess the salience of primary keywords over time, we first removed all “parts-of-speech” apart from nouns or keywords. This is based on the assumption that it is nouns that are the part of speech that represent the content of an article (Stuart et al., 2013). A salience score was calculated for each primary keyword for every two-week period. The score was a product of the keyword's normalised corpus frequency (i.e., number of keyword occurrences divided by total word counts per 10,000 words) and the keyword's relative document frequency (i.e., proportion of articles in which the keyword was mentioned). This composite metric allowed us to account both for the centrality of a keyword in the narrative of the articles published in the 2-week period (normalized corpus frequency), and the spread of the keyword in the media in the same period (relative document frequency; Manning et al., 2008).

##### Sentiment

Targeted sentiment analysis was used to assess perceptions of *behavioural science* applied in national public policy context. We first identified all sentences (*N* = 1280) in our corpus where a *behavioural science* keyword occurred. As a sentence could contain more than one keyword (median = 1, range = 1–5), this resulted in a sample of 1,605 keyword-sentence pairs, termed opinion contexts. Each opinion context was coded manually for sentiment polarity expressed toward the keyword on a 5-point scale: −2 (extremely negative), −1 (moderately negative), 0 (neutral), +1 (moderately positive), +2 (extremely positive). Opinion contexts were also reviewed to contain reference to national-level policy (e.g., mention of *government, minister, no. 10*, see Supplementary Material 3 for a full list). When this was the case, sentiment polarity toward the policy actor linked to *behavioural science* was also rated. A subset of 110 opinion contexts were coded by all three coders to produce an inter-rater agreement and solve cases of disagreement, with the remainder coded by single coders (Hallgren, 2012).

To match salience and sentiment scoring, results were presented for two-week intervals over the period of the first national lockdown of 2020 in three sentiment categories: negative (−1; −2), neutral (0) and positive (+1; +2).

##### Co-occurrence

Finally, we used co-occurrence network analysis to investigate how the conceptual structure of the public narrative around *behavioural science* evolved over the period of the first national lockdown (Corman et al., 2002; Paranyushkin, 2011). To allow for reasonable variance in co-occurrence, we opted to move from two-week windows to a pre-, during- and post-lockdown window of analysis.

To analyze the narrative around keywords in the relevant context, we calculated co-occurrence at opinion-context level (*N* = 302 pre-, *N* = 540 during-, *N* = 438 post-lockdown) for two keywords or a keyword and any other term appearing in the same sentence. We expressed co-occurrence *via* the Dice coefficient: the ratio between the co-occurrence of two keywords and the sum of their individual occurrences multiplied by two (Frakes and Baeza-Yates, 1992; see Supplementary Material 6 for details). Simply put, two keywords that never co-occur have a coefficient of 0 and two keywords with identical occurrence have a coefficient of 1^2^.

In the co-occurrence networks, nodes represent terms and an edge between two nodes indicates the terms' co-occurrence, with a weight proportional to the strength of their association (i.e., the Dice coefficient) (Liu et al., 2012; Katsurai and Ono, 2019; Paranyushkin, 2019; Kim et al., 2020; Puerta et al., 2020). By graphically representing patterns of co-occurrence between terms, co-occurrence networks identify the importance of terms and their inter-relatedness (Van Eck et al., 2006; Van Eck and Waltman, 2007). The network analysis was conducted using the Python NetworkX 2.5 library (Hagberg et al., 2008).

To calculate co-occurrence, the opinion contexts were pre-processed as follows to reduce noise interference (cf. Véronis, 2004; Jurgens, 2011; Kim et al., 2020). (i) Common two- and three-word phrases that did not involve any of our behavioural-science keywords, were replaced with the corresponding bigrams/trigrams (e.g., “public health” with “public_health”) based on collocation statistics across all 647 articles, using Python gensim's Phrases model (Rehurek and Sojka, 2010). (ii) We only retained terms (beyond our keywords), whose part-of-speech was either adjective or noun. (iii) We removed terms which appeared less than 20 times across all three time windows (occurrence: median *N* = 1, Interquartile Range = 2, range = 1–223). Of 3,709 unique terms, 3,635 were eliminated and 74 were retained (appearing in 648 pre-, 1,470 during-, and 1,432 post-lockdown opinion contexts). (iv) The Dice coefficient was calculated between pairs that included at least one *behavioural science* keyword and with a raw co-occurrence of at least 10 in that time window.

To understand how the relevance of, and narrative around the keywords evolved, we calculated and compared the following network- and node-level metrics (Sudhahar et al., 2015):

(a) *Network density*: the ratio of the actual number of links between keywords to the maximum possible number of links. On a scale from 0 to 1, higher value indicates a cohesive network.
(b) *Network average clustering coefficient:* the interconnectedness of nodes in a network on a scale from 0 to 1. If more terms co-occur with each other, the clustering coefficient is high (it is 1 if every node is connected to all other nodes). If terms do not co-occur with each other, the clustering coefficient is low.
(c) *Node weighted degree centrality*: the sum of the edge weights for edges incident to that keyword. Higher values indicate more frequent direct links to other keywords.
(d) *Node weighted between centrality*: the degree to which a keyword stands between others. Higher values indicate greater importance in bridging subsets of keywords.
(e) *Communities:* we used the Louvain algorithm to detect communities of co-occurring words, i.e., “thematic clusters” in our networks (cf. Williams et al., 2016; Lozano et al., 2019). The Louvain algorithm works by maximizing modularity (Blondel et al., 2008). Modularity measures the density of connections within communities compared to the density of connections between communities. It takes on values between−1 and 1, with a higher value representing better community definition (Newman and Girvan, 2004). The Louvain algorithm has been found to be one of the fastest and best performing community-detection algorithms in comparative analyses (Lancichinetti and Fortunato, 2009; Yang et al., 2016).

Finally, the changing trends in keywords' relevance and narrative were identified by comparing the detected communities and ranking of keywords for node centrality metrics (c) and (d) in the three different time periods.

### Results

From all analyses we excluded 9 keywords due to extremely low overall frequency (<30 occurrences over the 24-week period of interest; see Supplementary Material 5A for details) as they did not provide enough data points across time to determine trends in our metrics of interest. This left 11 primary keywords: *behavioural science*, the discipline terms *behavioural economics* and *psychology*, four of the eight named actors (*Behavioural Insights Team, Halpern, Michie*, and *SPI-B*), two of three unnamed actors (*behavioural scientist* and *psychologist*), and two of six concept terms (*behaviour change* and *nudge*).

Below, we present trends in salience and sentiment toward behavioural-science keywords over time, followed by reference and sentiment toward public policy application and co-occurrence. As frequently the case in descriptive exploratory studies of linguistic data (e.g., Bian et al., 2016; Kim et al., 2020; Sharma et al., 2020), we contain our results to descriptive findings.

#### Salience and Sentiment of Keywords Over Time

**Figure 2:**
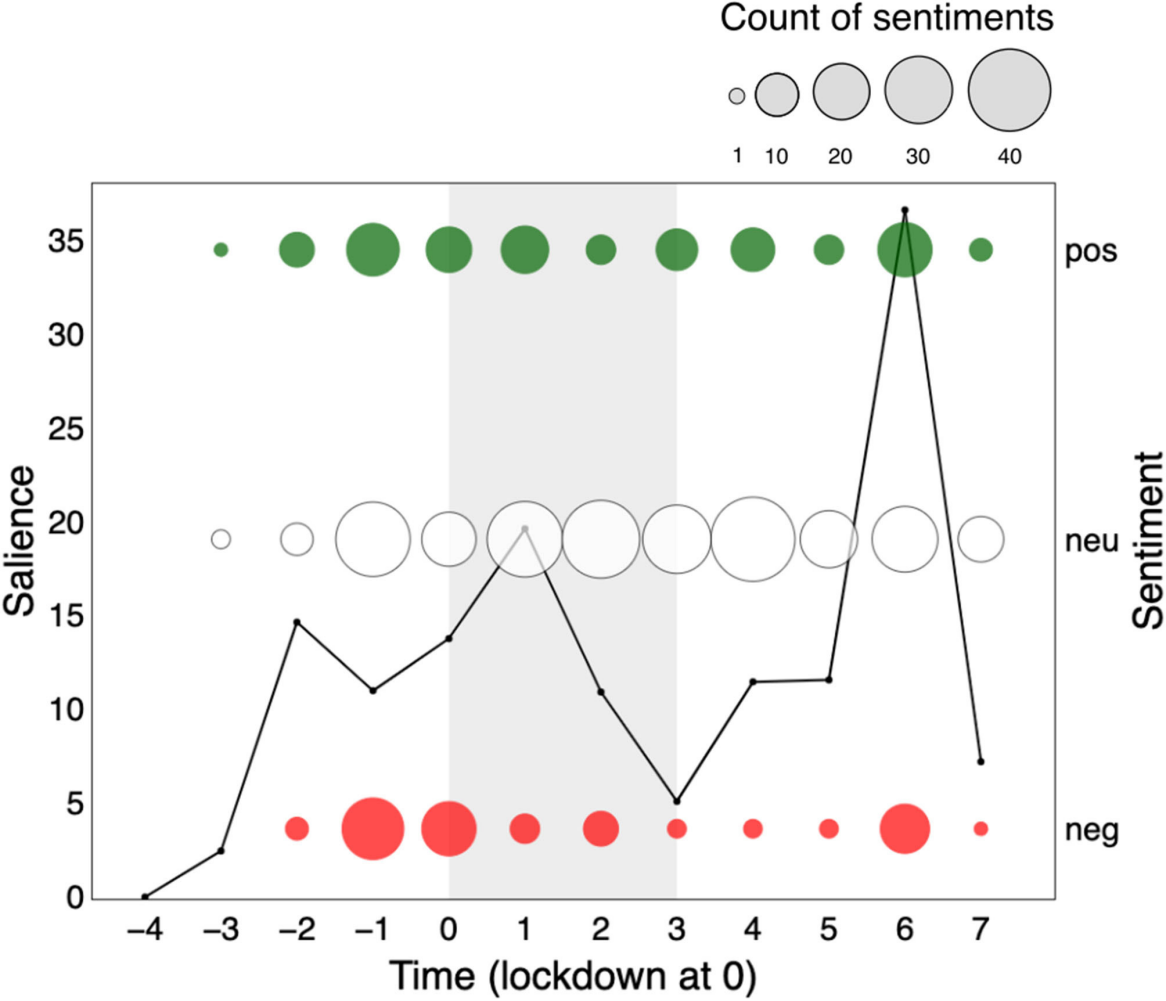
Salience of and sentiment toward the keyword “*behavioural science*” over a 12 two-week time-period surrounding the first British national lockdown of 2020 (gray area) in print media (top 15 UK newspapers). Salience is calculated for a 2-week period as the normalized keyword frequency (per 10,000 words) multiplied by the proportion of articles that mention the keyword. Sentiments are represented in counts of positive (+1 or +2), neutral (0), and negative (−2 or −1) bubbles over time, in green, white and red respectively. The size of the bubble is proportional to the count of sentiments in that polarity class toward the keyword.

Primary keyword *behavioural science* showed two clear surges: the first started one month prior to the UK lockdown (−2) and ended just after lockdown (+1) and the second rather spike-like surge occurred within a two-week period one month after the “hard” UK lockdown measures eased (+6; see Figure 2 and Supplementary Material 4). Simultaneous to the surges, we see an increase in polar sentiments: positive and negative sentiments are greater during these periods compared to other time-periods. This pattern is reminiscent of one commonly reported: “conflict” is deemed of news value and determines the extent to which journalists pay attention to politics (Galtung and Ruge, 1965; Harcup and O'neill, 2001; Van der Pas and Vliegenthart, 2016).

What seems to associate with the observed divisiveness? Discipline terms and unnamed actors do not show similar sentimental surges. *Psychology* (Figure 2I) seems to show a subdued version of *behavioural science* salience, with notably greater positive than negative sentiment. *Behavioural economics* is in fact largely absent from the narrative, with minimal salience in newspaper articles, but stable polarity over time.

**Figure 3:**
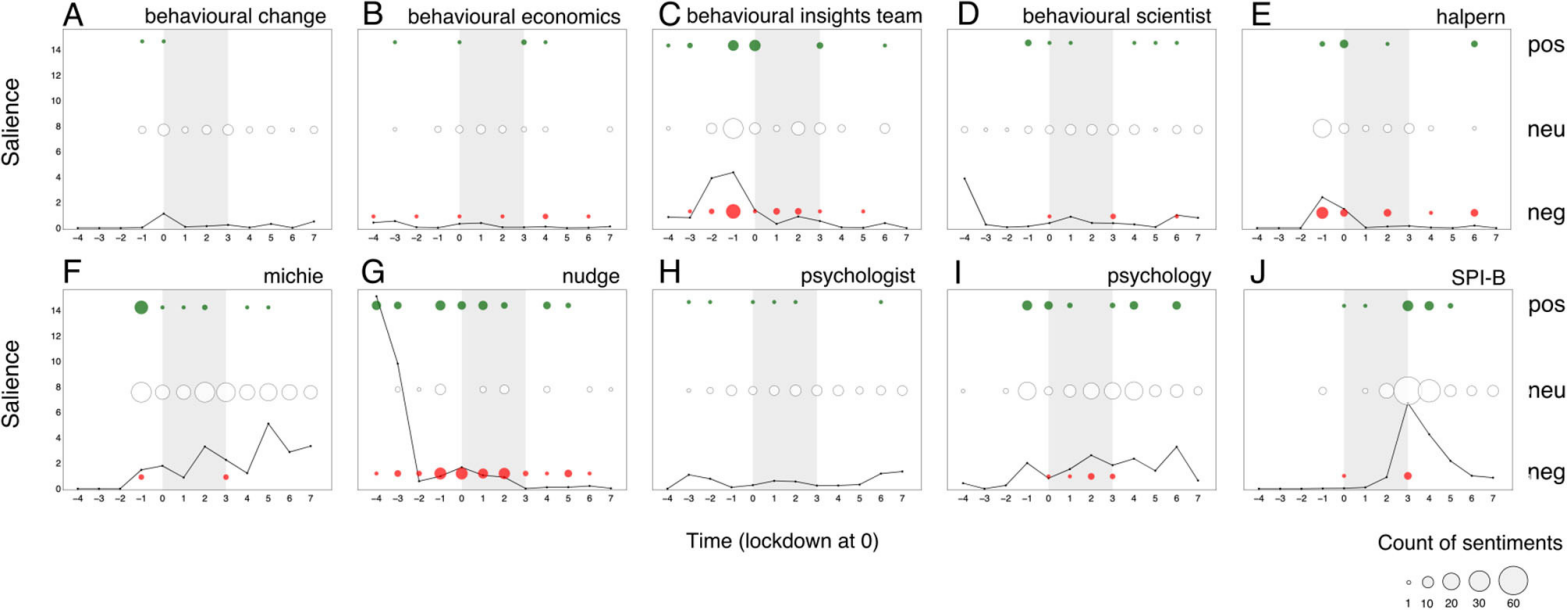
Salience of and sentiment toward the 10 primary keywords over the 12 two-week time period surrounding the first British national lockdown of 2020 (gray area) in print media (top 15 UK newspapers). **(A)** behaviour change (concept), **(B)** behavioural economics (discipline), **(C)** behavioural Insights Team (named actor), **(D)** behavioural scientist (unnamed actor), **(E)** halpern (named actor), **(F)** michie (named actor), **(G)** nudge (concept), **(H)** psychologist (unnamed actor), **(I)** psychology (discipline), **(J)** SPI-B (names actor). Salience is calculated per 2-week period as the normalized term frequency (per 10,000 words) multiplied by the proportion of articles that mention the keyword. Sentiments are represented in counts of positive (+1 or +2), neutral (0), and negative (−2 or −1) bubbles over time, in green, white and red respectively. The area of the bubbles is proportional to the count of sentiments toward the keyword.

Similarly, unnamed actors, such as *psychologist* (Figure 3H) or *behavioural scientist* (Figure 3D) do not share the surges in sentiment polarity observed for *behavioural science*. Although unnamed actors show a slight uptick in salience, they show a relatively steady (mostly neutral) sentiment.

We reach a different conclusion for named actors and concept terms. Salience for keyword *Michie* also mimics the *behavioural science* trend over time in subdued form, but with positive polarity during the first surge (−1). Keywords *Halpern* and *Behavioural Insights Team* show a nearly identical rise in salience to *behavioural science* in the period leading to lockdown, but rather eliciting a negative response. All actors thus seem to associate with the divisiveness we observe, possibly holding opposite perspectives. This narrative finds support in that all three actors only seem to emerge as public figures of *behavioural science* only around this pre-lockdown time period (−1).

A final pattern of divisiveness is aligned with the keyword *nudge*. Although *nudge* was not nearly as salient as other primary keywords, we observe negative sentiment during the first surge. In fact, *nudge* is the only primary keyword which, throughout the 24-week period, attracted more divisiveness than neutrality. Moreover, *nudge, Halpern*, and *Behavioural Insights Team* are the only primary keywords to show greater negative than positive polarity.

What seems to associate with the observed non-divisiness? We are particularly interested in capturing patterns of neutrality as many may deem this to be the category of sentiment best suited to scientific discussion. Here we make three additional observations: (1) keywords *Michie* and *SPI-B* (emerging mid-lockdown) showed increasing presence over time but managed to maintain neutrality. Notably, *Michie* also attracted a small but sustained quantity of positivity over the full period; (2) *psychology* (with a stable and lowered presence in the media) shows to maintain a neutral presence over time and (3) *behaviour change* seems to be largely absent from the narrative, although we see a small surge at the point of lockdown (0; one week after the first surge), possibly aligned with an expected moment in time where many needed to change their behaviour. See Supplementary Material 4 for more detail.

**Figure 4:**
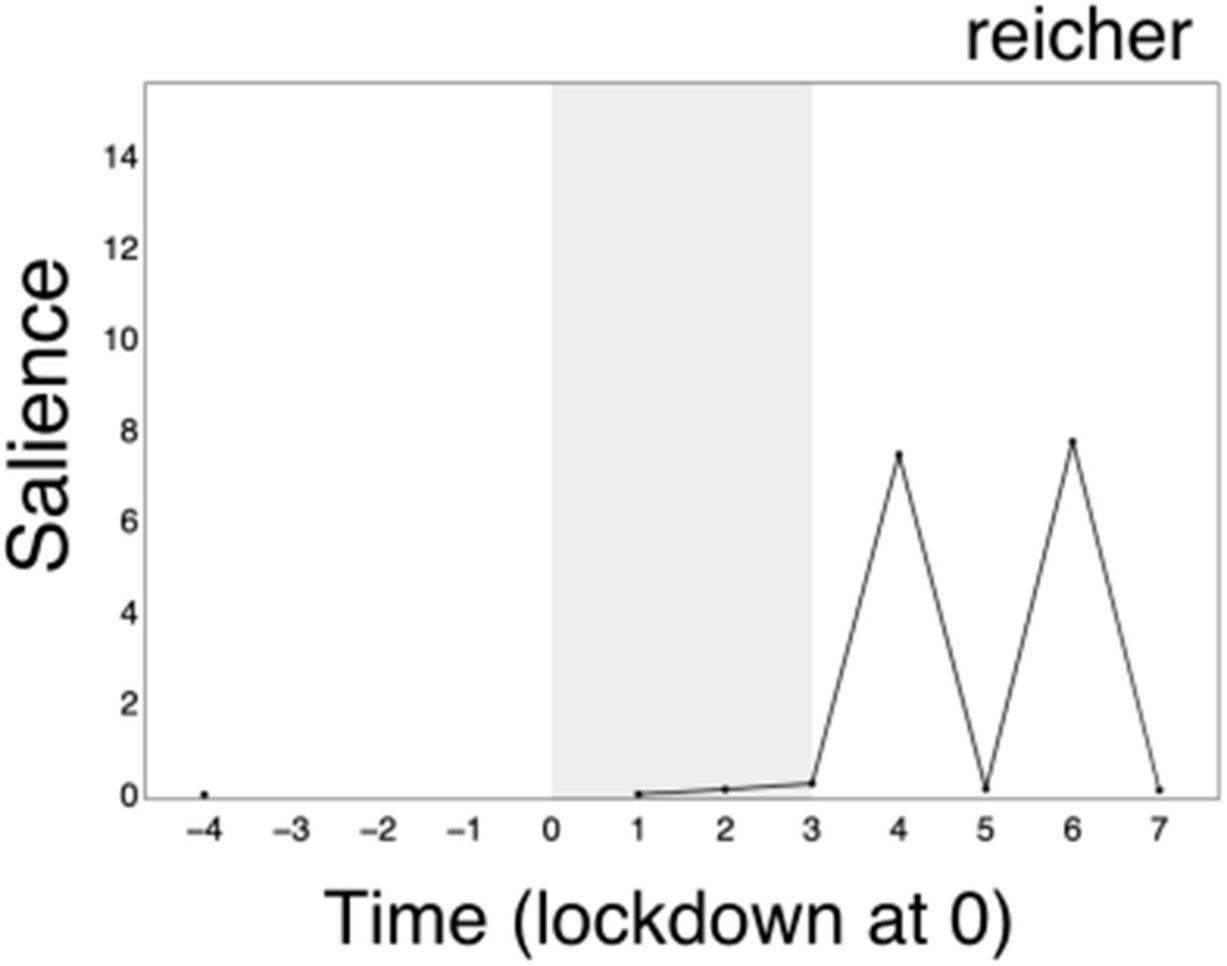
Qualitative inference identified Stephen Reicher as an additional actor. Reicher emerged on the topic of *behavioural science* toward the latter part of the 24-week time period, corresponding with a surge in salience (+6).

Finally, we note that our primary keywords do not provide insight into the second surge in divisiveness in *behavioural science* (aside from increased salience without sentimental fluctuation for *Michie* [+6] over this period), which lead to a qualitative inspection of the category of unnamed actors and resulted in identification of an additional key actor: Prof. Stephen Reicher (Supplementary Keyword: *Reicher*; see Figure 4). Further attention was paid to this in the qualitative analysis (Study 3).

#### Sentiment Toward Keywords in Context of Public Policy Application

**Figure 5:**
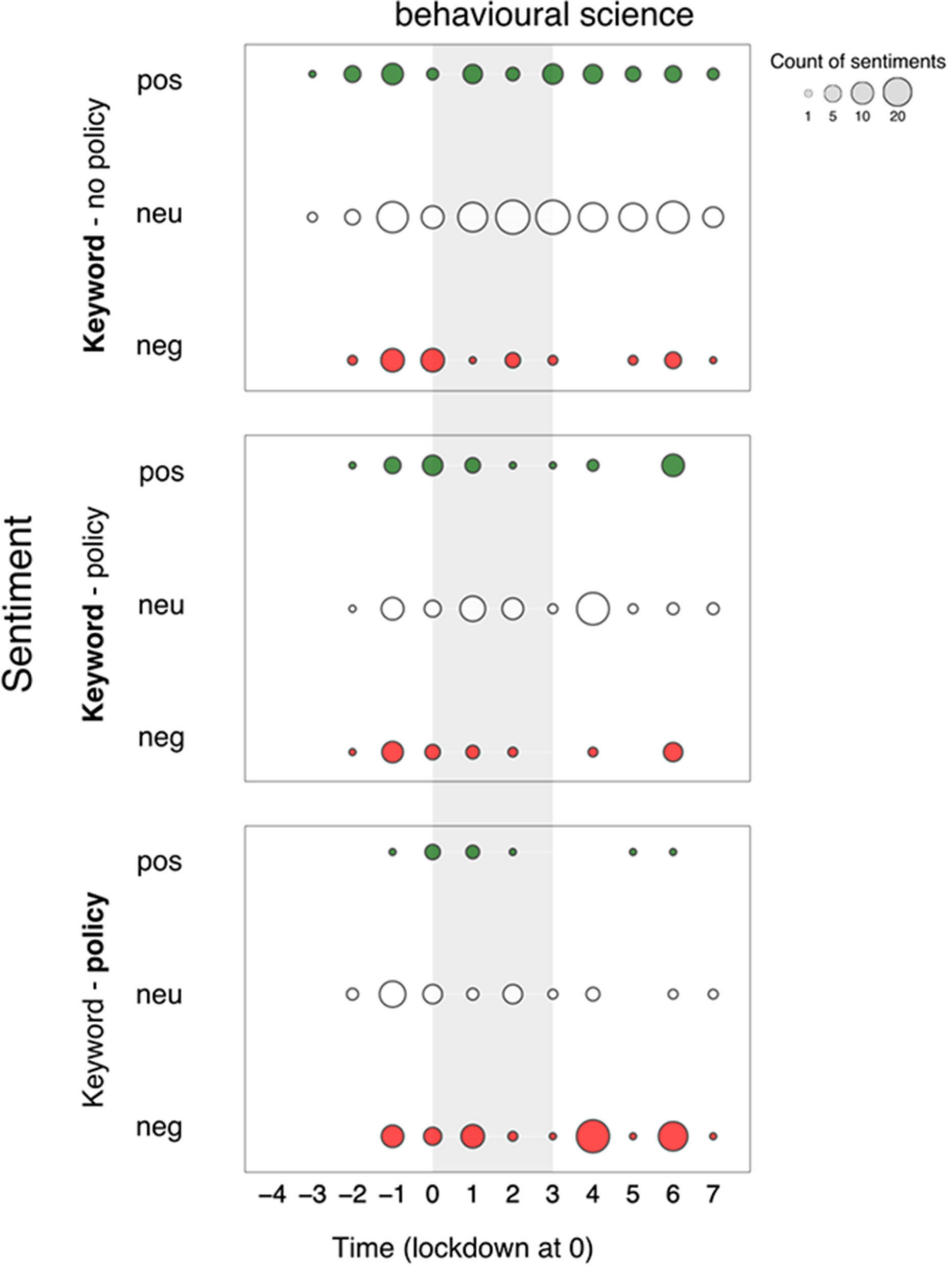
Sentiment toward “*Behavioural Science*” separated by sentences that do not (top) and do refer to national policy application (middle), and sentiment toward policy contexts of keywords (bottom) over the 12 two-week time period surrounding the first British national lockdown of 2020 from newspaper articles. Sentiments are represented in counts of positive (+1 or +2), neutral (0), and negative (−2 or −1) bubbles over time, in green, white and red respectively. The area of the bubbles is proportional to the count of sentiments toward the keyword. Reference category in bold.

We complement our understanding of sentiment expressed toward keywords by separating sentiments by those opinion contexts that refer to the application of *behavioural science* in public policy and those that do not. We display sentiments in three panels (see Figure 5): keyword sentiment when policy *was not* mentioned (top), keyword sentiment when policy *was* mentioned (middle), and sentiment toward policy application in those same opinion contexts (bottom; see data in Supplementary Materials 7, 8).

For *behavioural science*, we observe similar oscillations over time in all three panels, with two noteworthy differences between panels. First, we note higher neutrality and lower negativity toward *behavioural science* in opinion contexts which *did not* mention policy application (62% of neutral and 15% of negative sentiments overall) compared to those which *did* (52% of neutral and 22% of negative sentiments overall). In both contexts, the proportion of neutral sentiments toward *behavioural science* increased in the lockdown period (from 47 to 65% in contexts that *did not* mention policy and from 40 to 54% in contexts that *did*) and remained the highest post-lockdown. When we compare the sentiments toward *behavioural science* and its related *policy actors* in contexts in which both were mentioned (middle, bottom): we observe a much higher (57% overall) proportion of negative sentiments toward the policy actors, increasing across the three time windows (37% pre-lockdown, 42% during lockdown, 82% post-lockdown), paired with a decreasing proportion of neutral sentiments (60–40–15% post-lockdown). In opposition, the proportion of negative sentiment toward *behavioural science* shows a decreasing trend (from 37% to 19% to 18%). This suggests a transference of negative sentiment from the science of behaviour to the actors who are linked to it in this high-stake policy context over time. In other words, we do not only see a greater proportion of negativity toward *behavioural science* when mentioned in a policy context than when it is not, but we also see that the majority of this negativity is expressed toward the policy actors, and not *behavioural science* itself.

**Figure 6:**
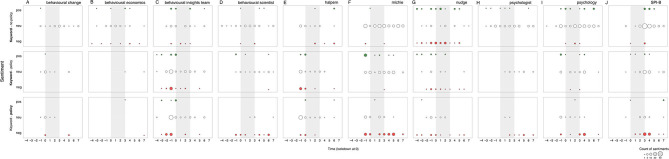
Sentiment toward the 10 primary keywords separated by sentences that do (top row) and do not refer to public policy application (middle row), and sentiment toward policy actors of keywords (bottom row) over a 12 two-week time period surrounding the first British national lockdown of 2020 in newspaper articles. Sentiments are represented in counts of positive (+1 or +2), neutral (0), and negative (−2 or −1) bubbles over time, in green, white and red respectively. The area of the bubbles is proportional to the count of sentiments toward the keyword. Reference category in bold. Column **(A)** behaviour change (concept), **(B)** behavioural economics (discipline), behavioural economics (discipline), **(C)** behavioural insights team (named actor), **(D)** behavioural scientist (unnamed actor), **(E)** halpern (named actor), **(F)** michie (named actor), **(G)** nudge (concept), **(H)** psychologist (unnamed actor), **(I)** psychology (discipline), **(J)** SPI-B (names actor).

What may result in the transference of negativity from *behavioural science* to the policy makers who use it? For sentiments expressed toward keywords in sentences that *do not* refer to policy application (Figure 6, top row) we recount two observations. First, for negative sentiment expressed toward *behavioural science not* in reference to policy, the picture is rather simple: prominent negativity is only observed around the concept of *nudge* (46% negative sentiments overall). This divisive, negative leaning pattern shows a small but consistent presence over the 24-week period, with a negative flare in the lead up to and throughout lockdown (echoed in articles which *do* mention public policy). Second, most keywords were more likely to appear in contexts that *do not* mention policy application (range 60–94% of their occurrences). The exceptions (unsurprisingly) *Behavioural Insights Team* and *Halpern*, which appeared in relation to policy actors in 69 and 63% of their occurrences respectively. In opinion contexts where policy was *not* mentioned, all keywords (aside from *nudge)* were discussed in neutral opinion contexts most often.

For opinion contexts that *do* mention policy application (Figure 6, middle row) and the sentiment *toward policy* (bottom row), we see a transference of negativity when the keyword is mentioned alongside *policy actors* for 9 out of the 10 keywords (just as *behavioural science*). We also observe two patterns: mentions of the common named actors *Behavioural Insights Team, Halpern*, and concept *nudge* share approximately equal numbers of negativity *with* the paired policy actors, suggesting a level of coupling pre-lockdown (−2, −1). This whilst discussion of actor *Michie* seemed to avoid negativity nearly entirely at cost to their policy co-mentions, suggesting a level of contrasting pre- (−2, −1), and mid- to end- lockdown (2–5). The latter pattern is echoed over the same time periods by a small but noticeable number of unnamed actors (*behavioural scientist* and *psychologist*), suggesting that a group of scientists may be “speaking out” against *behavioural science* application in policy.

The contrasting narrative offers insight into the drivers of a second surge in *behavioural science* divisiveness (+6). We observe that *psychologist, psychology*, and *SPI-B* collectively maintain neutrality, but share in negative sentiment expressed toward the co-mentioned policy application (bottom) in the post-lockdown period.

#### Co-occurrence Network Analysis

**Figure 7:**
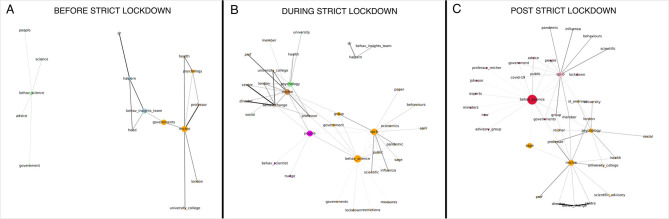
Networks of keyword co-occurrence across the three time periods: pre-lockdown **(A)**, during lockdown **(B)**, and post-strict lockdown **(C)**. Each node represents a keyword. Edgelines represents the strength of the co-occurrence (Dice coefficient) between two keywords. The node size represents the keyword's weighted betweenness centrality (a small constant has been added to all nodes so that all could be visible in the graph). Nodes are colored according to their community, as detected by the Louvain modularity algorithm.

Finally, we look at which keywords, actors, and concepts frequently co-occur in opinion-context with one another, complemented by five metrics: network density, network clustering, node degree centrality, node betweenness centrality and detected communities (“thematic clusters”) (Figure 7; Table 1; Supplementary Material 6).

**Table 1:** Structural network statistics on the opinion-context co-occurrence networks pre- during- and post-lockdown.

	**Pre-lockdown** **(27 Jan−22 Mar)**	**Lockdown** **(23 Mar−09 May)**	**Post-lockdown** **(10 May−10 July)**
Number of nodes	16	36	37
Network density	0.14	0.09	0.09
Network average clustering coefficient	0.18	0.11	0.08
Weighted degree centrality (descending rank, top 10 terms)	Michie Halpern (–) Behav._insights_team (–) Psychology Professor Head Health Behav_science Governments Dr	Michie Behav_change (+) SPI-B (+) Behav_science (+) Psychology People University_college Professor London Center	SPI-B Michie Behav_science Psychology Behav_change Director Center Member Professor Group
Weighted betweenness centrality (descending rank, top terms above 0.0)	Michie Governments Behav_insights_team (–) Halpern Psychology Behav_science	Behav_science (+) SPI-B (+) People Michie Psychology Group Government Halpern (–)	Behav_science Michie SPI-B SAGE Psychology
Louvain modularity (resolution parameter = 1.0)	0.53	0.48	0.44

*Movements of keywords into and out of the top 5 for degree centrality and betweenness centrality are marked with (+) and (–), respectively, with the first period as the reference category*.

For the network structure (see Table 1). Over the hard lockdown period we observe larger networks associated with lower network density (pre = 0.14; during = 0.09; post = 0.09) and lower clustering coefficients (pre = 0.18; during = 0.11 post = 0.08) than over the other two time periods. This suggests that the narrative structure around keywords was less cohesive and more diverse in opinion contexts in the two later periods. When considering degree centrality, *Michie* is the most central keyword across all three time periods; *Halpern* and *behavioural Insights Team* are central pre-lockdown but move down the structural importance ranks after that. Showing an opposite trend, *SPI-B* and *behaviour change* surge to relevance from the start of lockdown onwards. When it comes to connecting clusters of words (betweenness centrality), named actors michie and behavioural Insights Team play a central role pre-lockdown, but *behavioural science* and SPI-B serve to connect during- and post- lockdown. Notably, psychology is the only other discipline with structural importance across all three networks.

Interestingly, the non-keywords government(s) and people play an important connecting role pre- and during lockdown, probably reflecting the public policy context in which the keywords are discussed in those periods.

Trends in “thematic clusters” (see Figure 7). Pre-lockdown sees three strong associations: keyword *Halpern* coupled with *Behavioural Insights Team, Michie* coupled with *psychology*, and *behavioural science* coupled with “government,” “people,” and “advice.” Keyword behavioural Insights Team is connected through *Michie via* “governments” presumably highlighting the influential roles of the two in public policy. Interestingly, *behavioural science* forms a separate thematic cluster, suggesting *behavioural science* was discussed independently of its main national actors over these time periods.

During-lockdown, we observe that the narrative around *behavioural Insights Team* disconnects from that of other keywords in the opinion contexts. Keyword *michie* remains relevant and now includes relation to *behaviour change*, and now connects to keywords *behavioural science* and *SPI-B* via “government.” These last two keywords form a unique cluster and are discussed in wider relation to policy responses central to pandemic management (e.g., “measures,” “lockdown,” “behaviours,” “public”). We note two interesting facts: *psychology* forms a separate thematic cluster from *behavioural science*; “people” surges to a prominent connector role and forms a cluster with keyword *nudge* and *behavioural scientist*, presumably highlighting newspaper's practice to introduce behavioural interventions as relevant to individual and group behaviours (i.e., nudging people).

Post-lockdown, we make three novel observations: *michie, SPI-B* and *behavioural science* now solidify as the centers of three highly interconnected clusters. We note the emergence of the new actor “(professor) reicher,” and psychology, “SAGE,” and “governments” working as connectors between the *michie-behaviour change's* and the *behavioural science's* clusters.

### Discussion

Study 1 maps the discourse of *behavioural science* around the UK lockdown decision through trends in keywords and sentiment toward them. We find that increased salience can be linked to divisiveness in sentiment, associated with a cluster between *Behavioural Insights Team* and *Halpern* (and later also *nudge)* coupled with policy application of *behavioural science* in the first (pre-lockdown) wave. This coupling may be a reflection of the embedded relationship between application of *behavioural science* in governance and the work of BIT. Whilst their collaboration has allowed advancement of applying the science of *behavioural science* in many public policy areas, one possibility is that the tight relationship was deemed less acceptable under the high-stake policy conditions which were faced.

*Nudge*, independently of whether it was coupled with policy application of *behavioural science*, also seems to stir divisiveness. This may be a sticking point for trust and credibility in the public eye which seems, to a degree, to be generalizable (Hagman et al., 2015; Treger, 2020), yet simultaneously does not seem to be of any structural importance to the pre- during- or post-lockdown narrative. Other than that, the application of *behavioural science* in high-stake policy incurred relatively high negativity in media discourse, but this did not reflect necessarily on the science of behaviour, but rather in reference to its policy counterpart. In relation, two other clusters of associations seem to have been impactful. Key actors such as *Michie, SPI-B*, and the unnamed *psychologist* and *behavioural scientist* contrasted positively to *behavioural science* application in national-level governance. This suggests that one of the factors to have played into the trust and credibility of *behavioural science* (and its readiness for policy application) emanated from *behavioural science* actors themselves speaking out against its potential misuse as a policy tool under the high-stakes circumstances, and this seemed of particular influence a few weeks after the lockdown started to ease.

Finally we note that *behavioural science* is captured in a separable narrative from the frequent actors (SPI-B, Michie, BIT, Halpern). Over the course of the three time periods behavioural science starts to increasingly function to bridge these actors. This seems indicative of its “catch all” terminology: capturing the versatile and heterogeneous perspectives it represents. In addition, *behavioural science* offers the important bridge to national level policy applications (with terms such as “lockdown,” “measures,” “restrictions,” and “advisory_group”).

## Study 2 Social Media Discourse Analysis

### Introduction

In Study 1, we looked at patterns of salience and sentiment toward *behavioural science* in newspaper articles over the 24-week period surrounding the first UK lockdown of 2020. This analysis does not tell us how the public responded to these articles. To identify whether such stories gained traction on social media, we next identified a set of publicly available Twitter data to track the keywords identified in Study 1. Twitter is amongst the most frequently used social media to investigate public's perceptions across a range of topics (Bibo et al., 2014; Arribas-Bel et al., 2015; Bian et al., 2016; Ordun et al., 2020; Sharma et al., 2020). Twitter is popular for capturing public perceptions with over 330 million registered global users who dynamically generate over 500 million messages (also called “tweets”) per day (Chae, 2015; Mention, 2018). We opted for this (as opposed to another) social media platform for: (1) Twitter's informal, colloquially generated and unconstrained opinion data (Fried et al., 2014; Moe and Schweidel, 2017), (2) Twitter's ability to attract individuals focused on information sharing and seeking (Hughes et al., 2012). We reasoned that mapping the salience and sentiments of the identified *behavioural science* concepts and actors from Study 1 over the same time period in this dataset, would allow us to identify the nature and extent of concordance of public opinion in line with that expressed in print media.

### Materials and Methods

#### Materials

We used the Coronavirus Tweet Ids Version 7 dataset (Kerchner and Wrubel, 2020) from TweetSets, the archive of Twitter datasets for research and archiving managed by George Washington University (Littman, 2008). The Coronavirus dataset contains the tweet IDs of 239,861,658 tweets related to COVID-19, collected between March 3, 2020 and June 9, 2020 from the Twitter API using the tags “coronavirus,” “COVID-19,” “epidemiology,” “pandemic.” This dataset was selected as it was the open-source dataset of tweets that most closely reflected the timeframe and context of the news articles retrieved for Study 1.

TweetsSets allows querying the database of tweets based on keywords, hashtags, and other parameters, even if the user then only receives the tweetIDs. Thus, similar to Study 1, we developed a query to identify tweets relevant to the discussion of *behavioural science* and its application to public policy during the COVID-19 pandemic (see Supplementary Material 2B for details of the stages and final query). Our query resulted in a dataset of 13,664 tweet IDs, corresponding to around 0.006% of the initial dataset. We then used Hydrator (Documenting the Now, 2020) to hydrate these tweets IDs, i.e., retrieve the text of the tweets and associated metadata from the Twitter API, which resulted in 12,161 tweets.

**Figure 8:**
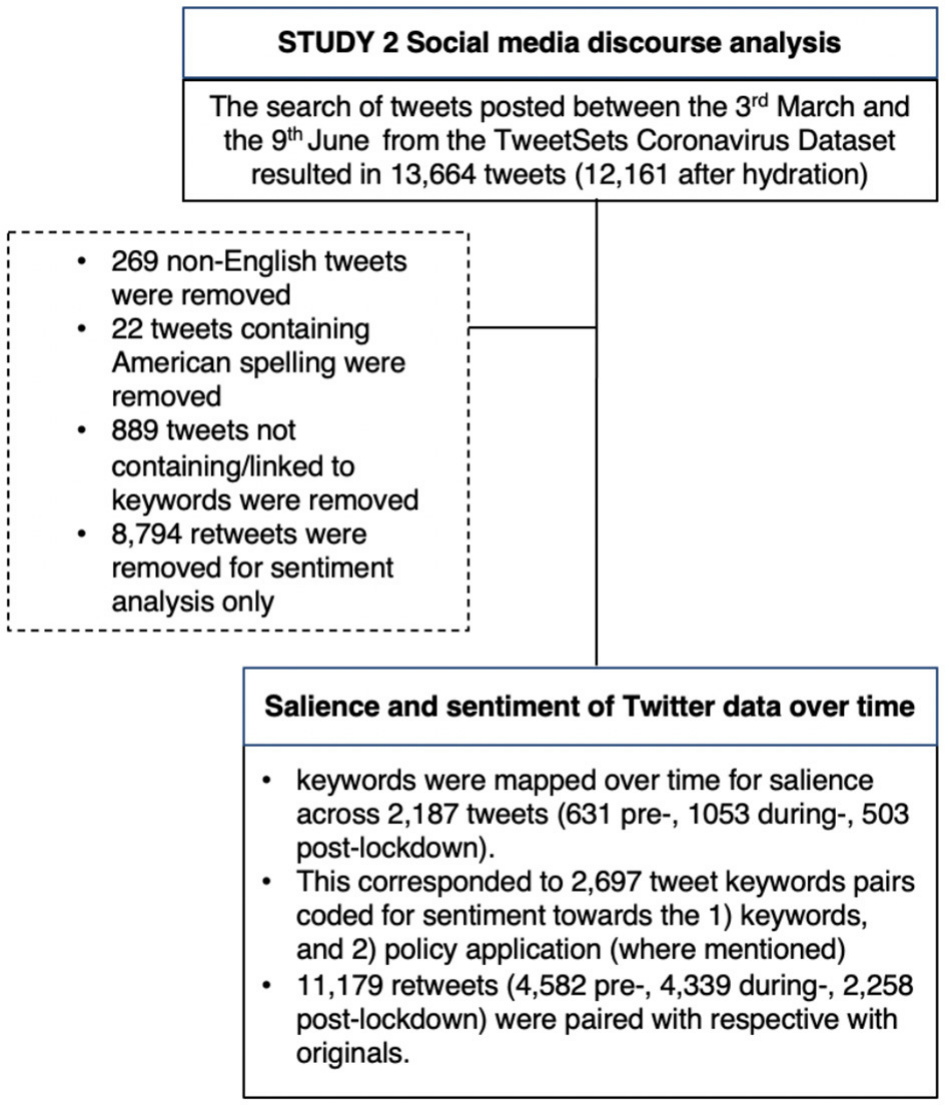
Flowchart of data selection and cleaning process taken for Study 2.

We removed retweets (8,794) using regular expressions to focus the analysis on original tweets as retweets can inflate the number of unique messages for the sentiment analysis. Two hundred and sixty-nine tweets that were not in English were also excluded. Of the remaining tweets, 462 contained no behavioural science keyword (the keyword was mentioned in another tweet linked from within the tweet) and 427 other tweets only contained coronavirus-related search queries but no behavioural science keywords: they were all excluded from analysis. Finally, we also removed 22 tweets that displayed American spelling of behavioural science keywords (e.g., *behavioural science*). We analyzed the remaining 2,187 tweets (631 pre-, 1,053 during-, 503 post-lockdown), corresponding to 2,697 keyword-tweet pairs, and their 11,179 pure retweets (sum of their “retweet_counts;” 4,582 pre-, 4,339 during-, 2,258 post-lockdown). See Figure 8 for a step-by-step.

#### Keyword Processing

To allow for comparison, we focused our analyses on the 11 primary keywords retained for analysis in Study 1 (see Supplementary Materials 3, 5B for details).

#### Analyses

##### Salience

We used document frequency (the proportions of tweets within a 2-week period in which the primary keyword occurred) as our measure of salience for the Twitter data. This differs from Study 1 (where we used document frequency multiplied by normalized term frequency): on Twitter, keywords tend to appear once per tweet (of 2,697 keyword occurrences, only 122 [4.5%] contained the same keyword more than once), and the number of total words per tweets is limited (max. 280 characters) and highly consistent (median = 32 words; IQR = 16 words). To assess salience over time we calculated two metrics: (i) *Salience (original tweets only)*: the proportion of total tweets in a given fortnight in which the keyword occurred. (ii) *Salience (accounting for retweets)*: the proportion of total tweets and retweets in a given fortnight in which the keyword occurred.

##### Sentiment

We coded sentiment toward keywords and public policy in original tweets as per Study 1, but report two sentiment measures: (i) *Sentiment (original tweets only)*: the count of positive/neutral/negative sentiments toward a keyword per 2-week period; and to account for the reach of sentiment expressed we also calculate (ii) *Sentiment (accounting for retweets)* by multiplying each sentiment by the number of times the tweet that contained it was retweeted^3^.

### Results

#### Salience and Sentiment of Keywords Over Time

**Figure 9:**
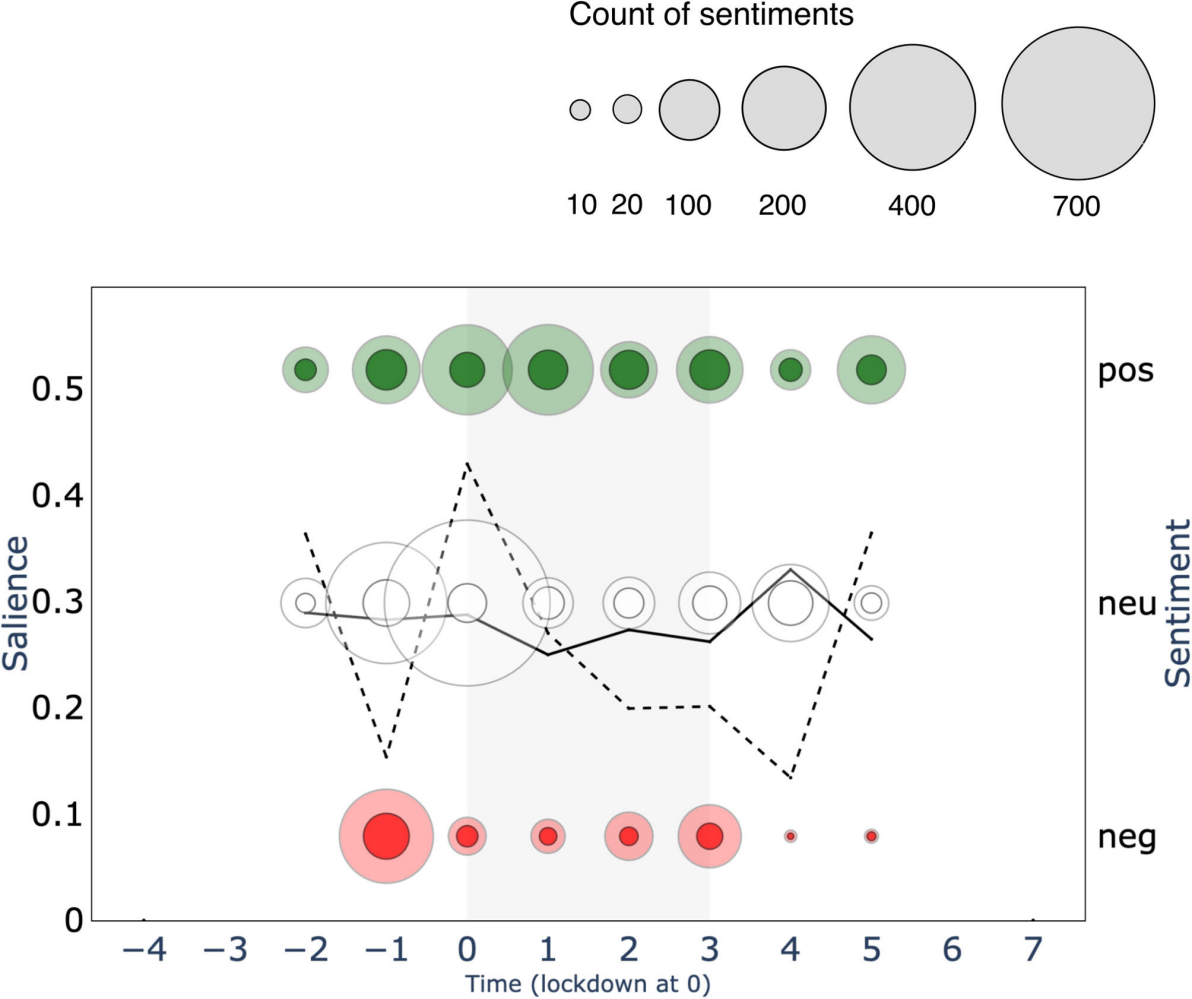
Salience and sentiment of “*Behavioural Science*” over the 8 two-week time-period surrounding the first British national lockdown of 2020 (gray area) in Twitter data. Salience is calculated as the proportion of tweets in that 2-week period that mention the keyword. Bold line represents salience in original tweets only; Dotted line represents salience accounting for retweets also. Sentiments are represented in counts of positive (+1 or +2), neutral (0), and negative (−2 or −1) bubbles over time, in green, white and red respectively. The area of the bubbles is proportional to the count of sentiments. Full-color bubbles represent sentiments in original tweets only; shaded-color bubbles represent sentiments accounting for retweets.

With regard to salience of behavioural science, Figure 9 shows a notably stable trend in original tweets over time, but when we include retweets (dotted line), we observe a pattern largely similar to that of newspaper articles: two surges, one during the fortnight at the start of lockdown (0) and one post-lockdown (5). See also Supplementary Material 9.

Regarding sentiments, original tweets that mention *behavioural science* attract similar levels of divisiveness in the two weeks prior to lockdown (−1; 37% neutral; 27% positive; 36% negative) and at the end of lockdown (3; 34% neutral, 46% positive, and 20% negative) as compared to our set of newspaper articles. Negative sentiments also similarly reduce as the lockdown eases. We do note higher levels of positive and neutral sentiments, which remain relatively constant throughout the entire period, with a noticeable surge in neutral retweets just prior to the start of lockdown (−1 = 52% of all sentiments; 0 = 74% of all sentiments).

**Figure 10:**
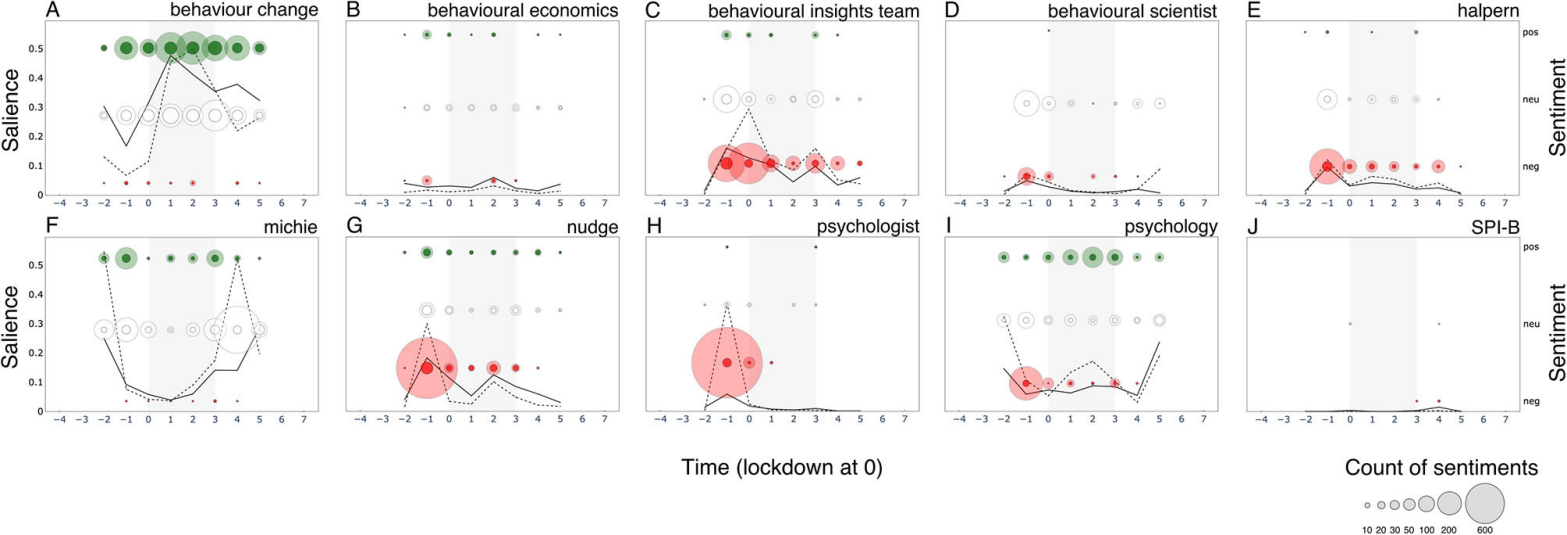
Twitter - Salience of and sentiment toward primary keywords over 8 two-week time-period surrounding the first British national lockdown of 2020 (in gray): **(A)** behaviour change (concept), **(B)** behavioural economics, **(C)** behavioural insights team (named actor), **(D)** behavioural scientist (unnamed actor), **(E)** halpern (named actor), **(F)** michie (named actor), **(G)** nudge (concept), **(H)** psychologist (unnamed actor), **(I)** psychology (discipline), **(J)** SPI-B (names actor). Salience is calculated as the proportion of tweets in that 2-week period that mention the keyword. Bold line represents salience in original tweets; dotted line represents salience including retweets. The area of the bubbles is proportional to the count of sentiments (red = −2, −1; white = 0; green = +1, +2) toward the keyword. Full bubbles represent sentiments in original tweets only; shaded bubbles represent sentiments accounting for retweets.

Comparing coverage of keywords on twitter (Figure 10, Supplementary Material 9) with that in newspapers (Figure 3), we see that *Michie* and *behaviour change* even more strikingly attract neutral and positive sentiment than in print media, and that *behavioural economics* is similarly absent from the conversation. We also see the same negativity toward *Halpern, Behavioural Insights Team* & *Nudge* just before lockdown. Unlike in media discourse, pre-lockdown negativity is also present for *psychology, psychologist* and *behavioural scientist*, suggesting that, in the Twitter public discourse, negativity is extended to the discipline and professions of these actors. And unlike in the newspapers, *SPI-B* is nearly entirely absent from Twitter chatter.

Comparing trends in tweets with retweets offers three interesting insights. First, most retweets are of negative sentiment. *Nudge* and *psychologist* see a dramatic surge in retweet (but not tweet) salience just prior to lockdown (−1), corresponding a burst of negative sentiment. *Behavioural Insights Team* sees a similar pattern, but delayed by two weeks (0). All three keywords see a decrease in tweet/retweet salience and negative sentiment thereafter. Second, *Michie* sees a surge in tweet and retweet salience before (−2) and after lockdown (4 and 5), both retaining high levels of positive and neutral sentiment. Third, *behaviour change* surges (starting from period 0) and remains high in salience throughout the period, in association with positive or neutral sentiments. For these two keywords (unlike all others), positive sentiments are retweeted most.

#### Sentiment Toward Keywords in Context of Public Policy Application

**Figure 11:**
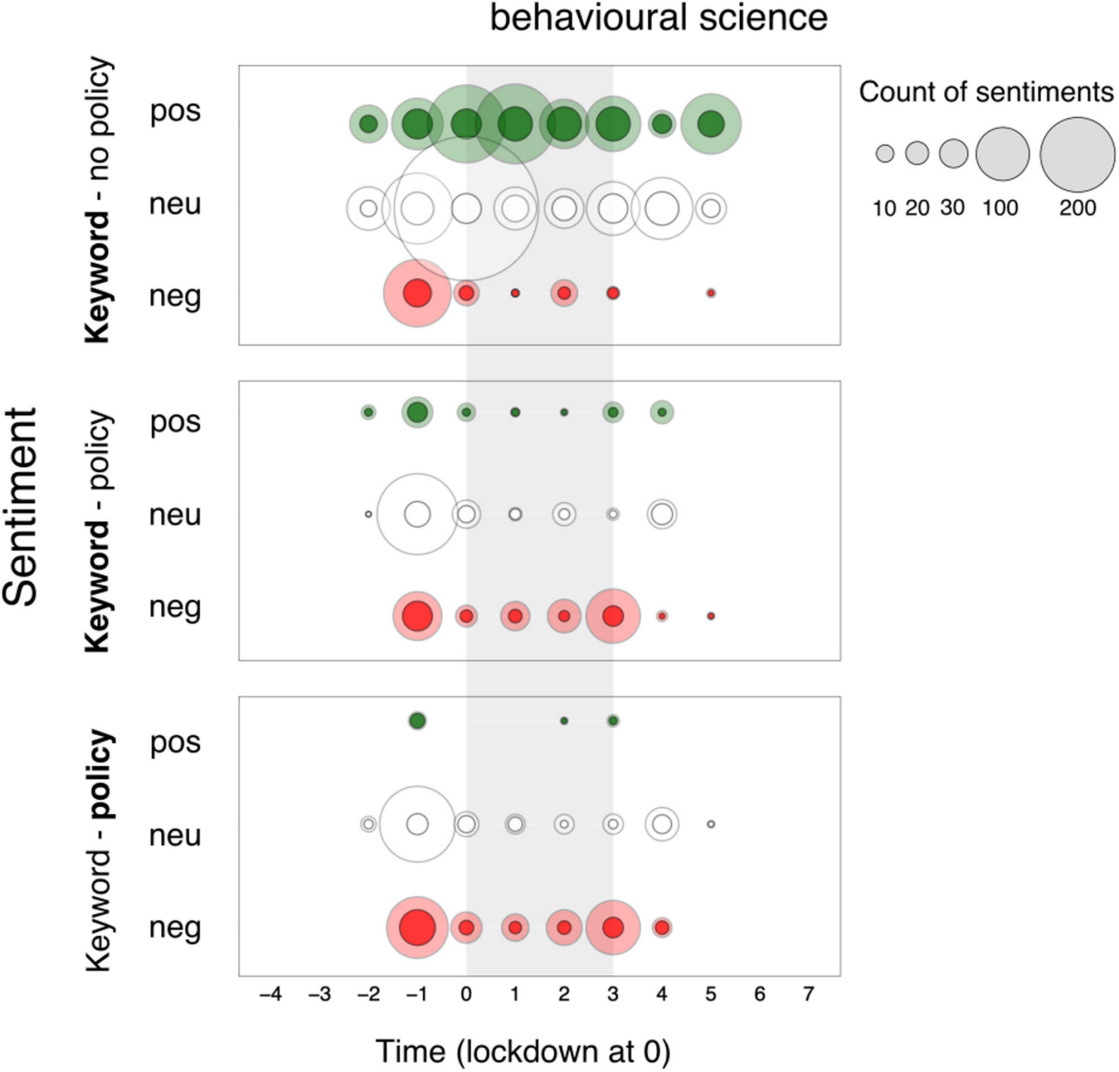
Twitter articles - Sentiment toward “*Behavioural Science*” separated by sentences that do (top) and do not refer to policy application (middle), and sentiment toward policy contexts of keywords (bottom) over the 8 two-week time period surrounding the first British national lockdown of 2020. Sentiments are represented in counts of positive (+1 or +2), neutral (0), and negative (−2 or −1) bubbles over time, in green, white and red respectively. The area of the bubbles is proportional to the count of sentiments toward the keyword. Full-color bubbles represent sentiments in original tweets only; shaded-color bubbles represent sentiments accounting for retweets.

How does mention of policy context affect perceptions of *behavioural science*? In Figure 11, we display sentiments in three panels: keyword sentiment when policy was not mentioned (top), keyword sentiment when policy was mentioned (middle), and sentiment toward policy application in those same opinion contexts (Figure 11 bottom; see data in Supplementary Material 10). Two patterns stand out as distinctive from those in print media. First, a larger majority of positive and neutral sentiments toward *behavioural science* are expressed when this is *not* mentioned alongside policy applications (top panel), with a burst of retweets of neutral sentiments (−1, 0). Second, the patterns of sentiments expressed toward behavioural science when policy application *is* mentioned (middle panel), and the sentiments expressed toward policy application itself (bottom panel) are closely matched. Just as in print media, we see a prevalence of negative sentiments throughout the period under consideration, with a burst in negativity just before (−1) and at the end of lockdown (3).

**Figure 12:**
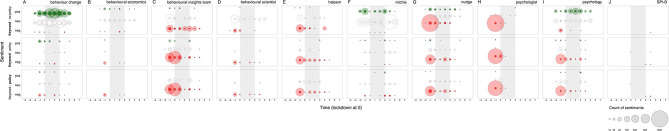
Twitter articles—sentiment toward the 10 primary keywords separated by sentences that do (top) and do not refer to policy application (middle), and sentiment toward policy contexts of keywords (bottom) over the 8 two-week time period surrounding the first British national lockdown of 2020. Sentiments are represented in counts of positive (+1 or +2), neutral (0), and negative (−2 or −1) bubbles over time, in green, white and red respectively. The area of the bubbles is proportional to the count of sentiments toward the keyword. Full-color bubbles represent sentiments in original tweets only; shaded-color bubbles represent sentiments accounting for retweets. **(A)** behaviour change (concept), **(B)** behavioural economics (discipline), **(C)** behavioural insights team (named actor), **(D)** behavioural scientist (unnamed actor), **(E)** halpern (named actor), **(F)** michie (named actor), **(G)** nudge (concept), **(H)** psychologist (unnamed actor), **(I)** psychology (discipline), **(J)** SPI-B (names actor).

Separating out the sentiments by mention of policy application for the other primary keywords on twitter (Figure 12; see Supplementary Materials 10, 11) allows us to capture three complementary results distinctive from the pattern observed in newspaper articles (Figure 4). First, the striking majority of positive sentiments expressed toward behavioural science keywords are *not* in reference to policy in association with 3 primary keywords: *behaviour change* (during lockdown)*, michie* (pre- and post- lockdown), and *psychology* (during lockdown). Second, keywords which attracted negative sentiment (*Behavioural Insights Team, Nudge, Halpern*) toward policy-referenced tweets (middle row), attracted similar (not more) negativity in non-policy referenced tweets (top row). Third, negativity expressed toward keywords (middle row) and their policy application (bottom row) when mentioned together, is strongly coupled throughout the set of tweets.

### Discussion

As was the case for Study 1, Study 2 shows that the rapid emergence of negative sentiment toward the embeddedness of *behavioural science* in the initial phase of COVID-19 restrictions is found within public discourse as well.

Twitter data also held more extreme sentiments, and increasingly coupled sentiment between *behavioural science* and its policy actors. This may in part be due to Twitter's succinct communication format (difficulty to express contrasting opinions with limited characters) but may also reflect a coupling in actual public opinion. We see some evidence for this: some tweets *did* express contrasting views (e.g., *Michie, nudge, Behavioural Insights Team*), but do not seem to hold the same retweet value. In fact, we see that tweets expressing negative sentiment toward *behavioural science and* its policy counterpart gained most traction overall. Second, we see that negativity is linked to a clustering of *Behavioural Insights Team*, and *Halpern* in the pre-lockdown period (just as in print media), but on twitter the negative sentiment also extends to their professions (*behavioural scientist; psychologist*).

Further to this, it is not possible to ascertain whether negative sentiment surrounding the *behavioural science* linked to government policy reflects negative sentiment toward the government transferring onto the involvement of *behavioural science*, or more general antipathy toward the type of *behavioural science* approaches employed by the government. It is clear that *behavioural science* and behavioural change approaches seen as independent of or even in opposition to government policy received a greater deal of both social media attention and positive sentiment, particularly in association with *behaviour change* or *psychology*, something highly consistent with findings from Study 1.

## Study 3 Thematic Analysis of Newspaper Articles

### Introduction

Studies 1 and 2 provide us with patterns of salience and sentiment toward the *behavioural science* in terms of its perceived “place” in high-stake public policy from journalistic and social media. To better contextualize these insights and examine how levers (or barriers) of trust and credibility toward *behavioural science* in contexts of high-stake policy making are constructed in the media, Study 3 utilizes a qualitative design, analyzing a subset of articles from Study 1 and with reference to data from Study 2.

### Materials and Methods

Articles were selected to include all instances of extreme sentiments (+2 or −2). This included a sample of (1) extreme sentiment toward the *behavioural science* keywords *and* public policy keywords, (2) extreme sentiment toward the *behavioural science* keywords, with neutrality toward public policy keywords, (3) neutrality toward the *behavioural science* keywords and extreme sentiment toward public policy keywords (see Table 2). The total sample of articles (*N* = 111) was analyzed using NVivo 12.

**Table 2:** Distribution of selected articles across the three time periods (pre-, during-, and post- lockdown) and sentiments toward *behavioural science* and public policy.

	**Sentiment toward behavioural science keyword**	**Sentiment toward policy application**	**Total number of articles (overlap)**
Congruent positive	+2	+2	8 (5)
Incongruent neutral	+2	0	2 (4)
Contrast	+2	−2	7 (7)
Incongruent neutral	0	+2	1 (0)
Incongruent neutral	0	−2	63 (7)
Contrast	−2	+2	0
Incongruent neutral	−2	0	8 (0)
Congruent negative	−2	−2	22 (9)

*Some articles appear in more than 1 category (overlap in brackets)*.

The method of analysis was deductive following standard procedures for codebook approaches to thematic analysis (Ritchie and Spencer, 1994) with an emphasis on contextualizing the findings from Study 1. In particular, given the differential coverage of actors, the rise and fall in emphasis on *behavioural science* and the patterns found in relation to sentiment toward public policy, the qualitative analysis focused on examining three questions which emerged from Study 1:

1. *How is the UK's approach to the pandemic framed as compared to that of other national approaches, with regards to trust and credibility?*
2. *How are the behavioural science discussed and compared to other sciences, with regards to trust and credibility in handling the pandemic?*
3. *How is Behavioural Science introduced in the articles, under which circumstances and how does this framing emphasize trust and credibility in the science?*

Specifically, the analysis entailed coding for actors (including scientific actors, government actors and international organizations such as WHO), sentences describing or discussing *Behavioural Science*, and sentences mentioning different countries approaches to COVID-19.

### Results

#### *Behavioural Science* as Part of a National Response Policy

Three themes thought to affect credibility of, and trust in *behavioural science* were identified in relation to the UK's national approach to the pandemic frame: (1) divergence from that of other countries and global policy recommendation; (2) perceived incongruence between the approach and adherence of most senior members of parliament; (3) expressed concern by scientific experts and government advisors.

##### Perception of UK Policy Response as Divergent

Most frequently the UK COVID-19 response is regarded through drawing on a comparative lens, questioning why it deviates so significantly from that of other countries;

“Over the next fortnight, as Italy moved to impose a lockdown, France and Spain began to do the same, and Germany embarked on physical distancing measures coupled with Europe's most extensive testing and contact tracing operation, Britain did comparatively little.” (Conn et al., 2020).

In addition, there was frequent mention of how the UK's approach deviated from the one promoted by the World Health Organization (WHO), which was perceived as a credible source to trust;

“The key principles from WHO are intensive surveillance. […] Yet the UK government is no longer testing anyone outside of hospitals, he warned. Prof. Costello added: “For me and the WHO people I have spoken to, this is absolutely the wrong policy. It would mean it just let's rip.” (Mullin, 2020).

##### Perception of Internal Incongruence

The lack of trust toward the national policy is amplified by frequent reports of incongruence between nationally imposed regulations and the adherence to these by parliamentary personnel who were part of developing the regulations (e.g., discussion of Dominic Cummings' action as warranted or disregard for regulations);

“Professor Susan Michie, director of the Center for Behaviour Change at University College London, said: “Whilst the PM was telling people to stay at home and keep at least two meters apart from each other, the House of Commons was open for business and face-to-face parliamentary activities were carrying on.” Given the transmission routes of touching contaminated surfaces and breathing in virus-laden droplets, it should not come as a surprise to hear that the PM and Health Secretary have tested positive for coronavirus. “There are many reasons why those in leadership positions, including in Government, should practice what they preach.” (Kirby et al., 2020).

##### Concerns From “Allied” Scientific Advisors and Experts

When critiques like the above come from scientists named and identified as *government* advisors (e.g., as part of the Scientific Advisory Group for Emergencies), the lack of trust toward government is further elevated. We note that this explains in part the positive sentiment expressed toward Prof. Susan Michie in Study 1 and 2, where her positioning as a scientist who aligns herself with a critical public (often using Twitter to do so) functions to position her as a scientist working for the public good (as opposed to in association with government). This is echoed if we look more closely at the most salient tweets in Study 2, where a positive reference to Michie was the third most retweeted (over 600 times);

“Professor Susan Michie of University College London has praised Nicola Sturgeon and Scotland's approach to COVID-19. Another blow for #ColonialQuay and BritNats! #TheNine #COVID19.” (Indy Swim, 2020).

Negative perceptions of the UK policy response (in contrast to that of countries perceived to have successfully suppressed infection rates) are also reinforced by drawing on national and international scientific expert whom, as a collective, comment and critique its incoherence with a globally united response to the pandemic;

“Public health experts and hundreds of doctors and scientists at home and abroad are urging the UK government to change its strategy against coronavirus, amid fears it will mean the epidemic “lets rip” through the population. They say the UK is turning its back on strategies that have successfully brought down the numbers of infections and deaths in other countries.” (Boseley, 2020a).

The inclusion of scientific experts criticizing the COVID-19 response policy opens up assumptions around *which* scientists might support the national approach, as it is argued to be informed by scientific knowledge. Here we see the coupling of *behavioural science* and public policy emerge, and the negative sentiment spills into how the behavioural science are perceived;

“The government's strategy has at its heart predictions about human behaviour. […] Which analyses of human behaviour are government scientists relying on? And how comparable are they? Why is fatigue such a problem for new coronavirus measures, which we might expect would command the same kind of support as a war effort, when the state lives with this “fatigue” in the design of the laws and norms that permanently regulate our lives? We can't answer these questions, because the government's scientists aren't yet disclosing what studies and past evidence underpin their current approach. The government's tactic - one might even call it a nudge - is to appeal to the credentials of its advisers and behavioural scientists, and to trust the experts.” (Yates, 2020).

In conclusion, perceptions of uniqueness, lack of adherence to regulations by parliamentary members, and experts questioning the science informing the UK strategies lead to a media framing of the UK COVID-19 policy response as neither trustworthy nor credible. Behavioural science is initially introduced as what makes the UK response national approach unique and gets caught in the debate.

#### *Behavioural Science* Relative to Other Sciences

Next, we examined how *behavioural science* was discussed, in comparison to other scientific approaches, to see which framings did or did not align with public trust and perceived credibility. Here too, we note two themes surrounding trust and credibility: (1) mentions of achievement; (2) scientific experts expressing opposing views.

##### Mentions of Achievement

We identified which scientific experts were named and how articles positioned the expertise of their respective fields. Unsurprisingly (based on the query) *behavioural science* actors were mentioned most, followed by public health experts and epidemiologists. Scientific disciplines were often mentioned through academic titles, achievements, previous contributions to policy or other contexts of global threat. These introductions consistently lent credibility to the expertise of all scientists (behavioural and other);

“Anthony Costello, a UK pediatrician and former director of the World Health Organization (WHO)…” (Boseley, 2020a).

“…a leading behavioural scientist has said. Susan Michie, professor of health psychology at University College London…” (Fisher and Lay, 2020)

“. the British scientist leading one of the world's most advanced efforts has said. Sarah Gilbert, professor of vaccinology at Oxford University…” (Thomson et al., 2020).

##### Sciences in Opposition

Scientific experts were also found to express criticism toward other scientific disciplines. We thus examined for which disciplines this occurred and attempted to distill the impact on their credibility in the eyes of the public. While much criticism voiced by experts was leveraged at the national policy approach (as described above) instances of critique at other sciences were also found;

“In March some epidemiologists privately expressed frustration over behavioural scientists advising the government to lockdown later over fears people would tire of restrictions.” (Smyth, 2020a).

Such expressions of concern often associated with unnamed scientific actors (“immunologist;” “epidemiologists”) cast doubt on the validity of the contrasted science. In fact, both of the most frequently retweeted tweets identified in Study 2 negatively contrast *behavioural science* with epidemiologists (see second tweet in next section);

“The government's science advisor is a behavioural psychologist, not an epidemiologist. This is crowd management.” (Seymour, 2020).

Similarly, articles reveal drivers of credibility and trust in *behavioural science* in contrast to other sciences, emphasizing the need to consider behavioural implications of different policy options;

“David McAdams worries that the health scientists are using simplistic “*ad hoc* assumptions about behaviour” when complex nudges, such as “effective political leadership,” can have big impacts. Understanding motivations properly is vital. Rich people will lock down voluntarily, but poor people may prioritize work. Policies could be tweaked accordingly. […]. The government's slavish following of epidemiological advice has been a disaster, a lockdown soft enough to leave the UK with a tenth of the world's deaths but hard enough to wipe out up to a third of economic output.” (Aldrick, 2020).

We conclude that credibility is extended to characteristics that highlighted the expertise of a particular individual interviewed or quoted in the articles, but that the contrasting perspectives between disciplines, embodied by the voices of different experts criticizing one another, serve as a barrier to trust and credibility in media surrounding what is deemed suitable science to aid toward a health pandemic. The approach of contrasting is similarly but less frequently found in support of behavioural scientists.

#### Key Actors and Concepts of *Behavioural Science*

Lastly, we analyze how key concepts and actors within the discipline are introduced. In particular, we consider how articulations construct *behavioural science* as trustworthy or not, with a focus on its emergent scientific role in high-stake public policy. Here, we separated themes into (1) barriers and (2) drivers of trust and credibility.

We observed four barriers to trust and credibility: (1) human irrationality and citizen autonomy, (2) perceived conflicts of interest, (3) *behavioural science* as being no more than common sense, and (4) the sparse evidence base for key concepts associated with the science.

##### Human Irrationality and Citizen Autonomy

As one common theme in media discourse, effectiveness of *behavioural science* rests on humans acting irrationally. This theme is at times met with resistance in association with the perceptions that the drive for a national lockdown rested on a soft (subconscious) “nudge” to overcome non-compliance. This perception aligns with criticism of policy-initiated behaviour change as a threat to citizen autonomy (Jones et al., 2013; Leggett, 2014).

##### Perceptions Around Conflict of Interests

Second, we observe emphasis on semi-privatization of Dr. David Halpern and the BIT, in particular in the context of strong negative sentiment;

“David Halpern, head of the semi-privatized nudge unit advising Mr. Johnson on *behavioural science*…” (Parker and Hughes, 2020).

“David Halpern, of the part Government-owned Behavioural Insights Team…” (Malnick, 2020).

This is important as the initial coupling of the government's strategy with these actors shows to be paired with perceptions of being profit driven. Under high-stake policy making, this may represent a source of distrust, as previous studies show that unbiased, reliable and transparent knowledge is associated with independence of other interests (Hendriks et al., 2015; Pittinsky, 2015).

##### Perceptions of Behavioural Science as No More Than Common Sense

We observe *behavioural science* discussed (1) through questioning its evidence-based and readiness for policy application, but also (2) through the extent to which it is not just more than common sense knowledge;

“*Behavioural science* is not a science. The discipline has been hit by a “replication crisis” - results of even well-known studies cannot always be reproduced. Few experimental conditions can be controlled and it is often difficult even to define terms. With little way to prove their hunches wrong, behavioural scientists often assume they are right. That matters when the “science” is applied to policy decisions. While many of *behavioural science's* insights are mere common sense (people are more likely to turn up for GP appointments when you remind them to), they are dressed up as fact. […] Besides, behavioural scientists are lobbyists for their own brand of thinking. They are not impartial advisers, and it is time the government stopped treating them as such. They should ditch them altogether. There is evidence enough.” (Gill, 2020b).

“Without an all-out national mobilization plan for social distancing, are the UK government behavioural and nudge strategies really evidence-based to flatten the peak? Or simply based on models?” (Mullin, 2020).

The use of quotation marks (“”) around the word science was found in other articles, which functions to express, at best, reservation and at worst a sense of irony toward the perception that *behavioural science* is indeed scientific (Weizman, 2011). Criticisms is expedited as scientific experts are introduced as experts of *behavioural science* aligned with government, yet subsequently identify as an independent experts;

“Boris Johnson got his response to the pandemic “disastrously wrong” because he did not listen to *behavioural science* experts, a government adviser has said. Delaying lockdown because people would get tired of staying at home was “vigorously opposed” by behavioural scientists feeding into the Scientific Advisory Group for Emergencies, said Stephen Reicher, a member of the Scientific Pandemic Influenza Group on Behaviours, a committee of Sage. Taking a swipe at behavioural theories known as “nudge,” he said that one view of human behaviour may be “overly dominating in No 10,” leading to “bad decisions”.” (Smyth, 2020b).

##### Questioning the Scientific Evidence Base for Herd Immunity, Behavioural Fatigue or Nudge

Most commonly, the introduction of *behavioural science* center around the mention of “nudges,” “herd immunity,” and “behavioural fatigue;”

“If ‘behavioural fatigue' truly represents a key factor in the government's decision to delay high-visibility interventions, we urge the government to share an adequate evidence base in support of that decision. If one is lacking, we urge the government to reconsider these decisions,” wrote Prof. Ulrike Hahn from Birkbeck, University of London, and others.” (Boseley, 2020a).

“*Behavioural science* works on the basis that people don't always act rationally, and that “nudges” can be more effective at changing behaviour than diktats from authority.” (Coyle, 2020).

The mention of the above concepts frequently emphasizes concern over their scientific basis. We also observed frequent coupling of “nudge” “herd immunity” with public policy application, which in triad is widely criticized in pre- and early lockdown media coverage.

Taken together, these themes question the credibility of the discipline in informing policy and come together in Martha Gill's (2020b) framing of behavioural scientists as not being “impartial advisers,” but rather with disguised motives. Here too, we see the use of quotation marks to question the legitimacy of the scientific basis for psychology and nudge. This framing is crucial, as it is also Martha Gill's tweet that held the highest retweet value (over 900) across the time frame;

“This ‘science advisor' [Halpern] is a psychologist. I really can't believe we are attempting to 'nudge' our way out of this with soft science when we need hard science. Epidemiologists are the scientists to listen to.” (Gill, 2020a).

Other articles reveal facilitators of credibility and trust in *behavioural science*. We identify three themes: (1) scientists who alert to the misuse of scientific evidence in government, and (2) reference to *behavioural science's* ability to capture public opinion and (3) aid in transparent communication.

##### Alerting to the Misuse of Scientific Evidence in Government

These articles distinguished between scientific expertise offered by *behavioural science* experts, and how they were translated into government action. They alert that the government appropriated policy recommendations around communication and messaging, which in turn fostered trust in *behavioural science* from media;

“West also said there had been growing unease among his advisory colleagues about a divergence between the scientific advice and the government's approach. “Those of us on Spi-B have been increasingly concerned about the extent to which the government's approach to the *behavioural science* and the messaging, particularly, has been at 180 degrees from the kind of advice that we have been sending into the Cabinet Office,” said West. Members of Spi-B […]say their recommendations to set very clear and unequivocal messages for the public to follow have frequently been ignored by politicians.” (Boseley, 2020b).

##### Discussion of Capturing Public Opinion and Transparent Communication

In similar critique of government, there is emphasis on how *behavioural science* measures are useful for capturing public reactions to policy measures, and that the role of the discipline in understanding how to communicate with the public in a transparent and clear manner was seen as crucial for adherence to new measures, but that this was not taken on board by the government.

We conclude that barriers to trust and credibility arise from questions around the scientific nature of the *behavioural science*, and the purity of intention of behavioural scientists. Drivers of trust and credibility come from decoupling the discipline from the government's response and stressing its uses for public involvement in scientific practice. For this, criticism from behavioural scientists on the government's advisory board (SPI-B) plays a key role, as they stress having felt their advice being “trashed” (Boseley, 2020b) or “ignored,” echoing the positive sentiment found toward SPI-B in Study 1.

### Discussion

Study 3 provides three layers of insight. First, the UK COVID-19 policy choices were characterized as unique or divergent in some prominent media publications, with the UK lockdown policy described as delaying harder restrictions based on evidence from *behavioural science*. This is consistent with patterns in Study 1 and 2 whereby *behavioural science* as embedded in the UK policy response was frequently characterized by negative sentiment, whereas criticism about these same policies by prominent (independent) behavioural scientists was more often characterized by positive sentiment.

Second, we note that the media awards credibility to scientific evidence under high-stake policy making conditions (perceived to be) valid, transparent and reliable. In contrast, credibility is questioned when other scientific experts (from within or outside the discipline) critique public policies or the scientific evidence that support them. References to epidemiologists, public health experts, clinicians, immunologists were common, and in most instances these actors were presented in ways that lent credibility to their expertise. But if these actors were critical of public policies, this was often driven by questions on “which science” was guiding the choices of policy officials. Hereto (lack of) transparency in addition to a lack of collaborativeness seems to be a driver of outcry.

Third, we observe an additional lever of credibility and trust. Particular scientists from within the discipline may cry out to separate their identity from that of the negatively perceived subgroup. With the over-coupling between lockdown policies and *behavioural science* in the media, we observed an uprising against its characterization from closely linked experts. Here credibility is undermined by links to scientific actors thought to have conflicts of interest and question the extent to which their contributions can be evidence-based and unbiased. The contrast of independent and dependent scientists function to raise awareness of the potential problematic relationship between science and public policy, seen as favoring not the public, but private interests.

## General Discussion

### Summary of Findings

Using two distinct data sources (print media and Twitter chatter) and a mixed methodological design, we have mapped media and public discourse surrounding *behavioural science* contributions to the first UK lockdown decision of March 2020. We found two distinct clusters of actors and concepts in the *behavioural science* to be received differentially by both the media and public: BIT, Dr. David Halpern and “nudge” were viewed as embedded with the lockdown policy, coupled with negative perceptions; on the other hand, Prof. Susan Michie, Prof. Steven Reicher, and the SPI-B were perceived to be speaking out against these policies. Some of those amongst the second set of actors were also publicly associated with less policy-oriented *behavioural science* activity, surrounding psychological science and behaviour change, which was regarded as substantially more positive. The public eye, however, was drawn more to the conflict observed between behavioural scientists embedded with policy and those expressing concern over their choices. This, in turn, showed to affect the perceptions of *behavioural science* most substantially.

How do the *behavioural science* approaches differ between clusters? One distinction is that positive and neutral sentiment toward behaviour change and psychology was captured by work surrounding the enabling of citizen choice (e.g., handwashing, social distancing), whilst negative and divisive sentiment was associated with *behavioural science* applied to more embedded and politicized restriction of citizen choice (e.g., lockdown, rules of social isolation). Although this may be so, we also observed negative sentiment toward nudge for not being restrictive enough, so this does not seem to explain the divisive debate entirely. Another contrast between these clusters of actors and concepts was their perceived embedded vs. independent nature from political, as opposed to public, needs. A common issue with embedding scientific practice in policy making is the bias in selection of evidence to suit political needs (Cairney, 2020; Stevens, 2020). In addition, *behavioural science* as embedded in the COVID-19 policy response was heavily criticized by the media for lack of transparent practices. In contrast, when prominent (independent) behavioural scientists discussed behavioural research as a tool to facilitate public involvement and transparency, its use was rather applauded. Finally, upon closer inspection we note differences between clusters of actors in terms of their willingness to engage with the media. We expect this may have impacted in which light the media covered the actors. As a proxy for whether actors entertained media engagement, we reviewed whether actors were discussed through direct quotes, vs. talked about. We see that those who were quoted more (e.g., *Michie, Reicher*), seemed to have been discussed in a more positive light than those who were not (*Halpern, SPI-B;* see Supplementary Material 12).

### *Behavioural Science* and COVID-19 Response: Implications and Recommendations

In light of the barriers and drivers observed in relation to trust and credibility around the integration of *behavioural science* in national policy making under emergency constraints, we discuss recommendations for (1) informing transparent and ethical communication for future behavioural policy making and (2) their immediate use for shaping communication around the behavioural COVID-19 policy measures.

#### Make Behavioural Policy Ethical and Transparent

The extent to which *behavioural science* and the political philosophical tradition of libertarian paternalism are conflated, resulted in confusion and divisiveness in media and public discourse. In our data, we see that *behavioural science* and nudging are often conflated, paired with disagreement about the political philosophical implications of nudging principles, and negative sentiments toward policy applications of *behavioural science*. This was particularly marked during the initial phases of the COVID-19 response, where *behavioural science* was often associated negatively with “soft” approaches to managing the virus, or advocacy for (explicit or implicit) policies in favor of herd immunity. While our results are not conclusive about the perceived lack impact of this confusion for ongoing trust in *behavioural science* approaches in the context of public policy, we can conclude that it was a significant source of enduring negative sentiment toward *behavioural science* and behavioural scientists during this time period. Even if choice processes behind individual policy choices cannot be disclosed, we recommend that generalized processes could aid in perceived transparency. Related to this, confusions, conflations and sentiments need to be monitored and addressed directly by key public figures in the field in high stake contexts. Related to this, a substantial body of public opinion expressed concerns that *behavioural science* could be used in ways that are manipulative and/or bypassing citizen autonomy. On a longer term, we recommend that further efforts are made by leaders in the field to clarify the ethical features of different behavioural policy tools (e.g., Lades and Delaney, 2020), to embed such tools in day to day practice and to justify policy choices where suitable.

#### Clarify the Field of *Behavioural Science*

The development of behavioural-science driven approaches has been a marked feature of British public policy of the last decade. The integration of a *behavioural science* stream into the government COVID-19 response policy was debated heavily throughout its initial phases, but no mention was made of the heterogeneous perspectives that this reflects. The public representations we captured reflect a high degree of heterogeneity in the use of the discipline term to represent distinct perspectives and streams of research, discussed in separable clusters of association, something that in itself may have contributed to confusion among the public. Similarly, the extent to which *behavioural science* research is seen as a valuable input beyond lay intuitions about human behaviour is another important aspect of field clarification. Also, the readiness of various strands of *behavioural science* to contribute to emergency situations is another feature of public discourse that has also been reflected in recent academic debates (e.g., IJzerman et al., 2020; Lunn, 2020). Structured discussion among key public figures and institutions that use this phrase about the nature and historical origins of their work might be particularly helpful in resolving such confusion and clarifying distinctions between distinctive streams of thought. We hope the analysis in this paper could contribute to this process.

#### Define the Role of (Behavioural) Science in Policy Transparently

Overall, the public perception of *behavioural science* also displays a marked pattern of positivity, with both media and the public expressing positive sentiment. Positivity was mostly expressed in relation to the role of *behavioural science* and behavioural scientists in enabling protective health behaviours, improving citizen involvement in science and pandemic response policy overall.

We observe that the spread of negative sentiment was centered around a relatively small group of interconnected actors, and that negative sentiments about high stake policy decisions gained more traction than those linked to positive sentiment. It is beyond the scope of the current study to ascertain whether the perception of UK policy being markedly different from other countries due to *behavioural science* influence is a reflection of the actual policy process. Even if not, we recommend that a widespread perception of this nature is addressed in the short term, as it could have consequences for the acceptability of *behavioural science* in policy as well as potentially detracting from the consistency and perceived trustworthiness of its contribution to the emergency response.

Negative sentiment toward *behavioural science* and behavioural scientists link to the embeddedness of *behavioural science* within the lockdown policies of the UK, with suspicions that the “divergent” UK approach may have reflected insufficient separation between the science advice and political decision making. The extent to which the BIT's financial structure constrains their role in policy was also a feature of public discourse on *behavioural science* during this period. We recommend that establishing norms and expectations from the role of scientist, scientific advisor, policy maker, and advocate may be of help to the actor and public.

#### Implications for Current Pandemic Practice

(Behavioural) science teams working with government on pandemic response should increase efforts to explain the composition of their teams, engage with the public proactively and dynamically with media narratives on the role of science and its role in policy. Leaders in the field should continue to communicate the role of evidence in informing policy transparently, and where possible increase efforts to be seen as independent from political processes.

### Conclusion and Future Research

This study is based on analysis of public discourse in one country at a time of a major crisis. Future work comparing the discourse *behavioural science* across different global settings will give a fuller account of the developing influence of emergent *behavioural science* on policy. Furthermore, the current study is based on samples of print and social media. An interesting area of future study will be to compare discourse between types of newspapers, expand the timeframe of this search, other high stake policy contexts, or examine public attitudes and representations directly through surveys and interviews. Generally, an urgent task highlighted by the study of this COVID-19 policy response, is to continue efforts at field definition and role clarification in the (behavioural) sciences more globally.

## Data Availability Statement

The raw data supporting the conclusions of this article will be made available by the authors, without undue reservation.

## Author Contributions

JS, AT, and SO designed Study 1, 2, and 3. AT lead data processing and analysis for Study 1 and 2. SO lead the data analysis for Study 3. IM, SO, AT, and JS contributed to sentiment coding for Study 1 and 2. JS, AT, SO, IM, and LD contributed to discussions and writing the paper. All authors contributed to the article and approved the submitted version.

## Conflict of Interest

The authors declare that the research was conducted in the absence of any commercial or financial relationships that could be construed as a potential conflict of interest.

## Footnotes

^1^We note that discussion of key actors is not against any law of data protection.

^2^Note the dice coefficient is influenced by co-occurrence, but also by the individual frequency of the two keywords. Thus, the Dice coefficient can be high even when the co-occurrence is relatively small. For example, if two keywords have overall low frequency but they almost always appear together whenever they appear in an article. To minimize any misrepresentations, we only used the dice coefficient analysis for keywords *n* > 20.

^3^For instance, if in a given 2-week period 4 tweets were published, each with a certain number of retweets, and KwordA appeared in 3 of them as follow: tweet 1 | retweets: 10 | kwordA: 1; tweet 2 | retweets: 5 | kwordA: 1; tweet 3 | retweets: 0 | kwordA: 1; tweet 4 | retweets: 1 | kwordA: 0. Salience (original tweets only) for kwordA in this fortnight would be: 3/4 = 0.75. Salience (incl. retweets) for kwordA in this fortnight would be: [(1 + 10) + (1 + 5) + (1 + 0)] / (4 + 16) = 18/20 = 0.9.
