# Supplementary material for: Lessons From the UK's Lockdown: Discourse on Behavioural Science in Times of COVID-19
Source: Front Psychol. 2021 Jun 17;12:647348. doi: 10.3389/fpsyg.2021.647348 (PMC8247580; doi:10.3389/fpsyg.2021.647348)
Supplement: Supplementary file 1 [file Data_Sheet_1.PDF]

7.1 Supplementary Material 1: Table displaying circulation figures by newspaper for 2020 and the newspapers used for the quantitative analysis of Study 1.

**Newspaper**	**TBR***	**-4**	**-3**	**-2**	**-1**	**0**	**1**	**2**	**3**	**4**	**5**	**6**	**7**
		**27/1--9/2**	**10/2-23/2**	**24/2-8/3**	**9/3-22/3**	**23/3-5/4**	**6/4-19/4**	**20/4-3/5**	**4/5-17/5**	**18/5-31/5**	**1/6-14/6**	**15/6-28/6**	**29/6-12/6**
City A.M.	85,738	1	1		2	1							
Daily Express	289,679							1					
Daily Mirror	441,934		2	1	6	2	1	1	21	9	2		
Daily Record	103,222				2	1	2	1	3				
Daily Star	432,909												
Evening Standard	787,447	1		2	4					4		3	
Financial Times	155,009	2	1	6	13	24	11		11	1	2	2	2
i	215,932			3	4		4	2	4	3	3	1	1
Metro	1,419,614			1					2	1		3	1
*The Telegraph***	566,105			1	12	24	28	38	27	8	2	3	2
The Guardian	281,900	2	2	2	20	13	4	19	20	30	8	10	9
The Independent	13,908	3			7	12	8	10	4	7	1	4	2
The Mail	2,200,207	1	3	4	15	3	3			14	15	10	11
The Sun	1,206,595			1	1	8	3	4	2	5	2		1
The Times	359,960	1	5	11	47	17	39	26	36	11	10	8	5
		**11**	**14**	**32**	**133**	**105**	**103**	**102**	**130**	**93**	**45**	**44**	**34**
***Circulation numbers for February 2020 taken from ABC for average monthly circulation.*													
***Not audited by ABC from February 2020, circulation number based on January 2020*													
