# Supplementary material for: Lessons From the UK's Lockdown: Discourse on Behavioural Science in Times of COVID-19
Source: Front Psychol. 2021 Jun 17;12:647348. doi: 10.3389/fpsyg.2021.647348 (PMC8247580; doi:10.3389/fpsyg.2021.647348)
Supplement: Supplementary file 2 [file Data_Sheet_2.PDF]

7.2 Supplementary Material 2: Defining the A) *Lexis Nexis* query on the topic of 'behavioural science' between 20th of January and 10th of May and B) for the 3rd March and the 9th June Twitter data archive

Step 1: Generation of common terms through exploratory reading. Based on exploratory reading of articles on behavioural science we arrived at the following search terms: *behavi*ral scien! OR behavi*ral insight! OR behavi*r change OR nudg! OR behavi*r OR psycholog! OR behavi*ral analys! OR behavi*ral econom! OR behavi*ral policy OR irrational behavi*r OR libertarian paternalis! OR paternal! OR choice architec! OR nudge theor! OR nudge strateg! OR behavi*ral fatigue OR herd immunity OR david halpern OR susan michie OR richard thaler OR cass sunstein OR dan* kahneman OR richard amlot OR spi-b OR behavioural insights team OR nudge unit*

Step 2: Systematic generation of overlooked key actors and search terms. To identify additional relevant terms for our query, we used two methods. First, we qualitatively reviewed a random sample of articles that were returned in Step 1. This resulted in the addition of '*nick chate*r' and '*nanny state*'. Next, to identify potential key behavioural science actors that we could have missed (or should not have been in the original search), we went through all the SPI-B members (https://bit.ly/2HHv8Et). To identify the actors most likely to influence behavioural science in the high-stake policy context *and* be deemed a relevant actor in the public sphere, we refined this search by consulting the overarching SAGE board (https://bit.ly/2HHv8Et). This step in our search took place on 10 May 2020, and returned: Susan Michie, David Halpern, Theresa Marteau, Lucy Yardley, Charlotte Watts, Brooke Rogers, and Russell Viner. For these actors (as well as for all the actors that were already in our query) we ran a *Lexis Nexis* search one at a time between 20 Jan 2020 and 10 May 2020 in combination with the broadest terms we deemed relevant to the behavioural sciences: '*AND behav! OR nudg! OR SPI-B*' to identify which actors would be relevant to add into the final query. This did not result in additional actors, but one actor ('*richard amlot'*) was removed.

In sum, this step resulted in the following search: *behavi*ral scien! OR behavi*ral insight! OR behavi*r change OR nudg! OR behavi*r OR psycholog! OR behavi*ral analys! OR behavi*ral econom! OR behavi*ral policy OR irrational behavi*r OR libertarian paternalis! OR paternal! OR choice architec! OR nudge theor! OR nudge strateg! OR behavi*ral fatigue OR herd immunity OR david halpern OR susan michie OR richard thaler OR cass sunstein OR dan* kahneman OR spi-b OR behavioural insights team OR nudge unit OR nick chater OR nanny state*

Step 3: Replacements of keywords that generated frequent irrelevant results. As Step 1 also generated a high number of irrelevant results based on search terms *'nudg!', 'herd immunity', 'behavi*r', 'paternal!', 'nanny state', 'psycholog!', 'behavi*r change*' and *'irrational behaviour'*, we opted to only include these terms in any combination with one another in the final query if 1) it generated at least one article above and beyond the original query and 2) this generated articles were deemed relevant to the theme of behavioural science, as agreed by three independent coders.

This resulted in the replacement of *nudg!' OR 'herd immunity' OR 'behavi*r' OR 'paternal!' OR 'nanny state'* with *(herd immunity AND behavi*r AND nudg!) OR (nanny state AND behavi*r AND nudg!).* In addition we identified that '*psycholog*!' and *'behavi*r'* resulted in a few novel relevant articles when combined with *'nudg!*: *(psycholog! /2 nudg!) OR (behavi*r! /2 nudg!)*. NOTE: '*nudge strateg!* and '*nudge unit*' were already in the original query. Most notably, we attempted a number of combinations with '*psycholog!*' and

*'behavi*r'* without resulting in relevant additional findings above and beyond the updated query. Similarly, searches for *'behavi*r change'* and '*irrational behavi*r*' in combination with any of the above search terms, did not result in relevant additions to the corpus.

This brought the query to: *behavi*ral scien! OR behavi*ral insight! OR behavi*ral analys! OR behavi*ral econom! OR behavi*ral policy OR libertarian paternalis! OR paternal! OR choice architec! OR nudge theor! OR nudge strateg! OR behavi*ral fatigue OR herd immunity OR david halpern OR susan michie OR richard thaler OR cass sunstein OR dan* kahneman OR spi-b OR behavioural insights team OR nudge unit OR nick chater OR (herd immunity AND behavi*r AND nudg!) OR (nanny state AND behavi*r AND nudg!) OR (psycholog! /2 nudg!) OR (behavi*r! /2 nudg!)*

To refine the relevance of the corpus even further, we finally added two '*exclusions*' to the query: **'***judge david halpern***'** and '*criminal behavi*ral analys!*'.

Supplementary Material 2.B: Defining the search query for the Twitter data archive

We extracted the tweets from Coronavirus Tweet Ids Version 7 dataset (Kerchner & Wrubel, 2020) from TweetSets, the archive of Twitter datasets for research and archiving managed by George Washing University (Littman, 2008). The Coronavirus dataset contains the tweet IDs of 239,861,658 tweets related to COVID-19, collected between March 3, 2020 and June 9, 2020 from the Twitter API using the tags "coronavirus", "COVID-19", "epidemiology", "pandemic".

Only tweets whose text contained <u>at least one of</u> these terms were retrieved (comma separate multiple terms, space separated words were treated as a unique phrase):

nudge theory, david halpern, nick chater, susan michie, richard thaler, cass sunstein, dan kahneman, daniel kahneman, behavioural science, behaviour change, behavioural scientist, behavioural insight, libertarian paternalism, choice architecture, choice architect, behavioural analysis, behavioural analyst, behavioural insights team, nudge unit, behavioural economics, behavioural economist, behavioural policy, behavioural fatigue, herd immunity behaviour, herd immunity behavior, herd immunity behavioural science, herd immunity nudg, herd immunity nudge, herd immunity nudging, herd immunity nudge unit, herd immunity nudge theory, herd immunity behavioural, nudge strategy, nanny state behaviour, nanny state nudg, nudgetheory, davidhalpern, nickchater, susanmichie, richardthaler, casssunstein, dankahneman, danielkahneman, behaviouralscience, behaviourchange, behaviouralscientist, behaviouralinsight, libertarianpaternalism, choicearchitecture, choicearchitect, behaviouralanalysis, behaviouralanalyst, behaviouralinsightsteam, nudgeunit, behaviouraleconomics, behaviouraleconomist, behaviouralpolicy, behaviouralfatigue, herdimmunity behaviouralscience, nudgestrategy, nannystate behaviour, nannystate nudg

Unlike for the news article search, which was filtered for UK relevance at source by selecting British newspapers - an option not available for Twitter, for the Twitter data we tried to limit the number of tweets that were non UK-relevant by only including the British spelling of search terms when an American spelling was also possible (e.g., *behavioural science*).

In the Twitter query we also included the term *behaviour change*. Unlike the source database for the news articles, the source Coronavirus Tweet Ids dataset was already prefiltered for tweets related to COVID-19 and so to the relevant high-stake policy context.

Finally, we included a version of the multi-word search terms without the space inbetween words (e.g., *nudgetheory*), as this is how twitter users often write hashtags or long phrases in order to respect Twitter character limit.
