# Supplementary material for: Lessons From the UK's Lockdown: Discourse on Behavioural Science in Times of COVID-19
Source: Front Psychol. 2021 Jun 17;12:647348. doi: 10.3389/fpsyg.2021.647348 (PMC8247580; doi:10.3389/fpsyg.2021.647348)
Supplement: Supplementary file 3 [file Data_Sheet_3.PDF]

***7.3 Supplementary Material 3:* Table of the codebook for primary and secondary keywords used for analysis.**

**Keyword domain**	**Primary keywords**	**Secondary keywords**
disciplines	behavioural science	behavioural_science<br>behavioural_sciences<br>behavioural_policy<br>behavioural_scientists
	behavioural economics	behavioural_economics<br>behavioural_economists
	psychology	psychologists<br>psychology<br>psychological_science<br>psychological_policy
behavioural science concepts	behavioural analysis	behavioural_analysis<br>behavioural_analyst<br>behavioural_analysts
	behavioural change	behaviour_change<br>behavioural_change
	behavioural insight	behavioural_insight<br>behavioural_insights
	choice architecture	choice_architecture<br>choice_architect
	irrational behaviour	irrational_behaviour<br>irrational_choice<br>irrational_choices
	nudge	nudges<br>nudge<br>nudging<br>nudge_theory<br>nudge_strategy<br>paternalism<br>libertarian_paternalism<br>paternalistic
named actors	behavioural insights team	behavioural_insights_team<br>nudge_unit
	chater	chater
	halpern	halpern
	kahneman	kahneman
	michie	michie
	SPI-B	SPI-B
	sunstein	sunstein

	thaler	thaler
unnamed actors	behavioural economist	behavioural economist
	behavioural scientist	behavioural scientist
	psychologist	psychologist
