# Supplementary material for: Lessons From the UK's Lockdown: Discourse on Behavioural Science in Times of COVID-19
Source: Front Psychol. 2021 Jun 17;12:647348. doi: 10.3389/fpsyg.2021.647348 (PMC8247580; doi:10.3389/fpsyg.2021.647348)
Supplement: Supplementary file 4 [file Data_Sheet_4.PDF]

7.4 Supplementary Material 4: Trends in Salience and Sentiment for keywords per fortnight in newspaper articles (Study 1).

			Sentiment						Salience					
			Sentiment count			Prop sentiments			Normalized TF*relative DF	Difference	normalise TF (per 10,000 words)	relative DF	Term frequency (TF)	Document Frequency (DF)
			neg	neu	pos	neg	neu	pos						
Keyword	Fortnight starting	Fortnight to lockdown												
Behaviour change	2020-01-27	-4	0	0	0				0.00		0.00	0.00	0	0
	2020-02-10	-3	0	0	0				0.00	0.00	0.00	0.00	0	0
	2020-02-24	-2	0	0	0				0.00	0.00	0.00	0.00	0	0
	2020-03-09	-1	0	4	1	0.0	0.8	0.2	0.04	0.04	1.51	0.03	5	3
	2020-03-23	0	0	10	1	0.0	1.31	0.09	1.13	1.09	6.38	0.18	11	11
	2020-04-06	1	0	3	0	0.0	1.0	0.0	0.10	-1.03	2.08	0.05	3	3
	2020-04-20	2	0	6	0	0.0	1.0	0.0	0.15	0.05	2.68	0.06	6	4
	2020-05-04	3	0	8	0	0.0	1.0	0.0	0.25	0.10	3.14	0.08	8	8
	2020-05-18	4	0	3	0	0.0	1.0	0.0	0.04	-0.21	1.12	0.04	3	3
	2020-06-01	5	0	4	0	0.0	1.0	0.0	0.32	0.28	3.16	0.10	4	4
	2020-06-15	6	0	1	0	0.0	1.0	0.0	0.02	-0.30	0.97	0.03	1	1
	2020-06-29	7	0	4	0	0.0	1.0	0.0	0.52	0.49	3.86	0.13	4	4
Behavioural Economics	2020-01-27	-4	1	0	0	1.0	0.0	0.0	0.43		4.32	0.10	1	1
	2020-02-10	-3	0	1	1	0.0	0.5	0.5	0.55	0.11	7.10	0.08	2	1
	2020-02-24	-2	1	0	0	1.0	0.0	0.0	0.07	-0.48	1.70	0.04	1	1

frontiers

	2020-03-09	-1	0	3	0	0.0	1.0	0.0	0.03	-0.04	0.91	0.03	3	3
	2020-03-23	0	1	4	1	0.17	1.07	0.17	0.34	0.31	3.48	0.10	6	6
	2020-04-06	1	0	6	0	0.0	1.0	0.0	0.40	0.06	4.15	0.10	6	6
	2020-04-20	2	1	4	0	0.2	0.8	0.0	0.06	-0.33	2.23	0.03	5	2
	2020-05-04	3	0	2	2	0.0	0.5	0.5	0.06	0.00	1.57	0.04	4	4
	2020-05-18	4	2	2	1	0.4	0.4	0.2	0.11	0.05	1.87	0.06	5	5
	2020-06-01	5	0	0	0				0.00	-0.11	0.00	0.00	0	0
	2020-06-15	6	1	0	0	1.0	0.0	0.0	0.02	0.02	0.97	0.03	1	1
	2020-06-29	7	0	2	0	0.0	1.0	0.0	0.13	0.10	1.93	0.07	2	2
Behavioural Insights Team	2020-01-27	-4	0	1	1	0.0	0.5	0.5	0.86		8.64	0.10	2	1
	2020-02-10	-3	1	0	2	0.33	0.0	1.07	0.82	-0.04	10.65	0.08	3	1
	2020-02-24	-2	2	8	0	0.2	0.8	0.0	3.92	3.10	17.00	0.23	10	6
	2020-03-09	-1	15	29	8	0.29	0.56	0.15	4.38	0.45	15.75	0.28	52	30
	2020-03-23	0	1	9	9	0.05	0.47	0.47	1.42	-2.95	11.02	0.13	19	8
	2020-04-06	1	3	3	0	0.5	0.5	0.0	0.33	-1.09	4.15	0.08	6	5
	2020-04-20	2	3	13	0	0.19	1.21	0.0	0.90	0.57	7.13	0.13	16	9
	2020-05-04	3	1	9	3	0.08	1.09	0.23	0.55	-0.35	5.10	0.11	13	11
	2020-05-18	4	0	4	0	0.0	1.0	0.0	0.05	-0.50	1.50	0.04	4	3
	2020-06-01	5	1	0	0	1.0	0.0	0.0	0.02	-0.03	0.79	0.03	1	1
	2020-06-15	6	0	7	1	0.0	1.28	0.12	0.39	0.37	7.75	0.05	8	2
	2020-06-29	7	0	0	0				0.00	-0.39	0.00	0.00	0	0

Behavioural Science	2020-01-27	-4	0	0	0				0.00		0.00	0.00	0	0
	2020-02-10	-3	0	2	1	0.0	1.07	0.33	2.46	2.46	10.65	0.23	3	3
	2020-02-24	-2	3	6	7	0.19	0.38	0.44	14.64	12.19	27.19	0.54	16	14
	2020-03-09	-1	22	32	16	0.31	0.46	0.23	10.99	-3.65	21.20	0.52	70	56
	2020-03-23	0	17	17	12	0.37	0.37	0.26	13.77	2.77	26.68	0.52	46	32
	2020-04-06	1	5	33	13	0.1	1.05	0.25	19.62	5.85	35.31	0.56	51	35
	2020-04-20	2	7	35	5	0.15	1.14	0.11	10.92	-8.69	20.96	0.52	47	37
	2020-05-04	3	2	27	10	0.05	1.09	0.26	5.10	-5.82	15.30	0.33	39	34
	2020-05-18	4	2	41	11	0.04	1.16	0.2	11.47	6.37	20.25	0.57	54	47
	2020-06-01	5	2	19	5	0.08	1.13	0.19	11.57	0.10	20.51	0.56	26	22
	2020-06-15	6	14	25	17	0.25	0.45	0.3	36.60	25.03	54.22	0.68	56	27
	2020-06-29	7	1	12	3	0.06	1.15	0.19	7.21	-29.39	15.45	0.47	16	14
Behavioural Scientist	2020-01-27	-4	0	3	0	0.0	1.0	0.0	3.89		12.95	0.30	3	3
	2020-02-10	-3	0	1	0	0.0	1.0	0.0	0.27	-3.61	3.55	0.08	1	1
	2020-02-24	-2	0	1	0	0.0	1.0	0.0	0.07	-0.21	1.70	0.04	1	1
	2020-03-09	-1	0	4	3	0.0	0.57	0.43	0.14	0.07	2.12	0.06	7	7
	2020-03-23	0	1	5	1	0.14	1.11	0.14	0.39	0.26	4.06	0.10	7	6
	2020-04-06	1	0	8	1	0.0	1.29	0.11	0.89	0.50	6.23	0.14	9	9
	2020-04-20	2	0	8	0	0.0	1.0	0.0	0.40	-0.49	3.57	0.11	8	8
	2020-05-04	3	2	8	0	0.2	0.8	0.0	0.38	-0.02	3.92	0.10	10	10

	2020-05-18	4	0	7	1	0.0	1.28	0.12	0.29	-0.10	3.00	0.10	8	8
	2020-06-01	5	0	1	1	0.0	0.5	0.5	0.08	-0.21	1.58	0.05	2	2
	2020-06-15	6	1	5	1	0.14	1.11	0.14	1.02	0.94	6.78	0.15	7	6
	2020-06-29	7	0	5	0	0.0	1.0	0.0	0.80	-0.21	4.83	0.17	5	5
Halpern	2020-01-27	-4	0	0	0				0.00		0.00	0.00	0	0
	2020-02-10	-3	0	0	0				0.00	0.00	0.00	0.00	0	0
	2020-02-24	-2	0	0	0				0.00	0.00	0.00	0.00	0	0
	2020-03-09	-1	10	24	2	0.28	1.07	0.06	2.42	2.42	10.90	0.22	36	24
	2020-03-23	0	4	7	5	0.25	0.44	0.31	1.50	-0.93	9.28	0.16	16	10
	2020-04-06	1	0	3	0	0.0	1.0	0.0	0.03	-1.46	2.08	0.02	3	1
	2020-04-20	2	4	5	1	0.4	0.5	0.1	0.13	0.09	4.46	0.03	10	2
	2020-05-04	3	0	7	0	0.0	1.0	0.0	0.16	0.04	2.75	0.06	7	6
	2020-05-18	4	1	2	0	0.33	1.07	0.0	0.04	-0.12	1.12	0.04	3	3
	2020-06-01	5	0	0	0				0.00	-0.04	0.00	0.00	0	0
	2020-06-15	6	4	1	3	0.5	0.12	0.38	0.19	0.19	7.75	0.03	8	1
	2020-06-29	7	0	0	0				0.00	-0.19	0.00	0.00	0	0
Michie	2020-01-27	-4	0	0	0				0.00		0.00	0.00	0	0
	2020-02-10	-3	0	0	0				0.00	0.00	0.00	0.00	0	0
	2020-02-24	-2	0	0	0				0.00	0.00	0.00	0.00	0	0

	2020-03-09	-1	2	29	13	0.05	1.06	0.3	1.48	1.48	13.33	0.11	44	12
	2020-03-23	0	0	15	1	0.0	1.34	0.06	1.80	0.32	9.28	0.19	16	12
	2020-04-06	1	0	15	1	0.0	1.34	0.06	0.88	-0.92	11.08	0.08	16	5
	2020-04-20	2	0	29	2	0.0	1.34	0.06	3.31	2.43	13.82	0.24	31	17
	2020-05-04	3	2	26	0	0.07	1.33	0.0	2.26	-1.05	10.98	0.21	28	21
	2020-05-18	4	0	17	1	0.0	1.34	0.06	1.22	-1.04	6.75	0.18	18	15
	2020-06-01	5	0	22	1	0.0	1.36	0.04	5.12	3.90	18.14	0.28	23	11
	2020-06-15	6	0	17	0	0.0	1.0	0.0	2.88	-2.24	16.46	0.18	17	7
	2020-06-29	7	0	13	0	0.0	1.0	0.0	3.35	0.47	12.55	0.27	13	8
Nudge	2020-01-27	-4	1	0	6	0.14	0.0	1.26	15.11		30.22	0.50	7	5
	2020-02-10	-3	3	2	4	0.33	0.22	0.44	9.83	-5.29	31.94	0.31	9	4
	2020-02-24	-2	2	1	0	1.07	0.33	0.0	0.59	-9.24	5.10	0.12	3	3
	2020-03-09	-1	10	8	7	0.4	0.32	0.28	0.98	0.39	7.57	0.13	25	14
	2020-03-23	0	10	0	5	1.07	0.0	0.33	1.68	0.70	8.70	0.19	15	12
	2020-04-06	1	7	3	6	0.44	0.19	0.38	1.05	-0.63	11.08	0.10	16	6
	2020-04-20	2	9	6	3	0.5	0.33	0.17	0.90	-0.15	8.03	0.11	18	8
	2020-05-04	3	2	0	0	1.0	0.0	0.0	0.02	-0.89	0.78	0.02	2	2
	2020-05-18	4	1	3	4	0.12	0.38	0.5	0.11	0.09	3.00	0.04	8	3
	2020-06-01	5	4	0	2	1.07	0.0	0.33	0.12	0.01	4.73	0.03	6	1
	2020-06-15	6	1	2	0	0.33	1.07	0.0	0.22	0.10	2.90	0.08	3	3

	2020-06-29	7	0	1	0	0.0	1.0	0.0	0.03	-0.19	0.97	0.03	1	1
Psychologist	2020-01-27	-4	0	0	0				0.00		0.00	0.00	0	0
	2020-02-10	-3	0	1	1	0.0	0.5	0.5	1.09	1.09	7.10	0.15	2	2
	2020-02-24	-2	0	3	1	0.0	1.15	0.25	0.78	-0.31	6.80	0.12	4	3
	2020-03-09	-1	0	8	0	0.0	1.0	0.0	0.11	-0.67	2.42	0.05	8	5
	2020-03-23	0	0	5	1	0.0	1.23	0.17	0.28	0.17	3.48	0.08	6	5
	2020-04-06	1	0	7	1	0.0	1.28	0.12	0.62	0.33	5.54	0.11	8	7
	2020-04-20	2	0	9	1	0.0	0.9	0.1	0.57	-0.05	4.46	0.13	10	9
	2020-05-04	3	0	8	0	0.0	1.0	0.0	0.25	-0.32	3.14	0.08	8	8
	2020-05-18	4	0	8	0	0.0	1.0	0.0	0.25	0.01	3.00	0.08	8	7
	2020-06-01	5	0	4	0	0.0	1.0	0.0	0.32	0.07	3.16	0.10	4	4
	2020-06-15	6	0	6	1	0.0	1.26	0.14	1.19	0.86	6.78	0.18	7	7
	2020-06-29	7	0	7	0	0.0	1.0	0.0	1.35	0.17	6.76	0.20	7	6
Psychology	2020-01-27	-4	0	1	0	0.0	1.0	0.0	0.43		4.32	0.10	1	1
	2020-02-10	-3	0	0	0				0.00	-0.43	0.00	0.00	0	0
	2020-02-24	-2	0	2	0	0.0	1.0	0.0	0.26	0.26	3.40	0.08	2	2
	2020-03-09	-1	0	23	7	0.0	1.17	0.23	2.02	1.76	9.09	0.22	30	24
	2020-03-23	0	1	5	5	0.09	0.45	0.45	0.82	-1.20	6.38	0.13	11	8
	2020-04-06	1	1	11	2	0.07	1.19	0.14	1.54	0.72	9.69	0.16	14	10
	2020-04-20	2	3	19	0	0.14	1.26	0.0	2.63	1.09	9.81	0.27	22	19
	2020-05-04	3	2	20	2	0.08	1.23	0.08	1.85	-0.78	9.42	0.20	24	20

	2020-05-18	4	0	24	5	0.0	1.23	0.17	2.36	0.51	10.87	0.22	29	18
	2020-06-01	5	0	10	0	0.0	1.0	0.0	1.42	-0.94	7.89	0.18	10	7
	2020-06-15	6	0	12	5	0.0	1.11	0.29	3.29	1.88	16.46	0.20	17	8
	2020-06-29	7	0	5	0	0.0	1.0	0.0	0.64	-2.65	4.83	0.13	5	4
Spi-B	2020-01-27	-4	0	0	0				0.00		0.00	0.00	0	0
	2020-02-10	-3	0	0	0				0.00	0.00	0.00	0.00	0	0
	2020-02-24	-2	0	0	0				0.00	0.00	0.00	0.00	0	0
	2020-03-09	-1	0	4	0	0.0	1.0	0.0	0.02	0.02	1.21	0.02	4	2
	2020-03-23	0	1	0	1	0.5	0.0	0.5	0.04	0.01	1.16	0.03	2	2
	2020-04-06	1	0	2	1	0.0	1.07	0.33	0.10	0.06	2.08	0.05	3	3
	2020-04-20	2	0	16	0	0.0	1.0	0.0	0.90	0.81	7.13	0.13	16	9
	2020-05-04	3	4	58	8	0.06	1.23	0.11	6.73	5.83	27.46	0.25	70	25
	2020-05-18	4	0	37	6	0.0	1.26	0.14	4.27	-2.46	16.12	0.27	43	22
	2020-06-01	5	0	10	2	0.0	1.23	0.17	2.18	-2.09	9.47	0.23	12	9
	2020-06-15	6	0	7	0	0.0	1.0	0.0	1.02	-1.17	6.78	0.15	7	6
	2020-06-29	7	0	9	0	0.0	1.0	0.0	0.87	-0.15	8.69	0.10	9	3
