# Supplementary material for: Lessons From the UK's Lockdown: Discourse on Behavioural Science in Times of COVID-19
Source: Front Psychol. 2021 Jun 17;12:647348. doi: 10.3389/fpsyg.2021.647348 (PMC8247580; doi:10.3389/fpsyg.2021.647348)
Supplement: Supplementary file 5 [file Data_Sheet_5.PDF]

7.5 *Supplementary Material 5*: Overall frequency of primary keywords A) in newspaper articles (Study 1) and B) on Twitter (Study 2).

A)

Primary Keyword	Overall frequency	Included in analysis
behavioural science	424	Y
michie	206	Y
spi-b	166	Y
psychology	165	Y
behavioural_inisghts_team	134	Y
nudge	113	Y
halpern	83	Y
psychologist	72	Y
behavioural scientist	68	Y
behavioural change	45	Y
behavioural economics	36	Y
kahneman	27	N
behavioural insight	18	N
sunstein	16	N
thaler	13	N
behavioural analysis	9	N
behavioural economist	8	N
chater	2	N
irrational behaviour	0	N
choice architecture	0	N

B)

Keyword	Frequency	Included in analysis
behav_change	722	Y
behavioural science	644	Y
nudge	243	Y
behavioural insights team	233	Y
michie	232	Y
psychology	181	Y
halpern	99	Y
sunstein	82	N
behavioural economics	67	Y
behavioural scientist	51	Y
psychologist	44	Y
behavioural insight	32	N
kahneman	28	N
behavioural economist	15	N
behavioural analysis	10	N
choice architecture	10	N
thaler	10	N
chater	9	N
spi-b	5	Y
irrational behaviour	0	N
