# Supplementary material for: Lessons From the UK's Lockdown: Discourse on Behavioural Science in Times of COVID-19
Source: Front Psychol. 2021 Jun 17;12:647348. doi: 10.3389/fpsyg.2021.647348 (PMC8247580; doi:10.3389/fpsyg.2021.647348)
Supplement: Supplementary file 6 [file Data_Sheet_6.PDF]

7.6 Supplementary Material 6: The Dice coefficient formula and co-occurrence strength between keyword pairs per time-period. Pairs are reported in descending order of association in pre-lockdown.

		Co-occurrence strength (Dice coefficient) top10 in each period in bold			Which co-occurrences have stayed / started / ended:	
**keyword1**	**keyword2**	**Pre Lockdown**	**During Lockdown**	**Post Lockdown**	**during vs pre lockdown?**	**post vs during lockdown?**
michie	professor	0.62	0.46	0.48	stayed	stayed
dr	halpern	0.55	0.50	-	stayed	ended
behav_insights_team	halpern	0.51	0.34	-	stayed	ended
halpern	head	0.46	-	-	ended	none
psychology	health	0.45	0.38	0.38	stayed	stayed
michie	health	0.44	0.24	0.26	stayed	stayed
michie	university_college	0.44	0.48	0.40	stayed	stayed
michie	london	0.41	0.37	0.38	stayed	stayed
michie	governments	0.36	-	0.21	ended	started
behav_insights_team	head	0.34	-	-	ended	none
psychology	professor	0.33	-	-	ended	none
governments	behav_insights_team	0.28	-	-	ended	none
michie	psychology	0.27	0.17	-	stayed	ended
science	behav_science	0.20	-	-	ended	none
government	behav_science	0.19	-	-	ended	none
advice	behav_science	0.19	-	0.32	ended	started
people	behav_science	0.18	-	-	ended	none
paper	spi-b	-	0.21	-	started	ended
sage	behav_science	-	0.13	0.13	started	stayed
sage	spi-b	-	0.25	-	started	ended
social	psychology	-	0.26	0.36	started	stayed
behav_science	spi-b	-	0.10	0.13	started	stayed
behav_science	people	-	0.26	0.22	started	stayed
behav_science	governments	-	0.11	0.25	started	stayed
behav_science	lockdown	-	0.17	0.15	started	stayed
behav_science	professor	-	0.19	0.12	started	stayed
behav_science	economics	-	0.15	-	started	ended
behav_science	public	-	0.18	0.11	started	stayed

behav_science	pandemic	-	0.14	-	started	ended
behav_science	measures	-	0.10	-	started	ended
behav_science	influenza	-	0.10	-	started	ended
behav_science	group	-	0.21	0.16	started	stayed
behav_science	government	-	0.21	0.22	started	stayed
behav_science	restrictions	-	0.13	-	started	ended
behav_science	scientific	-	0.12	-	started	ended
spi-b	april	-	0.23	-	started	ended
spi-b	people	-	0.12	0.23	started	stayed
spi-b	public	-	0.17	0.21	started	stayed
spi-b	pandemic	-	0.39	0.44	started	stayed
spi-b	influenza	-	0.44	0.48	started	stayed
spi-b	group	-	0.38	0.49	started	stayed
spi-b	government	-	0.17	0.21	started	stayed
spi-b	behaviours	-	0.25	0.41	started	stayed
spi-b	scientific	-	0.42	0.47	started	stayed
michie	people	-	0.29	-	started	ended
michie	director	-	0.30	0.27	started	stayed
michie	prof	-	0.55	0.46	started	stayed
michie	centre	-	0.29	0.29	started	stayed
michie	member	-	0.21	0.32	started	stayed
michie	group	-	0.22	0.23	started	stayed
michie	government	-	0.23	-	started	ended
michie	behav_change	-	0.36	0.28	started	stayed
people	nudge	-	0.18	-	started	ended
people	behav_scientist	-	0.15	-	started	ended
people	psychology	-	0.13	-	started	ended
people	behav_change	-	0.20	-	started	ended
university_college	psychology	-	0.23	0.25	started	stayed
university_college	behav_change	-	0.57	-	started	ended
professor	psychology	-	0.33	0.45	started	stayed
professor	behav_change	-	0.30	-	started	ended
university	psychology	-	0.30	0.34	started	stayed
director	behav_change	-	0.69	0.89	started	stayed
london	psychology	-	0.27	0.26	started	stayed

london	behav_change	-	0.41	-	started	ended
centre	behav_change	-	0.73	0.83	started	stayed
sage	michie	-	-	0.18	none	started
advice	spi-b	-	-	0.21	none	started
behav_science	advisory_group	-	-	0.18	none	started
behav_science	johnson	-	-	0.14	none	started
behav_science	experts	-	-	0.18	none	started
behav_science	university	-	-	0.13	none	started
behav_science	ministers	-	-	0.11	none	started
behav_science	reicher	-	-	0.11	none	started
behav_science	member	-	-	0.21	none	started
behav_science	professor_reicher	-	-	0.12	none	started
behav_science	st_andrews	-	-	0.13	none	started
behav_science	new	-	-	0.13	none	started
behav_science	psychology	-	-	0.12	none	started
behav_science	covid-19	-	-	0.13	none	started
spi-b	governments	-	-	0.23	none	started
spi-b	london	-	-	0.18	none	started
spi-b	member	-	-	0.38	none	started
spi-b	covid-19	-	-	0.20	none	started
michie	scientific_advisory	-	-	0.25	none	started
reicher	psychology	-	-	0.24	none	started
member	psychology	-	-	0.18	none	started
st_andrews	psychology	-	-	0.31	none	started
