# Supplementary material for: Lessons From the UK's Lockdown: Discourse on Behavioural Science in Times of COVID-19
Source: Front Psychol. 2021 Jun 17;12:647348. doi: 10.3389/fpsyg.2021.647348 (PMC8247580; doi:10.3389/fpsyg.2021.647348)
Supplement: Supplementary file 7 [file Data_Sheet_7.PDF]

Supplementary Material 7

Supplementary Material 7: Sentiments towards keywords separated by public policy application mentions for newspaper articles (Study 1).

						Count of sentiments (per fortnight)			Proportion of sentiments by polarity (per fortnight)			Proportion of sentiments by polarity (per time window)			Proportion of sentiments by polarity (per policy presence)		
						neg	neu	pos	neg	neu	pos	neg	neu	pos	neg	neu	pos
**Keyword**	**Policy application is mentioned**	**Time window**	**Fortnight starting**	**Fortnight to lockdown**	**Tot counts Of sentiments**												
Behavioural change (behaviour change)	no	before-lockdown	2020-01-27	-4	0	0	0	0				0	0.75	0.25	0	0.97	0.03
			2020-02-10	-3	0	0	0	0									
			2020-02-24	-2	0	0	0	0									
			2020-03-09	-1	4	0	3	1	0	0.75	0.25						
		lockdown	2020-03-23	0	1	0	1	0	0	1	0	0	1	0			
			2020-04-06	1	2	0	2	0	0	1	0						
			2020-04-20	2	5	0	5	0	0	1	0						
			2020-05-04	3	8	0	8	0	0	1	0						
		post-lockdown	2020-05-18	4	3	0	3	0	0	1	0	0	1	0			
			2020-06-01	5	1	0	1	0	0	1	0						
			2020-06-15	6	1	0	1	0	0	1	0						
			2020-06-29	7	4	0	4	0	0	1	0						
	yes	before-lockdown	2020-01-27	-4	0	0	0	0				0	1	0	0	0.94	0.06
			2020-02-10	-3	0	0	0	0									
			2020-02-24	-2	0	0	0	0									
			2020-03-09	-1	1	0	1	0	0	1	0						
		lockdown	2020-03-23	0	10	0	9	1	0	0.9	0.1	0	0.92	0.08			
			2020-04-06	1	1	0	1	0	0	1	0						
			2020-04-20	2	1	0	1	0	0	1	0						
			2020-05-04	3	0	0	0	0									
		post-lockdown	2020-05-18	4	0	0	0	0				0	1	0			
			2020-06-01	5	3	0	3	0	0	1	0						
			2020-06-15	6	0	0	0	0									
			2020-06-29	7	0	0	0	0									
behavioural economics (behavioural economists)	no	before-lockdown	2020-01-27	-4	1	1	0	0	1	0	0	0.29	0.57	0.14	0.21	0.65	0.15
			2020-02-10	-3	2	0	1	1	0	0.5	0.5						
			2020-02-24	-2	1	1	0	0	1	0	0						
			2020-03-09	-1	3	0	3	0	0	1	0						
		lockdown	2020-03-23	0	6	1	4	1	0.17	0.67	0.17	0.1	0.75	0.15			
			2020-04-06	1	6	0	6	0	0	1	0						
			2020-04-20	2	5	1	4	0	0.2	0.8	0						
			2020-05-04	3	3	0	1	2	0	0.33	0.67						
		post-lockdown	2020-05-18	4	5	2	2	1	0.4	0.4	0.2	0.43	0.43	0.14			
			2020-06-01	5	0	0	0	0									
			2020-06-15	6	1	1	0	0	1	0	0						
			2020-06-29	7	1	0	1	0	0	1	0						
	yes	before-lockdown	2020-01-27	-4	0	0	0	0							0	1	0
			2020-02-10	-3	0	0	0	0									
			2020-02-24	-2	0	0	0	0									
			2020-03-09	-1	0	0	0	0									
		lockdown	2020-03-23	0	0	0	0	0				0	1	0			
			2020-04-06	1	0	0	0	0									
			2020-04-20	2	0	0	0	0									
			2020-05-04	3	1	0	1	0	0	1	0						
		post-lockdown	2020-05-18	4	0	0	0	0				0	1	0			
			2020-06-01	5	0	0	0	0									
			2020-06-15	6	0	0	0	0									
			2020-06-29	7	1	0	1	0	0	1	0						
		before-lockdown	2020-01-27	-4	0	0	0	0				0.14	0.57	0.29			
			2020-02-10	-3	0	0	0	0									
			2020-02-24	-2	2	0	2	0	0	1	0						
			2020-03-09	-1	12	2	6	4	0.17	0.5	0.33						
			2020-03-23	0	8	0	5	3	0	0.62	0.38						

Supplementary Material 7: Sentiments towards keywords separated by public policy application mentions for newspaper articles (Study 1).

						Count of sentiments (per fortnight)			Proportion of sentiments by polarity (per fortnight)			Proportion of sentiments by polarity (per time window)			Proportion of sentiments by polarity (per policy presence)		
						neg	neu	pos	neg	neu	pos	neg	neu	pos	neg	neu	pos
Behavioural Insights Team (nudge unit)	no	lockdown	2020-04-06	1	2	2	0	0	1	0	0				0.14	0.6	0.26
			2020-04-20	2	6	1	5	0	0.17	0.83	0						
			2020-05-04	3	7	1	3	3	0.14	0.43	0.43	0.17	0.57	0.26			
		post-lockdown	2020-05-18	4	1	0	1	0	0	1	0						
			2020-06-01	5	0	0	0	0									
			2020-06-15	6	4	0	3	1	0	0.75	0.25						
			2020-06-29	7	0	0	0	0				0	0.8	0.2			
	yes	before-lockdown	2020-01-27	-4	2	0	1	1	0	0.5	0.5				0.23	0.63	0.14
			2020-02-10	-3	3	1	0	2	0.33	0	0.67						
			2020-02-24	-2	8	2	6	0	0.25	0.75	0						
			2020-03-09	-1	40	13	23	4	0.32	0.57	0.1	0.3	0.57	0.13			
		lockdown	2020-03-23	0	11	1	4	6	0.09	0.36	0.55						
			2020-04-06	1	4	1	3	0	0.25	0.75	0						
			2020-04-20	2	10	2	8	0	0.2	0.8	0						
			2020-05-04	3	6	0	6	0	0	1	0	0.13	0.68	0.19			
		post-lockdown	2020-05-18	4	3	0	3	0	0	1	0						
			2020-06-01	5	1	1	0	0	1	0	0						
			2020-06-15	6	4	0	4	0	0	1	0						
			2020-06-29	7	0	0	0	0				0.12	0.88	0			
Behavioural Science (behavioural sciences, behavioural policy, behavioural scientists)	no	before-lockdown	2020-01-27	-4	0	0	0	0				0.24	0.47	0.29	0.15	0.62	0.22
			2020-02-10	-3	3	0	2	1	0	0.67	0.33						
			2020-02-24	-2	13	2	5	6	0.15	0.38	0.46						
			2020-03-09	-1	43	12	21	10	0.28	0.49	0.23						
		lockdown	2020-03-23	0	26	12	11	3	0.46	0.42	0.12	0.16	0.65	0.19			
			2020-04-06	1	28	1	19	8	0.04	0.68	0.29						
			2020-04-20	2	34	5	25	4	0.15	0.74	0.12						
			2020-05-04	3	36	2	25	9	0.06	0.69	0.25						
		post-lockdown	2020-05-18	4	26	0	18	8	0	0.69	0.31	0.09	0.68	0.23			
			2020-06-01	5	24	2	17	5	0.08	0.71	0.21						
			2020-06-15	6	34	6	22	6	0.18	0.65	0.18						
			2020-06-29	7	13	1	9	3	0.08	0.69	0.23						
	yes	before-lockdown	2020-01-27	-4	0	0	0	0				0.37	0.4	0.23	0.22	0.52	0.26
			2020-02-10	-3	0	0	0	0									
			2020-02-24	-2	3	1	1	1	0.33	0.33	0.33						
			2020-03-09	-1	27	10	11	6	0.37	0.41	0.22						
		lockdown	2020-03-23	0	20	5	6	9	0.25	0.3	0.45	0.19	0.54	0.27			
			2020-04-06	1	23	4	14	5	0.17	0.61	0.22						
			2020-04-20	2	13	2	10	1	0.15	0.77	0.08						
			2020-05-04	3	3	0	2	1	0	0.67	0.33						
		post-lockdown	2020-05-18	4	28	2	23	3	0.07	0.82	0.11	0.18	0.56	0.25			
			2020-06-01	5	2	0	2	0	0	1	0						
			2020-06-15	6	22	8	3	11	0.36	0.14	0.5						
			2020-06-29	7	3	0	3	0	0	1	0						
Behavioural Scientist	no	before-lockdown	2020-01-27	-4	3	0	3	0	0	1	0				0.07	0.86	0.07
			2020-02-10	-3	1	0	1	0	0	1	0						
			2020-02-24	-2	1	0	1	0	0	1	0						
			2020-03-09	-1	3	0	3	0	0	1	0	0	1	0			
		lockdown	2020-03-23	0	4	1	2	1	0.25	0.5	0.25						
			2020-04-06	1	7	0	7	0	0	1	0						
			2020-04-20	2	7	0	7	0	0	1	0						
			2020-05-04	3	7	2	5	0	0.29	0.71	0	0.12	0.84	0.04			
		post-lockdown	2020-05-18	4	5	0	4	1	0	0.8	0.2						
			2020-06-01	5	1	0	0	1	0	0	1						
			2020-06-15	6	1	0	1	0	0	1	0						
			2020-06-29	7	4	0	4	0	0	1	0	0	0.82	0.18			

Supplementary Material 7: Sentiments towards keywords separated by public policy application mentions for newspaper articles (Study 1).

						Count of sentiments (per fortnight)			Proportion of sentiments by polarity (per fortnight)			Proportion of sentiments by polarity (per time window)			Proportion of sentiments by polarity (per policy presence)		
						neg	neu	pos	neg	neu	pos	neg	neu	pos	neg	neu	pos
Behavioural Scientist			2020-01-27	-4	0	0	0	0									
		before-lockdown	2020-02-10	-3	0	0	0	0									
			2020-02-24	-2	0	0	0	0									
			2020-03-09	-1	4	0	1	3	0	0.25	0.75	0	0.25	0.75			
			2020-03-23	0	3	0	3	0	0	1	0						
	yes	lockdown	2020-04-06	1	2	0	1	1	0	0.5	0.5				0.04	0.75	0.21
			2020-04-20	2	1	0	1	0	0	1	0						
			2020-05-04	3	3	0	3	0	0	1	0	0	0.89	0.11			
			2020-05-18	4	3	0	3	0	0	1	0						
		post-lockdown	2020-06-01	5	1	0	1	0	0	1	0						
			2020-06-15	6	6	1	4	1	0.17	0.67	0.17						
			2020-06-29	7	1	0	1	0	0	1	0	0.09	0.82	0.09			
			2020-01-27	-4	0	0	0	0									
		before-lockdown	2020-02-10	-3	0	0	0	0									
			2020-02-24	-2	0	0	0	0									
			2020-03-09	-1	9	0	9	0	0	1	0	0	1	0			
			2020-03-23	0	5	0	5	0	0	1	0						
	no	lockdown	2020-04-06	1	1	0	1	0	0	1	0				0.22	0.66	0.12
			2020-04-20	2	6	3	2	1	0.5	0.33	0.17						
			2020-05-04	3	3	0	3	0	0	1	0	0.2	0.73	0.07			
			2020-05-18	4	1	1	0	0	1	0	0						
		post-lockdown	2020-06-01	5	0	0	0	0									
			2020-06-15	6	7	3	1	3	0.43	0.14	0.43						
			2020-06-29	7	0	0	0	0				0.5	0.12	0.38			
Halpern			2020-01-27	-4	0	0	0	0									
		before-lockdown	2020-02-10	-3	0	0	0	0									
			2020-02-24	-2	0	0	0	0									
			2020-03-09	-1	27	10	15	2	0.37	0.56	0.07	0.37	0.56	0.07			
			2020-03-23	0	11	4	2	5	0.36	0.18	0.45						
	yes	lockdown	2020-04-06	1	2	0	2	0	0	1	0				0.31	0.55	0.14
			2020-04-20	2	4	1	3	0	0.25	0.75	0						
			2020-05-04	3	4	0	4	0	0	1	0	0.24	0.52	0.24			
			2020-05-18	4	2	0	2	0	0	1	0						
		post-lockdown	2020-06-01	5	0	0	0	0									
			2020-06-15	6	1	1	0	0	1	0	0						
			2020-06-29	7	0	0	0	0				0.33	0.67	0			
			2020-01-27	-4	0	0	0	0									
		before-lockdown	2020-02-10	-3	0	0	0	0									
			2020-02-24	-2	0	0	0	0									
			2020-03-09	-1	23	2	21	0	0.09	0.91	0	0.09	0.91	0			
			2020-03-23	0	5	0	4	1	0	0.8	0.2						
	no	lockdown	2020-04-06	1	13	0	13	0	0	1	0				0.02	0.97	0.01
			2020-04-20	2	19	0	19	0	0	1	0						
			2020-05-04	3	18	1	17	0	0.06	0.94	0	0.02	0.96	0.02			
			2020-05-18	4	9	0	9	0	0	1	0						
		post-lockdown	2020-06-01	5	9	0	9	0	0	1	0						
			2020-06-15	6	17	0	17	0	0	1	0						
			2020-06-29	7	10	0	10	0	0	1	0	0	1	0			
Michie			2020-01-27	-4	0	0	0	0									
		before-lockdown	2020-02-10	-3	0	0	0	0									
			2020-02-24	-2	0	0	0	0									
			2020-03-09	-1	21	0	8	13	0	0.38	0.62	0	0.38	0.62			
			2020-03-23	0	11	0	11	0	0	1	0						
	yes	lockdown	2020-04-06	1	3	0	2	1	0	0.67	0.33				0.01	0.77	0.22
			2020-04-20	2	12	0	10	2	0	0.83	0.17						

Supplementary Material 7: Sentiments towards keywords separated by public policy application mentions for newspaper articles (Study 1).

						Count of sentiments (per fortnight)			Proportion of sentiments by polarity (per fortnight)			Proportion of sentiments by polarity (per time window)			Proportion of sentiments by polarity (per policy presence)		
						neg	neu	pos	neg	neu	pos	neg	neu	pos	neg	neu	pos
			2020-05-04	3	10	1	9	0	0.1	0.9	0	0.03	0.89	0.08			
			2020-05-18	4	9	0	8	1	0	0.89	0.11						
		post-lockdown	2020-06-01	5	14	0	13	1	0	0.93	0.07						
			2020-06-15	6	0	0	0	0									
			2020-06-29	7	3	0	3	0	0	1	0	0	0.92	0.08			
			2020-01-27	-4	5	1	0	4	0.2	0	0.8						
		before-lockdown	2020-02-10	-3	6	3	1	2	0.5	0.17	0.33	0.41	0.22	0.37			
			2020-02-24	-2	2	1	1	0	0.5	0.5	0						
			2020-03-09	-1	14	6	4	4	0.43	0.29	0.29						
			2020-03-23	0	11	8	0	3	0.73	0	0.27						
	no	lockdown	2020-04-06	1	12	5	1	6	0.42	0.08	0.5	0.58	0.16	0.26	0.46	0.21	0.32
			2020-04-20	2	14	8	5	1	0.57	0.36	0.07						
			2020-05-04	3	1	1	0	0	1	0	0						
			2020-05-18	4	8	1	3	4	0.12	0.38	0.5						
		post-lockdown	2020-06-01	5	5	3	0	2	0.6	0	0.4	0.27	0.33	0.4			
			2020-06-15	6	1	0	1	0	0	1	0						
Nudge (nudges, nudging, nudge theory, nudge strategy, paternalism, libertarian paternalism, paternalistic)			2020-06-29	7	1	0	1	0	0	1	0						
			2020-01-27	-4	2	0	0	2	0	0	1						
		before-lockdown	2020-02-10	-3	3	0	1	2	0	0.33	0.67						
			2020-02-24	-2	1	1	0	0	1	0	0						
			2020-03-09	-1	11	4	4	3	0.36	0.36	0.27	0.29	0.29	0.41			
			2020-03-23	0	4	2	0	2	0.5	0	0.5						
	yes	lockdown	2020-04-06	1	4	2	2	0	0.5	0.5	0				0.39	0.27	0.33
			2020-04-20	2	4	1	1	2	0.25	0.25	0.5						
			2020-05-04	3	1	1	0	0	1	0	0	0.46	0.23	0.31			
			2020-05-18	4	0	0	0	0									
		post-lockdown	2020-06-01	5	1	1	0	0	1	0	0						
			2020-06-15	6	2	1	1	0	0.5	0.5	0						
			2020-06-29	7	0	0	0	0				0.67	0.33	0			
			2020-01-27	-4	0	0	0	0									
		before-lockdown	2020-02-10	-3	2	0	1	1	0	0.5	0.5						
			2020-02-24	-2	4	0	3	1	0	0.75	0.25						
			2020-03-09	-1	8	0	8	0	0	1	0	0	0.86	0.14			
			2020-03-23	0	6	0	5	1	0	0.83	0.17						
	no	lockdown	2020-04-06	1	7	0	6	1	0	0.86	0.14				0	0.9	0.1
			2020-04-20	2	7	0	6	1	0	0.86	0.14						
			2020-05-04	3	7	0	7	0	0	1	0	0	0.89	0.11			
			2020-05-18	4	7	0	7	0	0	1	0						
		post-lockdown	2020-06-01	5	0	0	0	0									
			2020-06-15	6	7	0	6	1	0	0.86	0.14						
Psychologist			2020-06-29	7	5	0	5	0	0	1	0	0	0.95	0.05			
			2020-01-27	-4	0	0	0	0									
		before-lockdown	2020-02-10	-3	0	0	0	0									
			2020-02-24	-2	0	0	0	0									
			2020-03-09	-1	0	0	0	0									
			2020-03-23	0	0	0	0	0									
	yes	lockdown	2020-04-06	1	1	0	1	0	0	1	0				0	1	0
			2020-04-20	2	3	0	3	0	0	1	0						
			2020-05-04	3	1	0	1	0	0	1	0	0	1	0			
			2020-05-18	4	1	0	1	0	0	1	0						
		post-lockdown	2020-06-01	5	4	0	4	0	0	1	0						
			2020-06-15	6	0	0	0	0									
			2020-06-29	7	2	0	2	0	0	1	0	0	1	0			
			2020-01-27	-4	1	0	1	0	0	1	0						
		before-lockdown	2020-02-10	-3	0	0	0	0									

Supplementary Material 7: Sentiments towards keywords separated by public policy application mentions for newspaper articles (Study 1).

						Count of sentiments (per fortnight)			Proportion of sentiments by polarity (per fortnight)			Proportion of sentiments by polarity (per time window)			Proportion of sentiments by polarity (per policy presence)		
						neg	neu	pos	neg	neu	pos	neg	neu	pos	neg	neu	pos
Psychology (psychologists, psychological science, psychological policy)	no	before-lockdown	2020-02-24	-2	2	0	2	0	0	1	0				0.04	0.82	0.14
			2020-03-09	-1	18	0	16	2	0	0.89	0.11	0	0.9	0.1			
		lockdown	2020-03-23	0	10	1	5	4	0.1	0.5	0.4						
			2020-04-06	1	8	0	6	2	0	0.75	0.25						
			2020-04-20	2	20	3	17	0	0.15	0.85	0						
			2020-05-04	3	18	1	16	1	0.06	0.89	0.06	0.09	0.79	0.12			
		post-lockdown	2020-05-18	4	17	0	14	3	0	0.82	0.18						
			2020-06-01	5	10	0	10	0	0	1	0						
			2020-06-15	6	17	0	12	5	0	0.71	0.29						
			2020-06-29	7	4	0	4	0	0	1	0	0	0.83	0.17			
	yes	before-lockdown	2020-01-27	-4	0	0	0	0							0.05	0.72	0.22
			2020-02-10	-3	0	0	0	0									
			2020-02-24	-2	0	0	0	0									
			2020-03-09	-1	12	0	7	5	0	0.58	0.42	0	0.58	0.42			
		lockdown	2020-03-23	0	1	0	0	1	0	0	1						
			2020-04-06	1	6	1	5	0	0.17	0.83	0						
			2020-04-20	2	2	0	2	0	0	1	0						
			2020-05-04	3	6	1	4	1	0.17	0.67	0.17	0.13	0.73	0.13			
		post-lockdown	2020-05-18	4	12	0	10	2	0	0.83	0.17						
			2020-06-01	5	0	0	0	0									
			2020-06-15	6	0	0	0	0									
			2020-06-29	7	1	0	1	0	0	1	0	0	0.85	0.15			
Spi-b	no	before-lockdown	2020-01-27	-4	0	0	0	0							0.04	0.84	0.11
			2020-02-10	-3	0	0	0	0									
			2020-02-24	-2	0	0	0	0									
			2020-03-09	-1	3	0	3	0	0	1	0	0	1	0			
		lockdown	2020-03-23	0	1	1	0	0	1	0	0						
			2020-04-06	1	1	0	1	0	0	1	0						
			2020-04-20	2	10	0	10	0	0	1	0						
			2020-05-04	3	49	4	39	6	0.08	0.8	0.12	0.08	0.82	0.1			
		post-lockdown	2020-05-18	4	30	0	25	5	0	0.83	0.17						
			2020-06-01	5	12	0	10	2	0	0.83	0.17						
			2020-06-15	6	6	0	6	0	0	1	0						
			2020-06-29	7	4	0	4	0	0	1	0	0	0.87	0.13			
	yes	before-lockdown	2020-01-27	-4	0	0	0	0							0	0.9	0.1
			2020-02-10	-3	0	0	0	0									
			2020-02-24	-2	0	0	0	0									
			2020-03-09	-1	1	0	1	0	0	1	0	0	1	0			
		lockdown	2020-03-23	0	1	0	0	1	0	0	1						
			2020-04-06	1	2	0	1	1	0	0.5	0.5						
			2020-04-20	2	6	0	6	0	0	1	0						
			2020-05-04	3	21	0	19	2	0	0.9	0.1	0	0.87	0.13			
		post-lockdown	2020-05-18	4	13	0	12	1	0	0.92	0.08						
			2020-06-01	5	0	0	0	0									
			2020-06-15	6	1	0	1	0	0	1	0						
			2020-06-29	7	5	0	5	0	0	1	0	0	0.95	0.05			
