# Supplementary material for: Lessons From the UK's Lockdown: Discourse on Behavioural Science in Times of COVID-19
Source: Front Psychol. 2021 Jun 17;12:647348. doi: 10.3389/fpsyg.2021.647348 (PMC8247580; doi:10.3389/fpsyg.2021.647348)
Supplement: Supplementary file 8 [file Data_Sheet_8.PDF]

Supplementary Material 8: Sentiments towards public policy actors mentioned alongside behavioural science keywords for newspaper articles (Study 1).

					Count of sentiments (per fortnight)			Proportion of sentiments by polarity (per fortnight)			Proportion of sentiments by polarity (time window)			Proportion of sentiments (overall)		
					neg	neu	pos	neg	neu	pos	neg	neu	pos	neg	neu	pos
**Keyword**	**Time window**	**Fortnight to lockdown**	**Fortnight starting**	**Total N of sentiments**												
Behavior change	before-lockdown	-4	2020-01-27	0	0	0	0							0.38	0.62	0
		-3	2020-02-10	0	0	0	0									
		-2	2020-02-24	0	0	0	0									
		-1	2020-03-09	1	0	1	0	0	1	0	0	1	0			
	lockdown	0	2020-03-23	10	3	7	0	0.3	0.7	0						
		1	2020-04-06	1	0	1	0	0	1	0						
		2	2020-04-20	1	0	1	0	0	1	0						
		3	2020-05-04	0	0	0	0				0.25	0.75	0			
	post-lockdown	4	2020-05-18	0	0	0	0									
		5	2020-06-01	3	3	0	0	1	0	0						
		6	2020-06-15	0	0	0	0									
		7	2020-06-29	0	0	0	0				1	0	0			
Behavioural economics (behavioural economists)	before-lockdown	-4	2020-01-27	0	0	0	0							0.5	0	0.5
		-3	2020-02-10	0	0	0	0									
		-2	2020-02-24	0	0	0	0									
		-1	2020-03-09	0	0	0	0									
	lockdown	0	2020-03-23	0	0	0	0									
		1	2020-04-06	0	0	0	0									
		2	2020-04-20	0	0	0	0									
		3	2020-05-04	1	0	0	1	0	0	1	0	0	1			
	post-lockdown	4	2020-05-18	0	0	0	0									
		5	2020-06-01	0	0	0	0									
		6	2020-06-15	0	0	0	0									
		7	2020-06-29	1	1	0	0	1	0	0	1	0	0			
Behavioural Insights Team (nudge unit)	before-lockdown	-4	2020-01-27	2	0	2	0	0	1	0				0.27	0.63	0.1
		-3	2020-02-10	3	1	0	2	0.33	0	0.67						
		-2	2020-02-24	8	8	0	0	1	0	0						
		-1	2020-03-09	40	11	28	1	0.28	0.7	0.02	0.38	0.57	0.06			
	lockdown	0	2020-03-23	11	0	6	5	0	0.55	0.45						
		1	2020-04-06	3	0	3	0	0	1	0						
		2	2020-04-20	5	2	3	0	0.4	0.6	0						
		3	2020-05-04	5	0	5	0	0	1	0	0.08	0.71	0.21			
	post-lockdown	4	2020-05-18	2	0	2	0	0	1	0						
		5	2020-06-01	1	1	0	0	1	0	0						
		6	2020-06-15	4	0	4	0	0	1	0						
		7	2020-06-29	0	0	0	0				0.14	0.86	0			
		-4	2020-01-27	0	0	0	0									

		-3	2020-02-10	0	0	0	0									
		-2	2020-02-24	3	0	3	0	0	1	0	0.37	0.6	0.03			
	before-lockdown	-1	2020-03-09	27	11	15	1	0.41	0.56	0.04						
Behavioural science (behavioural sciences, behavioural policy, behavioural scientists)		0	2020-03-23	20	7	8	5	0.35	0.4	0.25						
		1	2020-04-06	19	12	3	4	0.63	0.16	0.21	0.42	0.4	0.19	0.57	0.34	0.09
		2	2020-04-20	11	2	8	1	0.18	0.73	0.09						
	lockdown	3	2020-05-04	3	1	2	0	0.33	0.67	0						
		4	2020-05-18	28	24	4	0	0.86	0.14	0						
		5	2020-06-01	2	1	0	1	0.5	0	0.5	0.82	0.15	0.04			
		6	2020-06-15	22	19	2	1	0.86	0.09	0.05						
	post-lockdown	7	2020-06-29	3	1	2	0	0.33	0.67	0						
		-4	2020-01-27	0	0	0	0									
	before-lockdown	-3	2020-02-10	0	0	0	0									
		-2	2020-02-24	0	0	0	0									
		-1	2020-03-09	4	4	0	0	1	0	0	1	0	0			
		0	2020-03-23	3	3	0	0	1	0	0						
Behavioural scientist	lockdown	1	2020-04-06	2	2	0	0	1	0	0				0.83	0.17	0
		2	2020-04-20	1	0	1	0	0	1	0						
		3	2020-05-04	3	1	2	0	0.33	0.67	0	0.67	0.33	0			
		4	2020-05-18	3	3	0	0	1	0	0						
	post-lockdown	5	2020-06-01	1	1	0	0	1	0	0						
		6	2020-06-15	6	6	0	0	1	0	0						
		7	2020-06-29	1	0	1	0	0	1	0	0.91	0.09	0			
		-4	2020-01-27	0	0	0	0									
	before-lockdown	-3	2020-02-10	0	0	0	0									
		-2	2020-02-24	0	0	0	0									
		-1	2020-03-09	27	3	24	0	0.11	0.89	0	0.11	0.89	0			
		0	2020-03-23	11	4	5	2	0.36	0.45	0.18						
Halpern	lockdown	1	2020-04-06	2	0	2	0	0	1	0						
		2	2020-04-20	3	1	2	0	0.33	0.67	0						
		3	2020-05-04	3	0	3	0	0	1	0	0.26	0.63	0.11			
		4	2020-05-18	1	0	1	0	0	1	0						
	post-lockdown	5	2020-06-01	0	0	0	0									
		6	2020-06-15	2	0	1	1	0	0.5	0.5						
		7	2020-06-29	0	0	0	0				0	0.67	0.33	0.16	0.78	0.06
		-4	2020-01-27	0	0	0	0									
	before-lockdown	-3	2020-02-10	0	0	0	0									
		-2	2020-02-24	0	0	0	0									
		-1	2020-03-09	21	11	10	0	0.52	0.48	0	0.52	0.48	0			
		0	2020-03-23	11	6	5	0	0.55	0.45	0						
Michie	lockdown	1	2020-04-06	1	0	0	1	0	0	1				0.67	0.32	0.01
		2	2020-04-20	12	5	7	0	0.42	0.58	0						
		3	2020-05-04	10	7	3	0	0.7	0.3	0	0.53	0.44	0.03			
		4	2020-05-18	9	9	0	0	1	0	0						
	post-lockdown	5	2020-06-01	14	13	1	0	0.93	0.07	0						
		6	2020-06-15	0	0	0	0									
		7	2020-06-29	3	3	0	0	1	0	0	0.96	0.04	0			
		-4	2020-01-27	2	2	0	0	1	0	0						

Nudge<br>(nudges, nudging,<br>nudge theory,<br>nudge strategy,<br>paternalism,<br>libertarian paternalism,<br>paternalistic)	before-lockdown	-3	2020-02-10	3	2	0	1	0.67	0	0.33						
		-2	2020-02-24	1	0	1	0	0	1	0						
		-1	2020-03-09	11	4	7	0	0.36	0.64	0	0.47	0.47	0.06			
	lockdown	0	2020-03-23	4	3	1	0	0.75	0.25	0						
		1	2020-04-06	4	1	3	0	0.25	0.75	0				0.5	0.47	0.03
		2	2020-04-20	3	0	3	0	0	1	0						
		3	2020-05-04	1	1	0	0	1	0	0	0.42	0.58	0			
	post-lockdown	4	2020-05-18	0	0	0	0									
		5	2020-06-01	1	1	0	0	1	0	0						
		6	2020-06-15	2	2	0	0	1	0	0						
		7	2020-06-29	0	0	0	0				1	0	0			
Psychologist	before-lockdown	-4	2020-01-27	0	0	0	0									
		-3	2020-02-10	0	0	0	0									
		-2	2020-02-24	0	0	0	0									
		-1	2020-03-09	0	0	0	0									
	lockdown	0	2020-03-23	0	0	0	0									
		1	2020-04-06	1	0	1	0	0	1	0				0.83	0.17	0
		2	2020-04-20	3	2	1	0	0.67	0.33	0						
		3	2020-05-04	1	1	0	0	1	0	0	0.6	0.4	0			
	post-lockdown	4	2020-05-18	1	1	0	0	1	0	0						
		5	2020-06-01	4	4	0	0	1	0	0						
		6	2020-06-15	0	0	0	0									
		7	2020-06-29	2	2	0	0	1	0	0	1	0	0			
Psychology<br>(psychologists,<br>psychological science,<br>psychological policy)	before-lockdown	-4	2020-01-27	0	0	0	0									
		-3	2020-02-10	0	0	0	0									
		-2	2020-02-24	0	0	0	0									
		-1	2020-03-09	12	4	8	0	0.33	0.67	0	0.33	0.67	0			
	lockdown	0	2020-03-23	1	0	1	0	0	1	0						
		1	2020-04-06	3	1	2	0	0.33	0.67	0				0.59	0.38	0.03
		2	2020-04-20	2	0	1	1	0	0.5	0.5						
		3	2020-05-04	6	5	1	0	0.83	0.17	0	0.5	0.42	0.08			
	post-lockdown	4	2020-05-18	12	11	1	0	0.92	0.08	0						
		5	2020-06-01	0	0	0	0									
		6	2020-06-15	0	0	0	0									
		7	2020-06-29	1	1	0	0	1	0	0	0.92	0.08	0			
SPI-B	before-lockdown	-4	2020-01-27	0	0	0	0									
		-3	2020-02-10	0	0	0	0									
		-2	2020-02-24	0	0	0	0									
		-1	2020-03-09	1	0	1	0	0	1	0	0	1	0			
	lockdown	0	2020-03-23	1	0	0	1	0	0	1						
		1	2020-04-06	2	2	0	0	1	0	0				0.64	0.28	0.08
		2	2020-04-20	6	2	4	0	0.33	0.67	0						
		3	2020-05-04	21	15	6	0	0.71	0.29	0	0.63	0.33	0.03			
	post-lockdown	4	2020-05-18	13	11	2	0	0.85	0.15	0						
		5	2020-06-01	0	0	0	0									
		6	2020-06-15	1	1	0	0	1	0	0						
		7	2020-06-29	5	1	1	3	0.2	0.2	0.6	0.68	0.16	0.16			
