# Supplementary material for: Lessons From the UK's Lockdown: Discourse on Behavioural Science in Times of COVID-19
Source: Front Psychol. 2021 Jun 17;12:647348. doi: 10.3389/fpsyg.2021.647348 (PMC8247580; doi:10.3389/fpsyg.2021.647348)
Supplement: Supplementary file 9 [file Data_Sheet_9.PDF]

Supplementary Material 9: Trends in salience and sentiment for keywords per fortnight in Twitter data (Study 2).

				Salience		Total N		Count of sentiments			Proportion of sentiments			Count of sentiments			Proportion of sentiments		
				Original tweets only	Incl. Retweets	Original tweets	Incl. Retweets	Original tweets only			Original tweets only			Incl. Retweets			Incl. Retweets		
Keyword	Fortnight starting	Fortnight to lockdown	Time window					Neu	Pos	Neg	Neu	Pos	Neg	Neu	Pos	Neg	Neu	Neg	Pos
Behaviour change	2020-02-24	-2	before-lockdown	0.30	0.13	23	43	12	10	1	0.52	0.43	0.04	28	14	1	0.65	0.02	0.33
	2020-03-09	-1		0.17	0.07	93	328	40	49	4	0.43	0.53	0.04	125	198	5	0.38	0.02	0.6
	2020-03-23	0	lockdown	0.32	0.11	92	264	49	41	2	0.53	0.45	0.02	148	110	6	0.56	0.02	0.42
	2020-04-06	1		0.48	0.46	147	535	92	53	2	0.63	0.36	0.01	190	343	2	0.36	0	0.64
	2020-04-20	2		0.41	0.50	110	542	54	54	2	0.49	0.49	0.02	148	382	12	0.27	0.02	0.7
	2020-05-04	3		0.35	0.36	122	578	57	65		0.47	0.53		336	242		0.58		0.42
	2020-05-18	4	post-lockdown	0.38	0.22	78	337	40	35	3	0.51	0.45	0.04	111	222	4	0.33	0.01	0.66
	2020-06-01	5		0.32	0.26	44	116	18	25	1	0.41	0.57	0.02	49	65	2	0.42	0.02	0.56
Behavioural economics (behavioural economists)	2020-02-24	-2	before-lockdown	0.04	0.01	3	3	1	1	1	0.33	0.33	0.33	1	1	1	0.33	0.33	0.33
	2020-03-09	-1		0.03	0.02	15	81	8	4	3	0.53	0.27	0.2	16	29	36	0.2	0.44	0.36
	2020-03-23	0	lockdown	0.03	0.01	9	25	6	3		0.67	0.33		17	8		0.68		0.32
	2020-04-06	1		0.03	0.02	8	18	7	1		0.88	0.12		16	2		0.89		0.11
	2020-04-20	2		0.06	0.03	16	34	7	5	4	0.44	0.31	0.25	16	5	13	0.47	0.38	0.15
	2020-05-04	3		0.02	0.01	8	23	6		2	0.75		0.25	21		2	0.91	0.09	
	2020-05-18	4	post-lockdown	0.01	0.01	3	8	2	1		0.67	0.33		5	3		0.62		0.38
	2020-06-01	5		0.04	0.01	5	6	4	1		0.8	0.2		5	1		0.83		0.17
Behavioural Insights Team (nudge unit)	2020-02-24	-2	before-lockdown	0.01	0.00	1	1	1			1			1			1		
	2020-03-09	-1		0.16	0.16	89	765	34	5	50	0.38	0.06	0.56	250	33	482	0.33	0.63	0.04
	2020-03-23	0	lockdown	0.13	0.29	37	677	11	3	23	0.3	0.08	0.62	70	12	595	0.1	0.88	0.02
	2020-04-06	1		0.10	0.12	32	136	2	4	26	0.06	0.12	0.81	25	5	106	0.18	0.78	0.04
	2020-04-20	2		0.04	0.09	12	94	8		4	0.67		0.33	15		79	0.16	0.84	
	2020-05-04	3		0.10	0.16	34	256	16	2	16	0.47	0.06	0.47	102	17	137	0.4	0.54	0.07
	2020-05-18	4	post-lockdown	0.03	0.05	7	80	2	1	4	0.29	0.14	0.57	4	3	73	0.05	0.91	0.04
	2020-06-01	5		0.06	0.04	8	17	2		6	0.25		0.75	4		13	0.24	0.76	
Behavioural science (behavioural sciences, behavioural policy, behavioural scientists)	2020-02-24	-2	before-lockdown	0.29	0.36	22	120	10	12		0.45	0.55		65	55		0.54		0.46
	2020-03-09	-1		0.28	0.15	157	751	58	43	56	0.37	0.27	0.36	393	122	236	0.52	0.31	0.16
	2020-03-23	0	lockdown	0.29	0.43	84	990	40	32	12	0.48	0.38	0.14	736	216	38	0.74	0.04	0.22
	2020-04-06	1		0.25	0.27	77	317	28	41	8	0.36	0.53	0.1	68	218	31	0.21	0.1	0.69
	2020-04-20	2		0.27	0.20	73	217	24	40	9	0.33	0.55	0.12	71	84	62	0.33	0.29	0.39
	2020-05-04	3		0.26	0.20	91	325	31	42	18	0.34	0.46	0.2	102	117	106	0.31	0.33	0.36
	2020-05-18	4	post-lockdown	0.33	0.13	68	206	53	14	1	0.78	0.21	0.01	159	43	4	0.77	0.02	0.21
	2020-06-01	5		0.26	0.36	36	160	11	23	2	0.31	0.64	0.06	32	123	5	0.2	0.03	0.77
Behavioural scientist	2020-02-24	-2	before-lockdown	0.01	0.00	1	1			1			1			1		1	
	2020-03-09	-1		0.05	0.07	27	340	11		16	0.41		0.59	228		112	0.67	0.33	
	2020-03-23	0	lockdown	0.03	0.04	8	100	3	1	4	0.38	0.12	0.5	68	1	31	0.68	0.31	0.01
	2020-04-06	1		0.01	0.02	4	18	4			1			18			1		
	2020-04-20	2		0.01	0.01	2	11	1		1	0.5		0.5	1		10	0.09	0.91	
	2020-05-04	3		0.01	0.00	4	7	2		2	0.5		0.5	4		3	0.57	0.43	
	2020-05-18	4	post-lockdown	0.02	0.02	4	31	3		1	0.75		0.25	30		1	0.97	0.03	
	2020-06-01	5		0.01	0.09	1	39	1			1			39			1		
Halpern	2020-02-24	-2	before-lockdown	0.01	0.00	1	1		1			1			1				1
	2020-03-09	-1		0.10	0.12	53	588	15	3	35	0.28	0.06	0.66	141	3	444	0.24	0.76	0.01
	2020-03-23	0	lockdown	0.03	0.03	9	78	1		8	0.11		0.89	5		73	0.06	0.94	
	2020-04-06	1		0.04	0.06	13	75	2	1	10	0.15	0.08	0.77	25	1	49	0.33	0.65	0.01
	2020-04-20	2		0.04	0.05	10	59	5		5	0.5		0.5	22		37	0.37	0.63	
	2020-05-04	3		0.02	0.03	7	41	2	1	4	0.29	0.14	0.57	17	7	17	0.41	0.41	0.17
	2020-05-18	4	post-lockdown	0.02	0.04	5	63	1		4	0.2		0.8	2		61	0.03	0.97	
	2020-06-01	5		0.01	0.00	1	1			1			1			1		1	
	2020-02-24	-2	before-lockdown	0.25	0.55	19	180	11	8		0.58	0.42		139	41		0.77		0.23

Supplementary Material 9: Trends in salience and sentiment for keywords per fortnight in Twitter data (Study 2).

Keyword	Fortnight starting	Fortnight to lockdown	Time window	Salience: Original tweets only	Salience: Incl. Retweets	Total N: Original tweets	Total N: Incl. Retweets	Count of sentiments, Original tweets only: Neu	Pos	Neg	Proportion of sentiments, Original tweets only: Neu	Pos	Neg	Count of sentiments, Incl. Retweets: Neu	Pos	Neg	Proportion of sentiments, Incl. Retweets: Neu	Neg	Pos
Michie	2020-03-09	-1	before-lockdown	0.09	0.08	51	372	26	24	1	0.51	0.47	0.02	200	170	2	0.54	0.01	0.46
	2020-03-23	0		0.06	0.04	17	96	14	2	1	0.82	0.12	0.06	91	4	1	0.95	0.01	0.04
	2020-04-06	1	lockdown	0.04	0.04	12	43	4	7	1	0.33	0.58	0.08	16	26	1	0.37	0.02	0.6
	2020-04-20	2		0.06	0.09	16	98	10	5	1	0.62	0.31	0.06	62	35	1	0.63	0.01	0.36
	2020-05-04	3		0.14	0.18	49	283	30	16	3	0.61	0.33	0.06	179	101	3	0.63	0.01	0.36
	2020-05-18	4	post-lockdown	0.14	0.52	29	800	23	5	1	0.79	0.17	0.03	782	17	1	0.98	0	0.02
	2020-06-01	5		0.29	0.20	39	87	37	2		0.95	0.05		84	3		0.97		0.03
Nudge (nudges, nudging, nudge theory, nudge strategy, paternalism, libertarian paternalism, paternalistic)	2020-02-24	-2	before-lockdown	0.04	0.02	3	5		2	1		0.67	0.33		4	1		0.2	0.8
	2020-03-09	-1		0.18	0.30	101	1460	32	18	51	0.32	0.18	0.5	77	52	1331	0.05	0.91	0.04
	2020-03-23	0		0.11	0.03	33	76	14	8	11	0.42	0.24	0.33	28	13	35	0.37	0.46	0.17
	2020-04-06	1	lockdown	0.05	0.02	16	28	2	5	9	0.12	0.31	0.56	8	5	15	0.29	0.54	0.18
	2020-04-20	2		0.12	0.10	33	109	9	6	18	0.27	0.18	0.55	26	11	72	0.24	0.66	0.1
	2020-05-04	3		0.08	0.05	29	76	13	5	11	0.45	0.17	0.38	38	13	25	0.5	0.33	0.17
	2020-05-18	4	post-lockdown	0.06	0.02	12	31	3	8	1	0.25	0.67	0.08	10	17	4	0.32	0.13	0.55
	2020-06-01	5		0.03	0.02	4	7	2	2		0.5	0.5		4	3		0.57		0.43
Choice architecture (choice architect)	2020-02-24	-2	before-lockdown	0.00	0.00	0	0												
	2020-03-09	-1		0.01	0.00	5	10	3	2		0.6	0.4		6	4		0.6		0.4
	2020-03-23	0		0.01	0.00	2	2	1	1		0.5	0.5		1	1		0.5		0.5
	2020-04-06	1	lockdown	0.00	0.00	0	0												
	2020-04-20	2		0.01	0.01	3	7		3			1			7				1
	2020-05-04	3		0.00	0.00	0	0												
	2020-05-18	4	post-lockdown	0.00	0.00	0	0												
	2020-06-01	5		0.00	0.00	0	0												
Psychologist	2020-02-24	-2	before-lockdown	0.01	0.00	1	1	1			1			1			1		
	2020-03-09	-1		0.06	0.37	32	1796	3	2	27	0.09	0.06	0.84	15	2	1779	0.01	0.99	0
	2020-03-23	0		0.02	0.02	5	54	2		3	0.4		0.6	7		47	0.13	0.87	
	2020-04-06	1	lockdown	0.01	0.00	2	4			2			1			4		1	
	2020-04-20	2		0.00	0.00	1	4	1			1			4			1		
	2020-05-04	3		0.01	0.00	3	4	1	2		0.33	0.67		2	2		0.5		0.5
	2020-05-18	4	post-lockdown	0.00	0.00	0	0												
	2020-06-01	5		0.00	0.00	0	0												
Psychology (psychologists, psychological science, psychological policy)	2020-02-24	-2	before-lockdown	0.14	0.32	11	107	5	6		0.45	0.55		63	44		0.59		0.41
	2020-03-09	-1		0.06	0.11	32	514	12	5	15	0.38	0.16	0.47	92	16	406	0.18	0.79	0.03
	2020-03-23	0		0.07	0.05	21	120	12	8	1	0.57	0.38	0.05	32	44	44	0.27	0.37	0.37
	2020-04-06	1	lockdown	0.06	0.13	19	155	7	8	4	0.37	0.42	0.21	48	86	21	0.31	0.14	0.55
	2020-04-20	2		0.09	0.17	23	185	6	14	3	0.26	0.61	0.13	29	153	3	0.16	0.02	0.83
	2020-05-04	3		0.08	0.10	29	165	10	15	4	0.34	0.52	0.14	39	91	35	0.24	0.21	0.55
	2020-05-18	4	post-lockdown	0.05	0.03	11	45	8	2	1	0.73	0.18	0.09	16	25	4	0.36	0.09	0.56
	2020-06-01	5		0.24	0.19	32	83	29	3		0.91	0.09		54	29		0.65		0.35
SPI-B	2020-02-24	-2	before-lockdown	0.00	0.00	0	0												
	2020-03-09	-1		0.00	0.00	0	0												
	2020-03-23	0		0.00	0.00	1	2	1			1			2			1		
	2020-04-06	1	lockdown	0.00	0.00	0	0												
	2020-04-20	2		0.00	0.00	0	0												
	2020-05-04	3		0.00	0.00	1	2			1			1			2		1	
	2020-05-18	4	post-lockdown	0.01	0.00	3	4	1		2	0.33		0.67	1		3	0.25	0.75	
	2020-06-01	5		0.00	0.00	0	0												
