# Supplementary material for: Lessons From the UK's Lockdown: Discourse on Behavioural Science in Times of COVID-19
Source: Front Psychol. 2021 Jun 17;12:647348. doi: 10.3389/fpsyg.2021.647348 (PMC8247580; doi:10.3389/fpsyg.2021.647348)
Supplement: Supplementary file 10 [file Data_Sheet_10.PDF]

Supplementary Material 10: Sentiments towards keywords separated by public policy application mentions for Twitter data (Study 2)

				**Total N**		**Count of sentiments Original tweets only**			**Count of sentiments Incl. Retweets**			**Proportion of sentiments Original tweets only**			**Proportion of sentiments Incl. Retweets**		
**Keyword**	**Public policy is mentioned**	**Fortnight starting**	**Fortnight to lockdown**	**Original tw**	**Incl. Retwe**	**neg**	**neu**	**pos**	**neg**	**neu**	**pos**	**neg**	**neu**	**pos**	**neg**	**neu**	**pos**
		2020-01-27	-4	0	0	0	0	0	0	0	0						
		2020-02-10	-3	0	0	0	0	0	0	0	0						
		2020-02-24	-2	23	43	1	12	10	1	28	14	0.04	0.52	0.43	0.02	0.65	0.33
		2020-03-09	-1	83	287	2	40	41	3	125	159	0.02	0.48	0.49	0.01	0.44	0.55
		2020-03-23	0	87	248	1	48	38	5	147	96	0.01	0.55	0.44	0.02	0.59	0.39
	no	2020-04-06	1	143	518	1	92	50	1	190	327	0.01	0.64	0.35	0.00	0.37	0.63
		2020-04-20	2	106	528	0	52	54	0	146	382	0.00	0.49	0.51	0.00	0.28	0.72
		2020-05-04	3	119	566	0	55	64	0	334	232	0.00	0.46	0.54	0.00	0.59	0.41
		2020-05-18	4	76	335	3	38	35	4	109	222	0.04	0.50	0.46	0.01	0.33	0.66
		2020-06-01	5	42	112	0	18	24	0	49	63	0.00	0.43	0.57	0.00	0.44	0.56
		2020-06-15	6	0	0	0	0	0	0	0	0						
Behaviour change		2020-06-29	7	0	0	0	0	0	0	0	0						
		2020-01-27	-4	0	0	0	0	0	0	0	0						
		2020-02-10	-3	0	0	0	0	0	0	0	0						
		2020-02-24	-2	0	0	0	0	0	0	0	0						
		2020-03-09	-1	10	41	2	0	8	2	0	39	0.20	0.00	0.80	0.05	0.00	0.95
		2020-03-23	0	5	16	1	1	3	1	1	14	0.20	0.20	0.60	0.06	0.06	0.88
	yes	2020-04-06	1	4	17	1	0	3	1	0	16	0.25	0.00	0.75	0.06	0.00	0.94
		2020-04-20	2	4	14	2	2	0	12	2	0	0.50	0.50	0.00	0.86	0.14	0.00
		2020-05-04	3	3	12	0	2	1	0	2	10	0.00	0.67	0.33	0.00	0.17	0.83
		2020-05-18	4	2	2	0	2	0	0	2	0	0.00	1.00	0.00	0.00	1.00	0.00
		2020-06-01	5	2	4	1	0	1	2	0	2	0.50	0.00	0.50	0.50	0.00	0.50
		2020-06-15	6	0	0	0	0	0	0	0	0						
		2020-06-29	7	0	0	0	0	0	0	0	0						
		2020-01-27	-4	0	0	0	0	0	0	0	0						
		2020-02-10	-3	0	0	0	0	0	0	0	0						
		2020-02-24	-2	3	3	1	1	1	1	1	1	0.33	0.33	0.33	0.33	0.33	0.33
		2020-03-09	-1	11	40	1	7	3	1	14	25	0.09	0.64	0.27	0.03	0.35	0.63
		2020-03-23	0	9	25	0	6	3	0	17	8	0.00	0.67	0.33	0.00	0.68	0.32
	no	2020-04-06	1	8	18	0	7	1	0	16	2	0.00	0.88	0.13	0.00	0.89	0.11
		2020-04-20	2	15	33	3	7	5	12	16	5	0.20	0.47	0.33	0.36	0.48	0.15
		2020-05-04	3	7	22	1	6	0	1	21	0	0.14	0.86	0.00	0.05	0.95	0.00
		2020-05-18	4	3	8	0	2	1	0	5	3	0.00	0.67	0.33	0.00	0.63	0.38
		2020-06-01	5	5	6	0	4	1	0	5	1	0.00	0.80	0.20	0.00	0.83	0.17
		2020-06-15	6	0	0	0	0	0	0	0	0						
Behavioural economics		2020-06-29	7	0	0	0	0	0	0	0	0						
(behavioural economists)		2020-01-27	-4	0	0	0	0	0	0	0	0						
		2020-02-10	-3	0	0	0	0	0	0	0	0						
		2020-02-24	-2	0	0	0	0	0	0	0	0						
		2020-03-09	-1	4	41	2	1	1	35	2	4	0.50	0.25	0.25	0.85	0.05	0.10
		2020-03-23	0	0	0	0	0	0	0	0	0						
	yes	2020-04-06	1	0	0	0	0	0	0	0	0						
		2020-04-20	2	1	1	1	0	0	1	0	0	1.00	0.00	0.00	1.00	0.00	0.00
		2020-05-04	3	1	1	1	0	0	1	0	0	1.00	0.00	0.00	1.00	0.00	0.00
		2020-05-18	4	0	0	0	0	0	0	0	0						
		2020-06-01	5	0	0	0	0	0	0	0	0						
		2020-06-15	6	0	0	0	0	0	0	0	0						
		2020-06-29	7	0	0	0	0	0	0	0	0						
		2020-01-27	-4	0	0	0	0	0	0	0	0						
		2020-02-10	-3	0	0	0	0	0	0	0	0						

		2020-02-24	-2	0	0	0	0	0	0	0	0						
		2020-03-09	-1	45	407	27	14	4	186	214	7	0.60	0.31	0.09	0.46	0.53	0.02
		2020-03-23	0	18	32	9	7	2	14	15	3	0.50	0.39	0.11	0.44	0.47	0.09
	no	2020-04-06	1	16	37	12	0	4	32	0	5	0.75	0.00	0.25	0.86	0.00	0.14
		2020-04-20	2	10	89	3	7	0	77	12	0	0.30	0.70	0.00	0.87	0.13	0.00
		2020-05-04	3	12	149	6	6	0	109	40	0	0.50	0.50	0.00	0.73	0.27	0.00
		2020-05-18	4	5	78	2	2	1	71	4	3	0.40	0.40	0.20	0.91	0.05	0.04
		2020-06-01	5	3	10	3	0	0	10	0	0	1.00	0.00	0.00	1.00	0.00	0.00
		2020-06-15	6	0	0	0	0	0	0	0	0						
Behavioural Insights Team (nudge unit)		2020-06-29	7	0	0	0	0	0	0	0	0						
		2020-01-27	-4	0	0	0	0	0	0	0	0						
		2020-02-10	-3	0	0	0	0	0	0	0	0						
		2020-02-24	-2	1	1	0	1	0	0	1	0	0.00	1.00	0.00	0.00	1.00	0.00
		2020-03-09	-1	44	358	23	20	1	296	36	26	0.52	0.45	0.02	0.83	0.10	0.07
		2020-03-23	0	19	645	14	4	1	581	55	9	0.74	0.21	0.05	0.90	0.09	0.01
	yes	2020-04-06	1	16	99	14	2	0	74	25	0	0.88	0.13	0.00	0.75	0.25	0.00
		2020-04-20	2	2	5	1	1	0	2	3	0	0.50	0.50	0.00	0.40	0.60	0.00
		2020-05-04	3	22	107	10	10	2	28	62	17	0.45	0.45	0.09	0.26	0.58	0.16
		2020-05-18	4	2	2	2	0	0	2	0	0	1.00	0.00	0.00	1.00	0.00	0.00
		2020-06-01	5	5	7	3	2	0	3	4	0	0.60	0.40	0.00	0.43	0.57	0.00
		2020-06-15	6	0	0	0	0	0	0	0	0						
		2020-06-29	7	0	0	0	0	0	0	0	0						
		2020-01-27	-4	0	0	0	0	0	0	0	0						
		2020-02-10	-3	0	0	0	0	0	0	0	0						
		2020-02-24	-2	19	112	0	9	10	0	64	48	0.00	0.47	0.53	0.00	0.57	0.43
		2020-03-09	-1	92	416	26	36	30	155	171	90	0.28	0.39	0.33	0.37	0.41	0.22
		2020-03-23	0	67	936	7	30	30	22	709	205	0.10	0.45	0.45	0.02	0.76	0.22
	no	2020-04-06	1	65	279	2	24	39	2	62	215	0.03	0.37	0.60	0.01	0.22	0.77
		2020-04-20	2	64	159	5	20	39	24	53	82	0.08	0.31	0.61	0.15	0.33	0.52
		2020-05-04	3	72	206	4	29	39	6	97	103	0.06	0.40	0.54	0.03	0.47	0.50
		2020-05-18	4	50	155	0	38	12	0	130	25	0.00	0.76	0.24	0.00	0.84	0.16
		2020-06-01	5	35	158	1	11	23	3	32	123	0.03	0.31	0.66	0.02	0.20	0.78
Behavioural science (behavioural sciences, behavioural policy, behavioural scientists)		2020-06-15	6	0	0	0	0	0	0	0	0						
		2020-06-29	7	0	0	0	0	0	0	0	0						
		2020-01-27	-4	0	0	0	0	0	0	0	0						
		2020-02-10	-3	0	0	0	0	0	0	0	0						
		2020-02-24	-2	3	8	0	1	2	0	1	7	0.00	0.33	0.67	0.00	0.13	0.88
		2020-03-09	-1	65	335	30	22	13	81	222	32	0.46	0.34	0.20	0.24	0.66	0.10
		2020-03-23	0	17	54	5	10	2	16	27	11	0.29	0.59	0.12	0.30	0.50	0.20
	yes	2020-04-06	1	12	38	6	4	2	29	6	3	0.50	0.33	0.17	0.76	0.16	0.08
		2020-04-20	2	9	58	4	4	1	38	18	2	0.44	0.44	0.11	0.66	0.31	0.03
		2020-05-04	3	19	119	14	2	3	100	5	14	0.74	0.11	0.16	0.84	0.04	0.12
		2020-05-18	4	18	51	1	15	2	4	29	18	0.06	0.83	0.11	0.08	0.57	0.35
		2020-06-01	5	1	2	1	0	0	2	0	0	1.00	0.00	0.00	1.00	0.00	0.00
		2020-06-15	6	0	0	0	0	0	0	0	0						
		2020-06-29	7	0	0	0	0	0	0	0	0						
		2020-01-27	-4	0	0	0	0	0	0	0	0						
		2020-02-10	-3	0	0	0	0	0	0	0	0						
		2020-02-24	-2	1	1	1	0	0	1	0	0	1.00	0.00	0.00	1.00	0.00	0.00
		2020-03-09	-1	21	277	11	10	0	53	224	0	0.52	0.48	0.00	0.19	0.81	0.00
		2020-03-23	0	6	94	2	3	1	25	68	1	0.33	0.50	0.17	0.27	0.72	0.01
	no	2020-04-06	1	4	18	0	4	0	0	18	0	0.00	1.00	0.00	0.00	1.00	0.00
		2020-04-20	2	1	1	0	1	0	0	1	0	0.00	1.00	0.00	0.00	1.00	0.00
		2020-05-04	3	3	5	1	2	0	1	4	0	0.33	0.67	0.00	0.20	0.80	0.00
		2020-05-18	4	1	23	0	1	0	0	23	0	0.00	1.00	0.00	0.00	1.00	0.00
		2020-06-01	5	1	39	0	1	0	0	39	0	0.00	1.00	0.00	0.00	1.00	0.00

		2020-06-15	6	0	0	0	0	0	0	0	0						
Behavioural scientist		2020-06-29	7	0	0	0	0	0	0	0	0						
		2020-01-27	-4	0	0	0	0	0	0	0	0						
		2020-02-10	-3	0	0	0	0	0	0	0	0						
		2020-02-24	-2	0	0	0	0	0	0	0	0						
		2020-03-09	-1	6	63	5	1	0	59	4	0	0.83	0.17	0.00	0.94	0.06	0.00
		2020-03-23	0	2	6	2	0	0	6	0	0	1.00	0.00	0.00	1.00	0.00	0.00
	yes	2020-04-06	1	0	0	0	0	0	0	0	0						
		2020-04-20	2	1	10	1	0	0	10	0	0	1.00	0.00	0.00	1.00	0.00	0.00
		2020-05-04	3	1	2	1	0	0	2	0	0	1.00	0.00	0.00	1.00	0.00	0.00
		2020-05-18	4	3	8	1	2	0	1	7	0	0.33	0.67	0.00	0.13	0.88	0.00
		2020-06-01	5	0	0	0	0	0	0	0	0						
		2020-06-15	6	0	0	0	0	0	0	0	0						
		2020-06-29	7	0	0	0	0	0	0	0	0						
		2020-01-27	-4	0	0	0	0	0	0	0	0						
		2020-02-10	-3	0	0	0	0	0	0	0	0						
		2020-02-24	-2	1	1	0	0	1	0	0	1	0.00	0.00	1.00	0.00	0.00	1.00
		2020-03-09	-1	26	337	20	4	2	213	122	2	0.77	0.15	0.08	0.63	0.36	0.01
		2020-03-23	0	5	31	4	1	0	26	5	0	0.80	0.20	0.00	0.84	0.16	0.00
	no	2020-04-06	1	8	13	7	0	1	12	0	1	0.88	0.00	0.13	0.92	0.00	0.08
		2020-04-20	2	6	26	1	5	0	4	22	0	0.17	0.83	0.00	0.15	0.85	0.00
		2020-05-04	3	1	14	0	1	0	0	14	0	0.00	1.00	0.00	0.00	1.00	0.00
		2020-05-18	4	2	56	1	1	0	54	2	0	0.50	0.50	0.00	0.96	0.04	0.00
		2020-06-01	5	0	0	0	0	0	0	0	0						
		2020-06-15	6	0	0	0	0	0	0	0	0						
Halpern		2020-06-29	7	0	0	0	0	0	0	0	0						
		2020-01-27	-4	0	0	0	0	0	0	0	0						
		2020-02-10	-3	0	0	0	0	0	0	0	0						
		2020-02-24	-2	0	0	0	0	0	0	0	0						
		2020-03-09	-1	27	251	15	11	1	231	19	1	0.56	0.41	0.04	0.92	0.08	0.00
		2020-03-23	0	4	47	4	0	0	47	0	0	1.00	0.00	0.00	1.00	0.00	0.00
	yes	2020-04-06	1	5	62	3	2	0	37	25	0	0.60	0.40	0.00	0.60	0.40	0.00
		2020-04-20	2	4	33	4	0	0	33	0	0	1.00	0.00	0.00	1.00	0.00	0.00
		2020-05-04	3	6	27	4	1	1	17	3	7	0.67	0.17	0.17	0.63	0.11	0.26
		2020-05-18	4	3	7	3	0	0	7	0	0	1.00	0.00	0.00	1.00	0.00	0.00
		2020-06-01	5	1	1	1	0	0	1	0	0	1.00	0.00	0.00	1.00	0.00	0.00
		2020-06-15	6	0	0	0	0	0	0	0	0						
		2020-06-29	7	0	0	0	0	0	0	0	0						
		2020-01-27	-4	0	0	0	0	0	0	0	0						
		2020-02-10	-3	0	0	0	0	0	0	0	0						
		2020-02-24	-2	18	179	0	11	7	0	139	40	0.00	0.61	0.39	0.00	0.78	0.22
		2020-03-09	-1	45	361	0	23	22	0	196	165	0.00	0.51	0.49	0.00	0.54	0.46
		2020-03-23	0	15	93	1	12	2	1	88	4	0.07	0.80	0.13	0.01	0.95	0.04
	no	2020-04-06	1	11	42	1	3	7	1	15	26	0.09	0.27	0.64	0.02	0.36	0.62
		2020-04-20	2	15	97	1	10	4	1	62	34	0.07	0.67	0.27	0.01	0.64	0.35
		2020-05-04	3	36	253	1	21	14	1	163	89	0.03	0.58	0.39	0.00	0.64	0.35
		2020-05-18	4	18	772	0	14	4	0	756	16	0.00	0.78	0.22	0.00	0.98	0.02
		2020-06-01	5	36	81	0	35	1	0	80	1	0.00	0.97	0.03	0.00	0.99	0.01
		2020-06-15	6	0	0	0	0	0	0	0	0						
Michie		2020-06-29	7	0	0	0	0	0	0	0	0						
		2020-01-27	-4	0	0	0	0	0	0	0	0						
		2020-02-10	-3	0	0	0	0	0	0	0	0						
		2020-02-24	-2	1	1	0	0	1	0	0	1	0.00	0.00	1.00	0.00	0.00	1.00
		2020-03-09	-1	6	11	1	3	2	2	4	5	0.17	0.50	0.33	0.18	0.36	0.45
		2020-03-23	0	2	3	0	2	0	0	3	0	0.00	1.00	0.00	0.00	1.00	0.00
	yes	2020-04-06	1	1	1	0	1	0	0	1	0	0.00	1.00	0.00	0.00	1.00	0.00

	yes	2020-04-20	2	1	1	0	0	1	0	0	1	0.00	0.00	1.00	0.00	0.00	1.00
		2020-05-04	3	13	30	2	9	2	2	16	12	0.15	0.69	0.15	0.07	0.53	0.40
		2020-05-18	4	11	28	1	9	1	1	26	1	0.09	0.82	0.09	0.04	0.93	0.04
		2020-06-01	5	3	6	0	2	1	0	4	2	0.00	0.67	0.33	0.00	0.67	0.33
		2020-06-15	6	0	0	0	0	0	0	0	0						
		2020-06-29	7	0	0	0	0	0	0	0	0						
		2020-01-27	-4	0	0	0	0	0	0	0	0						
		2020-02-10	-3	0	0	0	0	0	0	0	0						
		2020-02-24	-2	2	4	1	0	1	1	0	3	0.50	0.00	0.50	0.25	0.00	0.75
		2020-03-09	-1	52	1094	27	13	12	1017	33	44	0.52	0.25	0.23	0.93	0.03	0.04
		2020-03-23	0	25	55	8	9	8	27	15	13	0.32	0.36	0.32	0.49	0.27	0.24
	no	2020-04-06	1	10	18	5	1	4	8	6	4	0.50	0.10	0.40	0.44	0.33	0.22
		2020-04-20	2	25	56	11	8	6	20	25	11	0.44	0.32	0.24	0.36	0.45	0.20
		2020-05-04	3	22	56	5	12	5	8	35	13	0.23	0.55	0.23	0.14	0.63	0.23
		2020-05-18	4	9	19	0	1	8	0	2	17	0.00	0.11	0.89	0.00	0.11	0.89
ge (nudges, nudging, nudge theory, nudge strategy, paternalism, libertarian paternalism, paternalistic)		2020-06-01	5	3	5	0	2	1	0	4	1	0.00	0.67	0.33	0.00	0.80	0.20
		2020-06-15	6	0	0	0	0	0	0	0	0						
		2020-06-29	7	0	0	0	0	0	0	0	0						
		2020-01-27	-4	0	0	0	0	0	0	0	0						
		2020-02-10	-3	0	0	0	0	0	0	0	0						
		2020-02-24	-2	1	1	0	0	1	0	0	1	0.00	0.00	1.00	0.00	0.00	1.00
		2020-03-09	-1	49	366	24	19	6	314	44	8	0.49	0.39	0.12	0.86	0.12	0.02
		2020-03-23	0	8	21	3	5	0	8	13	0	0.38	0.63	0.00	0.38	0.62	0.00
	yes	2020-04-06	1	6	10	4	1	1	7	2	1	0.67	0.17	0.17	0.70	0.20	0.10
		2020-04-20	2	8	53	7	1	0	52	1	0	0.88	0.13	0.00	0.98	0.02	0.00
		2020-05-04	3	7	20	6	1	0	17	3	0	0.86	0.14	0.00	0.85	0.15	0.00
		2020-05-18	4	3	12	1	2	0	4	8	0	0.33	0.67	0.00	0.33	0.67	0.00
		2020-06-01	5	1	2	0	0	1	0	0	2	0.00	0.00	1.00	0.00	0.00	1.00
		2020-06-15	6	0	0	0	0	0	0	0	0						
		2020-06-29	7	0	0	0	0	0	0	0	0						
		2020-01-27	-4	0	0	0	0	0	0	0	0						
		2020-02-10	-3	0	0	0	0	0	0	0	0						
		2020-02-24	-2	1	1	0	1	0	0	1	0	0.00	1.00	0.00	0.00	1.00	0.00
		2020-03-09	-1	15	1001	13	2	0	991	10	0	0.87	0.13	0.00	0.99	0.01	0.00
		2020-03-23	0	3	9	1	2	0	2	7	0	0.33	0.67	0.00	0.22	0.78	0.00
	no	2020-04-06	1	1	2	1	0	0	2	0	0	1.00	0.00	0.00	1.00	0.00	0.00
		2020-04-20	2	1	4	0	1	0	0	4	0	0.00	1.00	0.00	0.00	1.00	0.00
		2020-05-04	3	3	4	0	1	2	0	2	2	0.00	0.33	0.67	0.00	0.50	0.50
		2020-05-18	4	0	0	0	0	0	0	0	0						
		2020-06-01	5	0	0	0	0	0	0	0	0						
		2020-06-15	6	0	0	0	0	0	0	0	0						
Psychologist		2020-06-29	7	0	0	0	0	0	0	0	0						
		2020-01-27	-4	0	0	0	0	0	0	0	0						
		2020-02-10	-3	0	0	0	0	0	0	0	0						
		2020-02-24	-2	0	0	0	0	0	0	0	0						
		2020-03-09	-1	17	795	14	1	2	788	5	2	0.82	0.06	0.12	0.99	0.01	0.00
		2020-03-23	0	2	45	2	0	0	45	0	0	1.00	0.00	0.00	1.00	0.00	0.00
	yes	2020-04-06	1	1	2	1	0	0	2	0	0	1.00	0.00	0.00	1.00	0.00	0.00
		2020-04-20	2	0	0	0	0	0	0	0	0						
		2020-05-04	3	0	0	0	0	0	0	0	0						
		2020-05-18	4	0	0	0	0	0	0	0	0						
		2020-06-01	5	0	0	0	0	0	0	0	0						
		2020-06-15	6	0	0	0	0	0	0	0	0						
		2020-06-29	7	0	0	0	0	0	0	0	0						
		2020-01-27	-4	0	0	0	0	0	0	0	0						
		2020-02-10	-3	0	0	0	0	0	0	0	0						

		2020-02-24	-2	10	101	0	5	5	0	63	38	0.00	0.50	0.50	0.00	0.62	0.38
		2020-03-09	-1	18	80	8	7	3	62	7	11	0.44	0.39	0.17	0.78	0.09	0.14
		2020-03-23	0	16	71	0	9	7	0	28	43	0.00	0.56	0.44	0.00	0.39	0.61
	no	2020-04-06	1	16	135	1	7	8	1	48	86	0.06	0.44	0.50	0.01	0.36	0.64
		2020-04-20	2	20	182	0	6	14	0	29	153	0.00	0.30	0.70	0.00	0.16	0.84
		2020-05-04	3	22	117	1	8	13	1	34	82	0.05	0.36	0.59	0.01	0.29	0.70
		2020-05-18	4	7	37	0	6	1	0	13	24	0.00	0.86	0.14	0.00	0.35	0.65
		2020-06-01	5	32	83	0	29	3	0	54	29	0.00	0.91	0.09	0.00	0.65	0.35
Psychology (psychologists, psychological science, psychological policy)		2020-06-15	6	0	0	0	0	0	0	0	0						
		2020-06-29	7	0	0	0	0	0	0	0	0						
		2020-01-27	-4	0	0	0	0	0	0	0	0						
		2020-02-10	-3	0	0	0	0	0	0	0	0						
		2020-02-24	-2	1	6	0	0	1	0	0	6	0.00	0.00	1.00	0.00	0.00	1.00
		2020-03-09	-1	14	434	7	5	2	344	85	5	0.50	0.36	0.14	0.79	0.20	0.01
		2020-03-23	0	5	49	1	3	1	44	4	1	0.20	0.60	0.20	0.90	0.08	0.02
	yes	2020-04-06	1	3	20	3	0	0	20	0	0	1.00	0.00	0.00	1.00	0.00	0.00
		2020-04-20	2	3	3	3	0	0	3	0	0	1.00	0.00	0.00	1.00	0.00	0.00
		2020-05-04	3	7	48	3	2	2	34	5	9	0.43	0.29	0.29	0.71	0.10	0.19
		2020-05-18	4	4	8	1	2	1	4	3	1	0.25	0.50	0.25	0.50	0.38	0.13
		2020-06-01	5	0	0	0	0	0	0	0	0						
		2020-06-15	6	0	0	0	0	0	0	0	0						
		2020-06-29	7	0	0	0	0	0	0	0	0						
		2020-01-27	-4	0	0	0	0	0	0	0	0						
		2020-02-10	-3	0	0	0	0	0	0	0	0						
		2020-02-24	-2	0	0	0	0	0	0	0	0						
		2020-03-09	-1	0	0	0	0	0	0	0	0						
		2020-03-23	0	0	0	0	0	0	0	0	0						
	no	2020-04-06	1	0	0	0	0	0	0	0	0						
		2020-04-20	2	0	0	0	0	0	0	0	0						
		2020-05-04	3	0	0	0	0	0	0	0	0						
		2020-05-18	4	1	1	1	0	0	1	0	0	1.00	0.00	0.00	1.00	0.00	0.00
		2020-06-01	5	0	0	0	0	0	0	0	0						
		2020-06-15	6	0	0	0	0	0	0	0	0						
SPI-B		2020-06-29	7	0	0	0	0	0	0	0	0						
		2020-01-27	-4	0	0	0	0	0	0	0	0						
		2020-02-10	-3	0	0	0	0	0	0	0	0						
		2020-02-24	-2	0	0	0	0	0	0	0	0						
		2020-03-09	-1	0	0	0	0	0	0	0	0						
		2020-03-23	0	1	2	0	1	0	0	2	0	0.00	1.00	0.00	0.00	1.00	0.00
	yes	2020-04-06	1	0	0	0	0	0	0	0	0						
		2020-04-20	2	0	0	0	0	0	0	0	0						
		2020-05-04	3	1	2	1	0	0	2	0	0	1.00	0.00	0.00	1.00	0.00	0.00
		2020-05-18	4	2	3	1	1	0	2	1	0	0.50	0.50	0.00	0.67	0.33	0.00
		2020-06-01	5	0	0	0	0	0	0	0	0						
		2020-06-15	6	0	0	0	0	0	0	0	0						
		2020-06-29	7	0	0	0	0	0	0	0	0						
