# Supplementary material for: Lessons From the UK's Lockdown: Discourse on Behavioural Science in Times of COVID-19
Source: Front Psychol. 2021 Jun 17;12:647348. doi: 10.3389/fpsyg.2021.647348 (PMC8247580; doi:10.3389/fpsyg.2021.647348)
Supplement: Supplementary file 11 [file Data_Sheet_11.PDF]

Supplementary Material 11: Sentiments towards public policy actors mentioned alongside behavioural science keywords for Twitter data (Study 2).

			Total count		Count of sentiments Original tweets only			Count of sentiments Incl. Retweets			Proportion of sentiments Original tweets only			Proportion of sentiments Incl. Retweets		
Keyword co-mentioned	Fortnight Starting	Fortnight to Lockdown	Original tweets only	Incl. Retweets	neg	neu	pos	neg	neu	pos	neg	neu	pos	neg	neu	pos
Behaviour change	2020-01-27	-4	0	0												
	2020-02-10	-3	0	0												
	2020-02-24	-2	0	0												
	2020-03-09	-1	10	41	4	3	3	30	6	5	0.40	0.30	0.30	0.73	0.15	0.12
	2020-03-23	0	5	16	1	2	2	12	2	2	0.20	0.40	0.40	0.75	0.13	0.13
	2020-04-06	1	4	17		4			17			1.00			1.00	
	2020-04-20	2	4	14	2		2	12		2	0.50		0.50	0.86		0.14
	2020-05-04	3	3	12	1	2		1	11		0.33	0.67		0.08	0.92	
	2020-05-18	4	2	2	1		1	1		1	0.50		0.50	0.50		0.50
	2020-06-01	5	2	4		2			4			1.00			1.00	
	2020-06-15	6	0	0												
	2020-06-29	7	0	0												
Behavioural Economics (behavioural economists)	2020-01-27	-4	0	0												
	2020-02-10	-3	0	0												
	2020-02-24	-2	0	0												
	2020-03-09	-1	4	41	4			41			1.00			1.00		
	2020-03-23	0	0	0												
	2020-04-06	1	0	0												
	2020-04-20	2	1	1	1			1			1.00			1.00		
	2020-05-04	3	1	1	1			1			1.00			1.00		
	2020-05-18	4	0	0												
	2020-06-01	5	0	0												
	2020-06-15	6	0	0												
	2020-06-29	7	0	0												
Behavioural Insights Team (nudge unit)	2020-01-27	-4	0	0												
	2020-02-10	-3	0	0												
	2020-02-24	-2	1	1	1			1			1.00			1.00		
	2020-03-09	-1	44	358	27	17		325	33		0.61	0.39		0.91	0.09	
	2020-03-23	0	19	645	16	3		594	51		0.84	0.16		0.92	0.08	
	2020-04-06	1	16	99	10	6		52	47		0.63	0.38		0.53	0.47	
	2020-04-20	2	2	5	2			5			1.00			1.00		
	2020-05-04	3	22	107	8	14		25	82		0.36	0.64		0.23	0.77	
	2020-05-18	4	2	2	2			2			1.00			1.00		
	2020-06-01	5	5	7	4	1		6	1		0.80	0.20		0.86	0.14	
	2020-06-15	6	0	0												
	2020-06-29	7	0	0												
Behavioural science (behavioural sciences, behavioural policy, behavioural scientists)	2020-01-27	-4	0	0												
	2020-02-10	-3	0	0												
	2020-02-24	-2	3	8		3			8			1.00			1.00	
	2020-03-09	-1	65	335	43	15	7	129	195	11	0.66	0.23	0.11	0.39	0.58	0.03
	2020-03-23	0	17	54	7	10		33	21		0.41	0.59		0.61	0.39	
	2020-04-06	1	12	38	5	7		25	13		0.42	0.58		0.66	0.34	
	2020-04-20	2	9	58	6	2	1	43	13	2	0.67	0.22	0.11	0.74	0.22	0.03
	2020-05-04	3	19	119	14	3	2	100	14	5	0.74	0.16	0.11	0.84	0.12	0.04
	2020-05-18	4	18	51	6	12		13	38		0.33	0.67		0.25	0.75	
	2020-06-01	5	1	2		1			2			1.00			1.00	
	2020-06-15	6	0	0												
	2020-06-29	7	0	0												
	2020-01-27	-4	0	0												

	2020-02-10	-3	0	0												
	2020-02-24	-2	0	0												
	2020-03-09	-1	6	63	4	2		58	5		0.67	0.33		0.92	0.08	
	2020-03-23	0	2	6	2			6			1.00			1.00		
Behavioural scientist	2020-04-06	1	0	0												
	2020-04-20	2	1	10	1			10			1.00			1.00		
	2020-05-04	3	1	2	1			2			1.00			1.00		
	2020-05-18	4	3	8	2	1		7	1		0.67	0.33		0.88	0.13	
	2020-06-01	5	0	0												
	2020-06-15	6	0	0												
	2020-06-29	7	0	0												
	2020-01-27	-4	0	0												
	2020-02-10	-3	0	0												
	2020-02-24	-2	0	0												
	2020-03-09	-1	27	251	17	10		235	16		0.63	0.37		0.94	0.06	
	2020-03-23	0	4	47	2	2		2	45		0.50	0.50		0.04	0.96	
Halpern	2020-04-06	1	5	62	2	3		36	26		0.40	0.60		0.58	0.42	
	2020-04-20	2	4	33	3	1		31	2		0.75	0.25		0.94	0.06	
	2020-05-04	3	6	27	2	4		10	17		0.33	0.67		0.37	0.63	
	2020-05-18	4	3	7	3			7			1.00			1.00		
	2020-06-01	5	1	1	1			1			1.00			1.00		
	2020-06-15	6	0	0												
	2020-06-29	7	0	0												
	2020-01-27	-4	0	0												
	2020-02-10	-3	0	0												
	2020-02-24	-2	1	1		1			1			1.00			1.00	
	2020-03-09	-1	6	11	4	2		8	3		0.67	0.33		0.73	0.27	
	2020-03-23	0	2	3	1	1		1	2		0.50	0.50		0.33	0.67	
Michie	2020-04-06	1	1	1			1			1			1.00			1.00
	2020-04-20	2	1	1	1			1			1.00			1.00		
	2020-05-04	3	13	30	8	3	2	11	6	13	0.62	0.23	0.15	0.37	0.20	0.43
	2020-05-18	4	11	28	6	5		13	15		0.55	0.45		0.46	0.54	
	2020-06-01	5	3	6	1	2		1	5		0.33	0.67		0.17	0.83	
	2020-06-15	6	0	0												
	2020-06-29	7	0	0												
	2020-01-27	-4	0	0												
	2020-02-10	-3	0	0												
Nudge	2020-02-24	-2	1	1			1			1			1.00			1.00
(nudges, nudging,	2020-03-09	-1	49	366	37	11	1	349	16	1	0.76	0.22	0.02	0.95	0.04	0.00
nudge theory,	2020-03-23	0	8	21	3	5		9	12		0.38	0.63		0.43	0.57	
nudge strategy,	2020-04-06	1	6	10	4	2		7	3		0.67	0.33		0.70	0.30	
paternalism,	2020-04-20	2	8	53	4	4		47	6		0.50	0.50		0.89	0.11	
libertarian	2020-05-04	3	7	20	5	2		11	9		0.71	0.29		0.55	0.45	
paternalism,	2020-05-18	4	3	12	2	1		10	2		0.67	0.33		0.83	0.17	
paternalistic)	2020-06-01	5	1	2		1			2			1.00			1.00	
	2020-06-15	6	0	0												
	2020-06-29	7	0	0												
	2020-01-27	-4	0	0												
	2020-02-10	-3	0	0												
	2020-02-24	-2	0	0												
	2020-03-09	-1	17	795	14	2	1	788	6	1	0.82	0.12	0.06	0.99	0.01	0.00
	2020-03-23	0	2	45	1	1		1	44		0.50	0.50		0.02	0.98	
psychologist	2020-04-06	1	1	2	1			2			1.00			1.00		
	2020-04-20	2	0	0												
	2020-05-04	3	0	0												
	2020-05-18	4	0	0												

	2020-06-01	5	0	0												
	2020-06-15	6	0	0												
	2020-06-29	7	0	0												
	2020-01-27	-4	0	0												
	2020-02-10	-3	0	0												
	2020-02-24	-2	1	6		1			6			1.00			1.00	
psychology (psychologists, psychological science, psychological policy)	2020-03-09	-1	14	434	8	5	1	362	69	3	0.57	0.36	0.07	0.83	0.16	0.01
	2020-03-23	0	5	49	1	3	1	1	47	1	0.20	0.60	0.20	0.02	0.96	0.02
	2020-04-06	1	3	20	3			20			1.00			1.00		
	2020-04-20	2	3	3	3			3			1.00			1.00		
	2020-05-04	3	7	48	2	2	3	3	32	13	0.29	0.29	0.43	0.06	0.67	0.27
	2020-05-18	4	4	8	2	2		5	3		0.50	0.50		0.63	0.38	
	2020-06-01	5	0	0												
	2020-06-15	6	0	0												
	2020-06-29	7	0	0												
	2020-01-27	-4	0	0												
	2020-02-10	-3	0	0												
	2020-02-24	-2	0	0												
	2020-03-09	-1	0	0												
	2020-03-23	0	1	2		1			2			1.00			1.00	
SPI-B	2020-04-06	1	0	0												
	2020-04-20	2	0	0												
	2020-05-04	3	1	2	1			2			1.00			1.00		
	2020-05-18	4	2	3	2			3			1.00			1.00		
	2020-06-01	5	0	0												
	2020-06-15	6	0	0												
	2020-06-29	7	0	0												
