# Supplementary material for: Lessons From the UK's Lockdown: Discourse on Behavioural Science in Times of COVID-19
Source: Front Psychol. 2021 Jun 17;12:647348. doi: 10.3389/fpsyg.2021.647348 (PMC8247580; doi:10.3389/fpsyg.2021.647348)
Supplement: Supplementary file 12 [file Data_Sheet_12.PDF]

7.12 Supplementary Materials 12: Comparison of direct quotes, indirect quotes and having been spoken about for key actors in the pre- during and post-lockdown time period in print media.

	Quoted	Michie	Halpern	SPI-B	BIT	Reicher
Pre-lockdown	Direct quotes	6	1			
	Indirect quotes	2	1			
	Spoken about		5		1	
	**Total**	**8**	**7**		**1**	
During lockdown	Direct quotes	16	2	12		3
	Indirect quotes	2		4		
	Spoken about			11		
	**Total**	**18**	**2**	**27**		**3**
Post-lockdown	Direct quotes	21	3	5		29
	Indirect quotes	7		1		9
	Spoken about	5	2	11		14
	**Total**	**33**	**5**	**17**		**52**
Total	Direct quotes	43	6	17		32
	Indirect quotes	11	1	5		9
	Spoken about	5	7	22	1	14
	**Total**	**59**	**13**	**45**	**3**	**55**

Note: Comparison of direct quotes, indirect quotes and having been spoken about for key actors in the pre- during and post-lockdown time period. 'Direct quotes' refer to opinion context where named actors are discussed with entire or parts of the speech-based content, taken from an interviews or tweets, reported with quotation marks. 'Indirect quotes' refer to opinion contexts where named actor interview or tweet content was reported in the form of indirect speech. 'Spoken about' refers to opinion contexts where the actors were mentioned without any reported speech, neither in direct quotes or indirect speech form.
